# A path to gigantism: Three‐dimensional study of the sauropodomorph limb long bone shape variation in the context of the emergence of the sauropod bauplan

**DOI:** 10.1111/joa.13646

**Published:** 2022-03-06

**Authors:** Rémi Lefebvre, Alexandra Houssaye, Heinrich Mallison, Raphaël Cornette, Ronan Allain

**Affiliations:** ^1^ Mécanismes Adaptatifs et Évolution, UMR 7179, MNHN, CNRS Muséum National d'Histoire Naturelle Paris France; ^2^ CeNak Hamburg Hamburg Germany; ^3^ Palaeo3D Rain Germany; ^4^ Institut de Systématique, Évolution, Biodiversité, UMR7205, MNHN, CNRS, SU, EPHE, UA Muséum National d'Histoire Naturelle Paris France; ^5^ Centre de Recherche en Paléontologie – Paris, UMR 7207, MNHN, CNRS, SU Muséum National d'Histoire Naturelle Paris France

**Keywords:** Dinosauria, functional morphology, gigantism, graviportality, phylomorphospace

## Abstract

Sauropodomorph dinosaurs include the largest terrestrial animals that ever lived on Earth. The early representatives of this clade were, however, relatively small and partially to totally bipedal, conversely to the gigantic and quadrupedal sauropods. Although the sauropod bauplan is well defined, notably by the acquisition of columnar limbs, the evolutionary sequence leading to its emergence remains debated. Here, we aim to tackle this evolutionary episode by investigating shape variation in the six limb long bones for the first time using three‐dimensional geometric morphometrics. The morphological features of the forelimb zeugopod bones related to the sauropod bauplan tend to appear abruptly, whereas the pattern is more gradual for the hindlimb zeugopod bones. The stylopod bones tend to show the same pattern as their respective zeugopods. The abrupt emergence of the sauropod forelimb questions the locomotor abilities of non‐sauropodan sauropodomorphs inferred as quadrupeds. Features characterizing sauropods tend to corroborate a view of their locomotion mainly based on stylopod retraction. An allometric investigation of the shape variation in accordance with size highlight differences in hindlimb bone allometries between the sauropods and the non‐sauropodan sauropodomorphs. These differences notably correspond to an unexpected robustness decrease trend in the sauropod hindlimb zeugopod. In addition to forelimb bones that appear to be proportionally more gracile than in non‐sauropodan sauropodomorphs, sauropods may have relied on limb architecture and features related to the size increase, rather than general robustness, to deal with the role of weight‐bearing.

## Graphical abstract

Our study investigates the limb long bone shape variation of a large sample of sauropodomorph dinosaurs in the context of the emergence of the sauropod bauplan. We highlight a differential emergence of sauropod‐related features, that appear abruptly in the forelimb bones and more gradually in the hindlimb ones. The study of the allometry shows unexpected size‐related differences of variation between non‐sauropodan sauropodmorphs and sauropods in the hindlimb zeugopod.

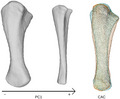

## 1 INTRODUCTION

**FIGURE 1:**
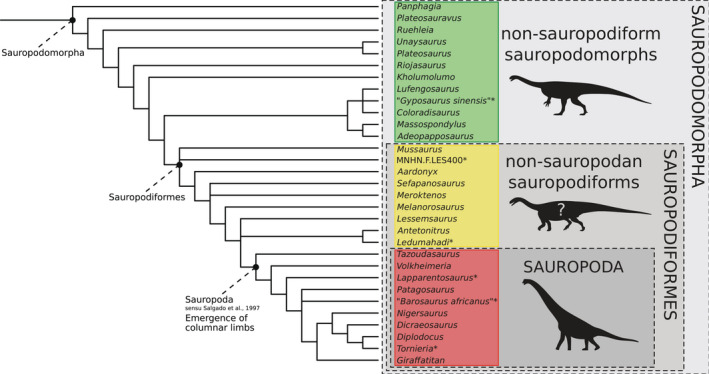
Phylogeny of the sauropodomorph dinosaurs used in this study. On the right, the Venn diagram in grey gives an overview of the taxonomy of the group, with clades in capital letters. The taxa belonging to the clade Sauropoda (in red) are examined in this study compared to taxa belonging to non‐sauropodiform sauropodomorphs (in green) and non‐sauropodan sauropodiforms (in yellow) grades. Based on phylogenies of Holwerda and Pol (2018) and Peyre de Fabrègues and Allain (2019). Taxa marked with an asterisk were added informally to the consensus of these two phylogenies (see Section 2). Silhouettes by Scott Hartman

Sauropodomorph dinosaurs include the largest terrestrial animals that ever lived on Earth (Rauhut et al., 2011; Sander & Clauss, 2008; Sander et al., 2011). Although these iconic long‐necked quadrupedal animals reached gigantic body sizes and masses around the node Sauropoda (Figure 1), the first representatives of this group, that is the non‐sauropodan sauropodomorphs (green and yellow clusters in Figure 1, formerly known as ‘prosauropods’ Sereno, 2007; also known as ‘basal sauropodomorphs’, Martinez & Alcober, 2009; Langer et al., 2010; see Bronzati, 2017), were small to moderately large animals and mostly inferred to be partial to exclusively bipeds (Bonnan & Senter, 2007; Bonnan & Yates, 2007; Chapelle et al., 2020; Mallison, 2010a; McPhee et al., 2014; Otero et al., 2017, 2019). Conversely, sauropods (sensu Salgado et al., 1997; Figure 1) are obligatory quadrupeds: the appearance and diversification of the group Sauropoda within Sauropodomorpha is thus linked to the emergence of a new bauplan (or body plan; Rauhut et al., 2011; Sander et al., 2011).

Numerous skeletal features characterize the sauropod bauplan. Key among them is the acquisition of columnar limbs, that is subvertically oriented limbs (Osborn, 1900), with limited flexion during rest and locomotion (Hildebrand, 1982; Wilson, 2005a; Wilson & Sereno, 1998). This limb orientation manages heavy weight efficiently (Hildebrand, 1982). Some other morphological features associated with the sauropod bauplan are the straight long axis of most limb bones, particularly the femur (Salgado et al., 1997; Wilson, 2005a; Wilson & Sereno, 1998) as well as several reductions of processes, notably the olecranon process, the cnemial crest and the fourth and lesser trochanters (Carrano, 2005; Upchurch, 1998; Wilson & Sereno, 1998), implying reduced locomotor abilities (Carrano, 1999, 2005). The forelimb is considerably modified compared to non‐sauropodan sauropodomorphs, as it is globally elongated and bears several characteristic features such as a low deltopectoral crest and a deep radial fossa (Wilson & Sereno, 1998; see McPhee & Choiniere, 2018 for a recent review).

The manus is digitigrade with a small number of phalanges (Allain & Aquesbi, 2008; Carrano, 2005; Goussard, 2009; Wilson, 2005a). The pelvis of sauropods is also characterized by an ilium anteroposteriorly developed with a low ischial peduncle (Carrano, 2005; Salgado et al., 1997; Wilson & Sereno, 1998) and an ischium subequal to longer than the pubis (Wilson & Sereno, 1998).

Some other features characterize sauropods but are not as tightly linked to the emergence of the clade. For instance, sauropods have a minimum of four sacral vertebrae, but this number is also found in some non‐sauropodan sauropodomorphs (Otero & Pol, 2013; Pol et al., 2011; Wang et al., 2017). The semi‐tubular manus, found in the vast majority of sauropods, appears later in the evolution of the clade (Allain & Aquesbi, 2008; Bonnan, 2003; Bonnan & Yates, 2007; Goussard, 2009; Wilson, 2005b; Wilson & Sereno, 1998), whereas the timing of appearance of the functionally plantigrade pes (i.e. supported by a fleshy pad) within sauropods is not clear (Jannel et al., 2019; Wilson, 2005b).

The evolution of gigantism within sauropodomorphs, that is reaching a high body size and multi‐ton body mass, is traditionally thought to be tightly linked to the evolution of columnar limbs (Rauhut et al., 2011; Sander et al., 2011; Wilson & Sereno, 1998). However, recent studies on newly described material of large non‐sauropodan sauropodomorphs tend to draw a different narrative. On the basis of osteology (Apaldetti et al., 2018; McPhee et al., 2018), histological features, reflecting an accelerated cyclical growth (Apaldetti et al., 2018), and limb circumferences (McPhee et al., 2018), these studies favour a scenario implying an early trend towards gigantism before the emergence of the sauropod bauplan. These non‐sauropodan sauropodomorphs closely related to sauropods would have reached body masses of a dozen of tons, comparable to early sauropods (e.g. Vulcanodontidae, *Shunosaurus*; Apaldetti et al., 2018; McPhee et al., 2018; Sander & Lallensack, 2018). Moreover, the acquisition of quadrupedality would have been anterior to the appearance of the columnar limbs in sauropods (McPhee et al., 2018). However, columnar limbs are still supposed to be the innovation that allowed sauropods to diversify in very diverse forms reaching a range of extreme gigantism, that is exceeding several dozens of tons, masses never reached by non‐sauropodan sauropodomorphs (Sander et al., 2011; Sander & Lallensack, 2018). In addition, caution is needed for body mass estimations as the methods used are sensitive to the inferred posture, which is estimated with uncertainties for numerous non‐sauropodan‐sauropodomorphs (Campione et al., 2014; Campione & Evans, 2012; Peyre de Fabrègues & Allain, 2019).

The variation in the shape of the limb bones and their scaling in relation to body size during the emergence of the sauropod bauplan, although investigated in numerous studies, were mostly explored by qualitative and linear measurement approaches (Allain & Aquesbi, 2008; Bonnan, 2003, 2005; Bonnan & Yates, 2007; Cooper, 1984; McPhee & Choiniere, 2018; Otero & Pol, 2013; Rauhut et al., 2011; Wilson & Sereno, 1998). Meanwhile, the power of biological studies investigating quantitatively the variation of shape has been enhanced through the advances in geometric morphometrics (GM; Adams et al., 2013; Mitteroecker & Gunz, 2009; Rohlf & Marcus, 1993). With its successive developments, this method now offers a robust framework to quantify the whole three‐dimensional (3D) shape of a specimen, notably by the use of 3D sliding semilandmarks (Gunz et al., 2005; Gunz & Mitteroecker, 2013), and to link shape variation with factors such as size (e.g. Klingenberg, 2016; Mitteroecker et al., 2004; Monteiro, 1999). Previous GM investigations of sauropodomorph limb bones were mostly performed in two‐dimensional (2D), using anatomical landmarks (Bonnan, 2004, 2007; Canudo & Cuenca‐Bescós, 2004; Yates et al., 2010; Ullmann et al., 2017; a 3D study was performed on non‐sauropodan sauropodomorph humeri in Staunton, unpublished dissertation), and only one study investigated both non‐sauropodan sauropodomorphs and sauropods in the context of the evolution of the sauropod bauplan (Yates et al., 2010). However, 2D analyses constrain to study the sampled specimens in only one view, thereby sometimes inducing inaccurate capture of the shape of objects (Cardini & Chiapelli, 2020). Moreover, limb bones can show large regions, such as shafts, lacking anatomical landmarks, and can thus only be correctly digitized by a sliding semilandmark approach. Recently, 3D GM studies (Lefebvre et al., 2020, investigating the biological and taphonomic variation in *Plateosaurus*, and Páramo et al., 2020, 2022 focusing on titanosaurs in a systematic context) have been performed on sauropodomorph limb long bones using anatomical landmarks and sliding semilandmarks, showing the potential of applying this method to the study of sauropodomorph evolution. Applied to the study of sauropodomoprh gigantism, 3D GM appears as a powerful framework allowing to study the allometry, that is the investigation of size and its consequences on shape variation (see Gould, 1966; Klingenberg, 2016). Indeed, the substantial differences in bauplan observed between the columnar sauropods and the non‐columnar sauropodomorphs noted by many authors (e.g. Apaldetti et al., 2018; Carrano, 2005; McPhee et al., 2018; Rauhut et al., 2011; Wilson, 2005a; Wilson & Sereno, 1998; Yates et al., 2010) may have induced (or have been concomitant with) a different management of size and mass increase in both groups. It is thus plausible that the allometric patterns of shape variation are different between columnar and non‐columnar sauropodomorphs.

Here we propose the first investigation, through the use of 3D GM, of the shape variation of the forelimb and hindlimb long bones occurring in relation to the emergence of the sauropod bauplan. The herein study (1) tests if the shape associated with the columnar limb architecture of the sauropod bauplan is distinguishable in a quantitative framework, (2) investigates the evolutionary pattern of the emergence of this architecture and the associated morphological features and (3) estimates the impact of allometry in the shape variation associated with this evolutionary episode, taking limb architecture into account.

The herein investigation of the six limb long bones in a quantitative framework should provide an integrative point of view of the evolution of the limb bauplan that permitted the extreme form of gigantism reached in this group.

## 2 MATERIALS AND METHODS

### 2.1 Materials and digitization

We studied the forelimb and hindlimb stylopod and zeugopod bones of a large sample of sauropodomorph dinosaurs (Table S1). A data set of 141 bones (20 humeri, 21 radii, 29 ulnae, 22 femora, 25 tibiae and 24 fibulae) sampled out of a total of 584 examined bones was used in our analysis. The sampled bones were digitized into 3D models using: (1) a surface scanner Artec EVA and the software Artec Studio 12 (Artec 3D, 2018), (2) Computed tomography scan data (Mallison, 2010a) and (3) photogrammetry and the software Agisoft Photoscan Pro (Agisoft LLC, 2018) or Reality Capture (Capturing Reality s.r.o., 2018), following recommendations of Mallison and Wings (2014) and Fau et al. (2016). In order to facilitate the analyses, the 3D models were decimated using the software Meshlab (Cignoni et al., 2008) or GOMinspect (GOM GmbH, 2018) when they were above the limit of 500,000 faces. The left bones were symmetrized arbitrarily on the right side for the purpose of the analysis using the same software. The complete specimens that were found in several parts at the moment of the accession to the collections were digitized as such and virtually merged using the software Blender (The Blender Foundation, 2017). Also, some bones were presenting missing parts for which the original complete morphology could easily be estimated. For them, we filled the gaps or reconstructed some missing parts (i.e. interpolation of well‐constrained parts of shaft or curves) using the software Geomagic (3D Systems, 2017).

### 2.2 Management of taphonomy

As our study intends to investigate the anatomical variation among a sample of fossil taxa, it is critical to take taphonomy into account (Hedrick & Dodson, 2013; Lefebvre et al., 2020). Indeed, the morphology of the bones is the result of two histories: the biological history of the living organism and the taphonomic history of the remains, from the death of the animal until its discovery. The latter involves deformations as well as incompleteness linked to breaks or abrasions. Thus, it is critical to apply a strategy of management of this bias. As the total number of specimens examined in this study was large (see Section 2.1, Table S1), and given the examined large taxonomic scale, we selected only the best‐preserved specimens available for each type of bone, in order to minimize a priori the impact of taphonomy in our sample (Lefebvre et al., 2020). Therefore, we graded each bone with regard to the following areas: the shaft, the proximal and distal ends, the deltopectoral crest (in humeri) and the fourth trochanter (in femora). We evaluated the apparent impact of taphonomy for each of these areas. For our analyses, we included only those bones that displayed minor to at most moderate taphonomic influence (Table S1). Sampled bones showing the anatomically aberrant shape and/or missing significant parts were thus excluded from the geometric morphometrics analyses.

### 2.3 Phylogenetic framework

Sauropodomorph phylogeny has been intensively studied since the first cladistic analyses involving this group (e.g. Benton et al., 2000; Galton, 1990; Galton & Upchurch, 2004; Gauthier, 1986; Langer et al., 1999, 2010, 2019; Martinez et al., 2012; Martinez & Alcober, 2009; McPhee et al., 2014; McPhee & Choiniere, 2018; Müller, Langer, Bronzati, et al., 2018; Peyre de Fabrègues et al., 2015; Peyre de Fabrègues & Allain, 2019; Pol & Powell, 2007; Sereno, 1999, 2007; Upchurch et al., 2007; Wilson & Sereno, 1998; Yates, 2003, 2007; Yates et al., 2010; Yates & Kitching, 2003; Zhang et al., 2018). However, several definitions of the groups composing this clade remain strongly debated. Although the monophyly (e.g. Galton, 1990; Galton & Upchurch, 2004; Sereno, 1999) or paraphyly (e.g. Pol et al., 2011; Pol & Powell, 2007; Yates, 2003, 2007) of the traditional group ‘Prosauropoda’ is still discussed, its paraphyly tended recently to become more dominantly accepted (although its structure is still debated; see Sereno, 2007; Upchurch et al., 2007; Peyre de Fabrègues et al., 2015). Hence, the current view of the clade Sauropodomorpha tends to define three groups of interests. Among the paraphyletic non‐sauropodan sauropodomorphs, one of those is the non‐sauropodiform sauropodomorphs (Figure 1). It is a paraphyletic assemblage of sauropodomorph taxa relatively far‐related to sauropods and are classically inferred as facultative to obligate bipeds (i.e. consensually containing *Plateosaurus* and *Massospondylus*, as well as some other unstable taxa, e.g. *Riojasaurus*; Sereno, 2007; Langer et al., 2010; Müller, 2020; Müller, Langer, & Dias‐da‐Silva, 2018; Peyre de Fabrègues & Allain, 2019; McPhee et al., 2020). On the other hand, some ‘near‐sauropod’ taxa (i.e. non‐sauropodan‐sauropodiforms; see Figure 1) correspond to non‐sauropodan sauropodomorphs closer to Sauropoda than to the non‐sauropodiform sauropodomorphs (McPhee et al., 2014; Sereno, 2007). They are traditionally viewed as showing several successive morphological innovations, assumed to be a progressive transition to the sauropod bauplan (Bonnan & Yates, 2007; McPhee et al., 2014; Otero et al., 2015; Otero & Pol, 2013; Yates et al., 2010). The phylogenetic position of many of these taxa is, however, lacking a stable consensus (e.g. McPhee et al., 2018; Otero et al., 2015; Peyre de Fabrègues & Allain, 2019). The last group, the clade Sauropoda, can take different definitions (Salgado et al., 1997; Sereno, 2007; Wilson & Sereno, 1998; Yates, 2007). Following the different definitions, several Sauropodiformes taxa fall within or outside Sauropoda (McPhee & Choiniere, 2018; Peyre de Fabrègues et al., 2015). In the least‐inclusive definition proposed by Salgado et al. (1997), only the taxa‐possessing columnar limbs fall within Sauropoda, This node‐based definition is the most stable and conservative compared to the alternative more unstable stem‐based definitions, lacking a similarly strong consensus (e.g. Wilson & Sereno, 1998; Sereno, 2007; Yates, 2007; see Peyre de Fabrègues et al., 2015; McPhee et al., 2018 for a comparative discussion of the definitions). Given the scope of our study, which focused on the emergence of the columnar‐limbed sauropods, we use here the definition of Sauropoda given by Salgado et al. (1997).

Despite the thorough effort to reconstruct the phylogenetic relationships of sauropodomorph dinosaurs, no robust consensus has emerged about the relationships of non‐sauropodan sauropodomorphs (see Sereno, 2007; Upchurch et al., 2007; Peyre de Fabrègues et al., 2015) and of non‐neosauropodan sauropods (see Holwerda & Pol, 2018). Consequently, the investigation of the phylogenetic signal in this study is tentative but will permit to test the robustness of several assumptions related to the emergence of the sauropod bauplan, e.g. the reliability of the distinction of non‐sauropodan sauropodiforms as showing features involved in the hypothesized scenario of a progressive evolution towards the sauropod bauplan, or also the quantification of the distinction between columnar and non‐columnar limbs.

To do so, an informal consensus tree (Figure 1) was built following Peyre de Fabrègues & Allain (2019, Figure S2) and Holwerda and Pol (2018). Taxa missing from this tree were informally added following the groups in the tree to which they are the most closely related. We chose this approach to add these taxa given the strong conflicts existing between the different phylogenetic analyses necessary to cover all the relationships of the analyzed taxa. The phylogenetic positions of *Gyposaurus sinensis* (often seen as a junior synonym of *Lufengosaurus*; Galton & Upchurch, 2004) and *Lapparentosaurus* were added following Pol et al. (2011), *Ledumahadi* following McPhee et al. (2018), *Tornieria* following Remes (2006) and of MNHN.F.LES400 following Peyre de Fabrègues, 2016; C. Peyre de Fabrègues, personal communication, 2020). The taxonomy and *a fortiori*, the phylogenetic relationships of the specimens attributed to *Barosaurus africanus*, are unclear since Remes (2009) established that the remains of this taxon from the Tendaguru Formation constitute an assemblage of indeterminate specimens. As all taxa discovered in the Tendaguru formation are sauropods belonging to Neosauropoda, Turiasauria or Mamenchisauridae (Mannion et al., 2019), we tentatively placed all the specimens referred to *B. africanus*, as well as an indeterminate radius and fibula, at the base of a polytomy regrouping all these taxa in the topology of Peyre de Fabrègues and Allain (2019).

### 2.4 Landmark acquisition

A set of anatomical landmarks was defined to digitize each type of bone (Table S2). We used sliding semilandmarks of curves and surfaces (Gunz & Mitteroecker, 2013) to capture the overall form of the bones, since the number of anatomical landmarks is, especially in this study, scarce for limb bones, compared to studies based on skulls. Anatomical landmarks and sliding semilandmarks of curves were acquired by the same operator (R.L.) using the software IDAV Landmark (Wiley et al., 2005). For each type of bone, a repeatability procedure was performed by acquiring the anatomical landmarks 10 times for three closely related specimens. A generalized procrustes analysis (GPA; see Gower, 1975; Rohlf & Slice, 1990; see also Section 2.5), followed by a principal component analysis (PCA; see Section 2.5) permitted to verify (Figure S1) that the intra‐individual variability (measurement error) was lower than inter‐individual variability (morphological variation). Sliding semilandmarks of the curve were resampled and evenly spaced using the function from Botton‐Divet et al. (2016). The surface‐sliding semilandmarks were warped on 3D models thanks to a template, that is a 3D model selected among each type of sampled bones. To do so, the sliding semilandmarks of the surface were placed manually on the template with the software IDAV Landmark. They were then warped using the ‘placePatch’ function of the Morpho package version 2.8 (Schlager, 2017) in R version 3.5.1 (R Core Team, 2018). A sliding procedure was performed on the sliding semilandmarks of curves and surfaces following the protocol of Gunz et al. (2005), minimizing the bending energy of a Thin‐Plate Spline, first between each specimen and the template (‘relaxLM’ function in Morpho; iterated five times), then between the result for each specimen and the consensus of the dataset (‘slider3d’ function in Morpho; iterated five times).

### 2.5 Three‐dimensional geometric morphometrics

Once slid, the resulting landmark configurations are superimposed with a GPA. This procedure removes the size, spatial position and orientation of the specimens. A PCA is performed on each of these data sets. This ordination analysis reorganizes the multivariate variation of the data set by maximizing the explained variation in a reduced number of new uncorrelated axes, the principal components (PC). We performed a projection of the phylogenetic relationships of our taxa in the multivariate shape space of the PCA, resulting in a phylomorphospace. This approach permits to visualize the distribution of the specimens in the morphospace, given their assumed phylogenetic relationships, by mapping an inferred tree. GPA was performed using the ‘gpagen’ function of the R package geomorph version 3.3.1 (Adams et al., 2020). The phylomorphospaces were obtained using ‘gm.prcomp’ function of the same package. PC1 and PC2 extreme landmark conformations were mapped on the meanshape and exported in 3D models using the ‘vcgPlywrite’ function of the Rvcg R package (Schlager, 2017). The 3D model used to map the extreme conformations was chosen among the sample by selecting the specimen showing excellent preservation and the fewest particular morphological features, in order to avoid interpreting artefactual structures. The exported 3D models representing the negative and the positive extremal conformations were compared by superimposition in Meshlab. A complementary comparison with the visualization obtained using the ‘DeformGrid3D’ function in Morpho was also performed. This function compares the two extreme conformations by superimposing them and by linking the geometrically homologous landmarks, without involving any 3D model deformation. Therefore, descriptions of variations of each extremal conformation are made relatively to the opposite one. More specifically, a variation in robustness correspond in our analyses to an increase or decrease in circumference (either globally or more specifically around the midshaft or the ends).

### 2.6 Analysis of allometry

To explore the assumption that the allometric patterns between columnar sauropods and non‐columnar sauropodomorphs are different, we tested the differences of allometric trajectory in our sample, taking columnarity as a group criterion. To do so, we performed for each analysis a Procrustes ANOVA (see Goodall, 1991), taking size and limb architecture (i.e. columnarity or not) into account. Each group is characterized by an allometric trajectory, notably defined by its slope and its intercept. We first tested the homogeneity of the slopes of the two groups (HoS test). A rejection of the assumption that the slopes are homogeneous would signify that the two allometric trajectories differ significantly (see Esquerré et al., 2017; Ferreira‐Cardoso et al., 2020). When the homogeneity of the slopes assumption was not rejected, we performed a permutation test for the similarity of the intercepts, using the function of Piras et al. (2011). The rejection of the assumption of equality of the intercepts would signify that the two allometric trajectories are not overlapping and are hence parallel (i.e. with similar slopes). Intercept tests are performed at the smallest value of centroid size in our sample rather than at the origin, as we do not have neonates in our sample (Ferreira‐Cardoso et al., 2020).

The Procrustes ANOVAs and the HoS tests were performed using the ‘procD.lm’ function in geomorphic version 3.3.1, with the permutation procedure provided by the RRPP version 0.6.1 package (Collyer & Adams, 2020). The significance of the homogeneity of slopes is tested with this function by stressing the significance of the interaction between size and group factors. For each test, 10,000 permutations were performed. To visualize the shape variation linked to the common allometry, that is that of the two groups together, we performed for the analyses where the HoS test did not reject the homogeneity of slopes, a pooled regression of shape coordinates centred on the group means (Klingenberg, 2016; Mitteroecker et al., 2004). The resulting common allometric component (CAC) and associated visualizations of allometry‐linked shape changes were obtained using the ‘cac’ and ‘showPC’ functions of the Morpho R package. The subsequently exported 3D models representing the negative and the positive extremal conformations (mapped on the meanshape) were compared by superimposition in Meshlab (see before in Section 2.5). For analyses, where the homogeneity of the slopes was rejected, we ran separately for each group a new GPA and visualized the size‐linked differences using the same functions.

## 3 RESULTS

### 3.1 Humerus

**FIGURE 2:**
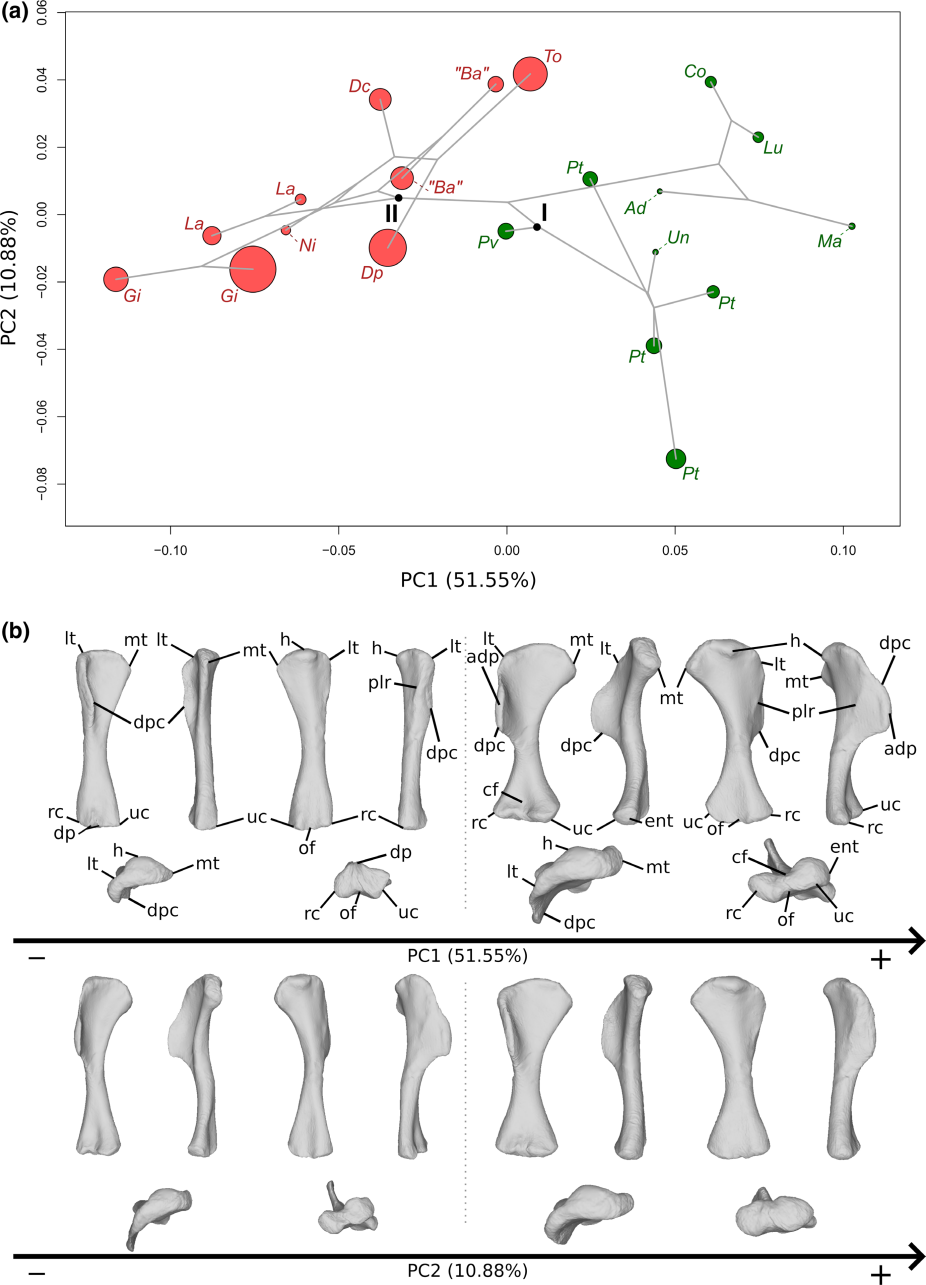
(a) Phylomorphospace of the humerus analysis along with the first two PCs. Green dots represent non‐sauropodiform sauropodomorphs, red dots represent sauropods. The diameter of the dots represents the centroid size of the specimen. Node I corresponds to the root of the tree and Node II to the estimation of the node Sauropoda. Taxonomic abbreviations: Ad: *Adeopapposaurus*; Ba: *Barosaurus africanus*; Co: *Coloradisaurus*; Dc: *Dicraeosaurus*; Dp: *Diplodocus*; Gi: *Giraffatitan*; La: *Lapparentosaurus*; Lu: *Lufengosaurus*; Ma: *Massospondylus*; Ni: *Nigersaurus*; Pt: *Plateosaurus*; Pv: *Plateosauravus*; To: *Tornieria*; Un: *Unaysaurus*. (b) Thin‐plate splines visualization of aligned theoretical shapes of the humerus analysis. The shape changes along with PC1 negative and positive shapes, and PC2 negative and positive shapes are observed. Each shape is represented, from left to right and top to down, in anterior, medial, posterior, lateral, proximal and distal views. In the proximal view, the top corresponds to the posterior side; in the distal view, the top corresponds to the anterior side. Anatomical abbreviations: Adp: Apex of deltopectoral crest; cf: cuboid fossa; dp: distal processes; dpc: deltopectoral crest; ent: entepycondyle; h: humeral head; lt: lateral tubercle; mt: medial tuberosity; of: olecranon fossa; plr: posterolateral ridge; rc: radial condyle; uc: ulnar condyle. PC, principal components

The first two axes of the PCA conducted on humeri express 62.43% of the total variation (Figure 2a). The first PC contributes 51.55% of the total variation and separates with few overlap the sauropods on the negative side from the non‐sauropodan sauropodomrophs on the positive side. More precisely, it tends to separate with some overlap on the negative extremity the specimens belonging to *Giraffatitan* from the other sauropods and on the positive extremity the Massospondylidae (i.e. *Massospondylus*, *Adeopapposaurus*, *Lufengosaurus* and *Coloradisaurus*) from the other non‐sauropodan sauropodomorphs. The shape of the negative extremity (Figure 2b) is proportionally less robust than the shape of the positive extremity, especially around the ends (Figure 2b). Its humeral head is less developed. Its medial tuberosity is considerably less developed and is pinched anteroposteriorly. The proximal end of the positive shape is bulkier, with a more developed humeral head, and a considerably more developed and posteriorly deflected medial tuberosity. A pronounced concavity marks the posterior limit between these two parts. The lateral tubercle is less developed in the negative shape than in the positive one but is sharply curved anteriorly, nearly forming a right‐angle corner in the proximal view. In the positive shape, the lateral tubercle is more gently curved. On the negative shape, the deltopectoral crest is considerably less developed, showing a slight sigmoid curvature in the anterior view. A variation of outline is discernible in the medial view: proximally to distally, the outline is successively concave then convex, with a proximal peak at the junction with the lateral tubercle and another more distal one, roughly in the middle of the proximal half. The deltopectoral crest of the positive shape is considerably developed, with a slight sigmoid curvature in the anterior view, and presents a bulge‐shaped apex, visible in anterior and lateral views. A posterolateral ridge, discernible on both shapes, is softly marked in the positive shape, whereas it is, in the negative shape, more marked, and is the location of convex strong curvature, complementary to the concave curvature seen in the anterior side of the bone. The shaft of the negative shape is straight, whereas it is straight in the positive one and sigmoid in medial and posterior views. Distally, the positive shape shows a deep cuboid fossa located anteriorly of the bone and a less‐developed olecranon fossa located posteriorly.

In the negative shape, the cuboid fossa is absent, whereas a distinctly marked olecranon fossa occurs on the posterior side of the bone. Two distal processes are present, roughly at the same location of the cuboid fossa. Two marked ridges are discernible on the medial side of the distal shaft, one being is laterally located. The distal end of the negative shape is barely wider than the midshaft mediolaterally, with an ulnar condyle slightly more developed than the radial condyle. The condyles are close and hardly discernible from each other. The medial margin of the radial condyle is circular, whereas the lateral margin of the ulnar condyle is more angled; the angles of the outline correspond to the ridges observed on the distal part of the shaft. The distal end of the positive shape is wider than the midshaft mediolaterally, with roughly equally developed and distinct condyles. They are relatively distant from each other. The radial condyle is roughly subovoid, whereas the ulnar condyle is more circular. This latter presents an entepicondyle, that is a mediolaterally oriented facet. A noticeable degree of torsion occurs between the proximal and the distal ends of the positive shape, whereas it is nearly non‐existent between the ends of the negative shape.

The second PC (Figure 2a) contributes 10.88% of the total variation. It tends to separate with some overlap on the negative side most of the Plateosauridae (i.e. *Plateosaurus* and *Unaysaurus*) and, to a lesser extent, the specimens of *Giraffatitan*, from a greater cluster formed by the other specimens. The shape of the negative extremity (Figure 2b) is globally proportionally less robust than that of the positive one (Figure 2b). The proximal end of the negative shape is less developed, with a noticeably less medially projected medial tuberosity, whereas the positive one is mediolaterally broad, with developed lateral tubercle, humeral head and medial tuberosity. On the negative shape, the deltopectoral crest is thin and developed; the regularity of the outline is disrupted by a prominent apex in the medial view. This apex is softly bulged and visible in the anterior view. The outline of the crest is nearly straight in the anterior view, and the anterior face of the bone is gently concave, without strong inflexion. The deltopectoral crest of the positive shape is thick and relatively homogeneously developed from its proximal beginning to its midshaft termination, in the medial view. A slight curvature of the outline in the anterior view, notably in the proximal part, gives a very softly sigmoidal aspect to the crest. It is materialized by a slight inflexion visible in the proximal view, with a concavity in the anterior side of the bone. The two shapes present a posterolateral ridge, but it is more marked in the positive shape, on a convexity complementary to the concavity of the anterior part of the bone. The shaft of the negative shape is slender and slightly sigmoid, whereas the shaft of the positive shape is straight and mediolaterally broad, especially medially, with an oval cross section. Distally, the negative shape shows relatively well‐marked cuboid and olecranon fossae, whereas, in the positive shape, only a slightly marked olecranon fossa is discernible. On the negative shape, the distal end is narrow mediolaterally, with a subovoid radial condyle and a subcircular ulnar condyle, presenting an entepicondyle. The distal end on the positive shape is broad mediolaterally, with condyles equally developed. They are located relatively close to each other, but are quite distinguishable, and are subovoid. A considerable degree of torsion exists between the ends of the negative shapes, whereas it is nearly non‐existent in the positive one.

### 3.2 Radius

**FIGURE 3:**
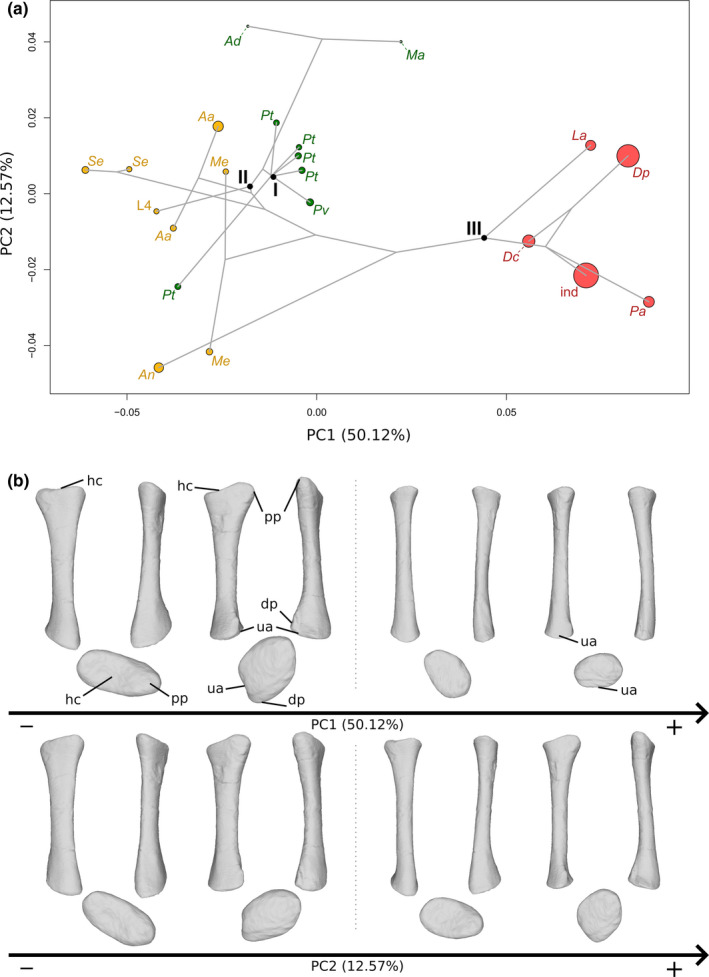
(a) Phylomorphospace of the radius analysis along with the first two PCs. Green dots represent the non‐sauropodiform sauropodomorphs, yellow dots represent non‐sauropodan sauropodiforms and red dots represent sauropods. The diameter of the dots represents the centroid size of the specimen. Node I corresponds to the root of the tree, Node II to the estimated node Sauropodiformes and Node III to the estimated node Sauropoda. Taxonomic abbreviations: Aa: *Aardonyx*; Ad: *Adeopapposaurus*; An: *Antetonitrus*; Dc: *Dicraeosaurus*; Dp: *Diplodocus*; ind: Indeterminate sauropod from Tendaguru; L4: MNHN.F.LES.400; La: *Lapparentosaurus*; Ma: *Massospondylus*; Me: *Melanorosaurus*; Pa: *Patagosaurus*; Pt: *Plateosaurus*; Pv: *Plateosauravus*; Se: *Sefapanosaurus*. (b) Thin‐plate splines visualization of aligned theoretical shapes of the radius analysis. The shape changes along with PC1 negative and positive shapes, and PC2 negative and positive shapes are observed. Each shape is represented, from left to right and top to down, in anterior, medial, posterior, lateral, proximal, and distal views. In proximal and distal views, the top corresponds to the anterior side. Anatomical abbreviations: Hc: Humeral cotyle; Dp: distal process; pp: proximal process; ua: ulnar articulation. PC, principal components

The first two axes of the PCA conducted on radii express 62.69% of the total variation (Figure 3a). The first PC contributes 50.12% of the total variation and clearly separates the non‐sauropodan sauropodomorphs from the sauropods. Among the non‐sauropodan sauropodomorphs, a separation with some overlap exists between the non‐sauropodiform sauropodomorphs and the non‐sauropodan sauropodiforms. The non‐sauropodan sauropodiforms group is more distant from the sauropod cluster than the non‐sauropodiform sauropodomorphs is. The shape of the negative extremity (Figure 3b) is proportionally considerably more robust than the shape of the positive extremity, especially around the ends (Figure 3b). Its proximal end is subovoid and elongated mediolaterally. Laterally, the end is slightly pinched and developed proximally, forming the proximal process. The lateral part is more developed than the medial part. It results that the proximal end is saddle‐shaped, with a central shallow depression, the radial humeral cotyle. On the positive shape, the proximal end is totally flat, subcircular and barely expanded anteroposteriorly. The anterior part is slightly more expanded than the posterior part. The expansion is more anteroposteriorly directed. Posteromedially, the margin of the end is slightly developed forming the articulation with the ulna. The shaft of the negative extremity is robust, straight and suboval in cross section, whereas it is, on the positive shape, slender, slightly curved anteroposteriorly notably on its distal half and subcircular in cross section. The distal end of the negative shape is oblique anteroposteriorly and its outline is subtriangular. A prominent distal process is observable, participating in a concave articulation of the ulna, posteriorly. On the positive shape, the distal end is only slightly oblique and is expanded mediolaterally. Posteriorly, a concavity articulating with the ulna is discernible in the distal view. A substantial difference in the degree of torsion exists between these two shapes. Compared to the shape of the negative extremity, the torsion of the ends of the positive one is different: approximately 45° anteriorly for the proximal end and approximately 45° medially for the distal end.

The second PC contributes 12.57% (Figure 3a) of the total variation and separates with strong overlaps, from the negative to the positive sides, the non‐sauropodan sauropodiforms, the sauropods and the non‐sauropodiform sauropodomorphs. More generally, the non‐sauropodan sauropodiforms are separated, with some overlap, from the non‐sauropodiform sauropodomorphs and the sauropods on the negative side of the axis. Within this group, *Antetonitrus* and the holotype of *Melanorosaurus* are more located on the negative side of the axis than the others. The shape of the negative extremity (Figure 3b) is proportionally considerably more robust than the shape of the positive extremity (Figure 3b). Its proximal end is more developed and suboval, whereas the proximal end of the positive shape is less oval and slightly elongated from the anteromedial direction to the posterolateral direction. Posterolaterally for both shapes, the posterolateral part of the end is slightly pinched and developed proximally, forming the proximal process. The posterolateral part is more developed than the anteromedial part. In both shapes, the proximal end is saddle‐shaped, with a central shallow radial humeral cotyle. The shaft of the negative shape is very bulky, straight and with an elliptical cross section, whereas the shaft of the positive one is very slender, sigmoid in anterior and posterior views and subcircular in cross section. The distal end of the two shapes is oblique. The negative one is bulky and expanded anteromedially to posterolaterally, with a subcircular outline only disrupted by the presence of the distal process pointing posteromedially, whereas the positive one is subsquared, with a distal process pointing posteriorly. The distal articulation with the ulna is in both shapes posteromedially oriented. There is nearly no degree of torsion between the ends of the negative shape, whereas some torsion exists for the positive one.

### 3.3 Ulna

**FIGURE 4:**
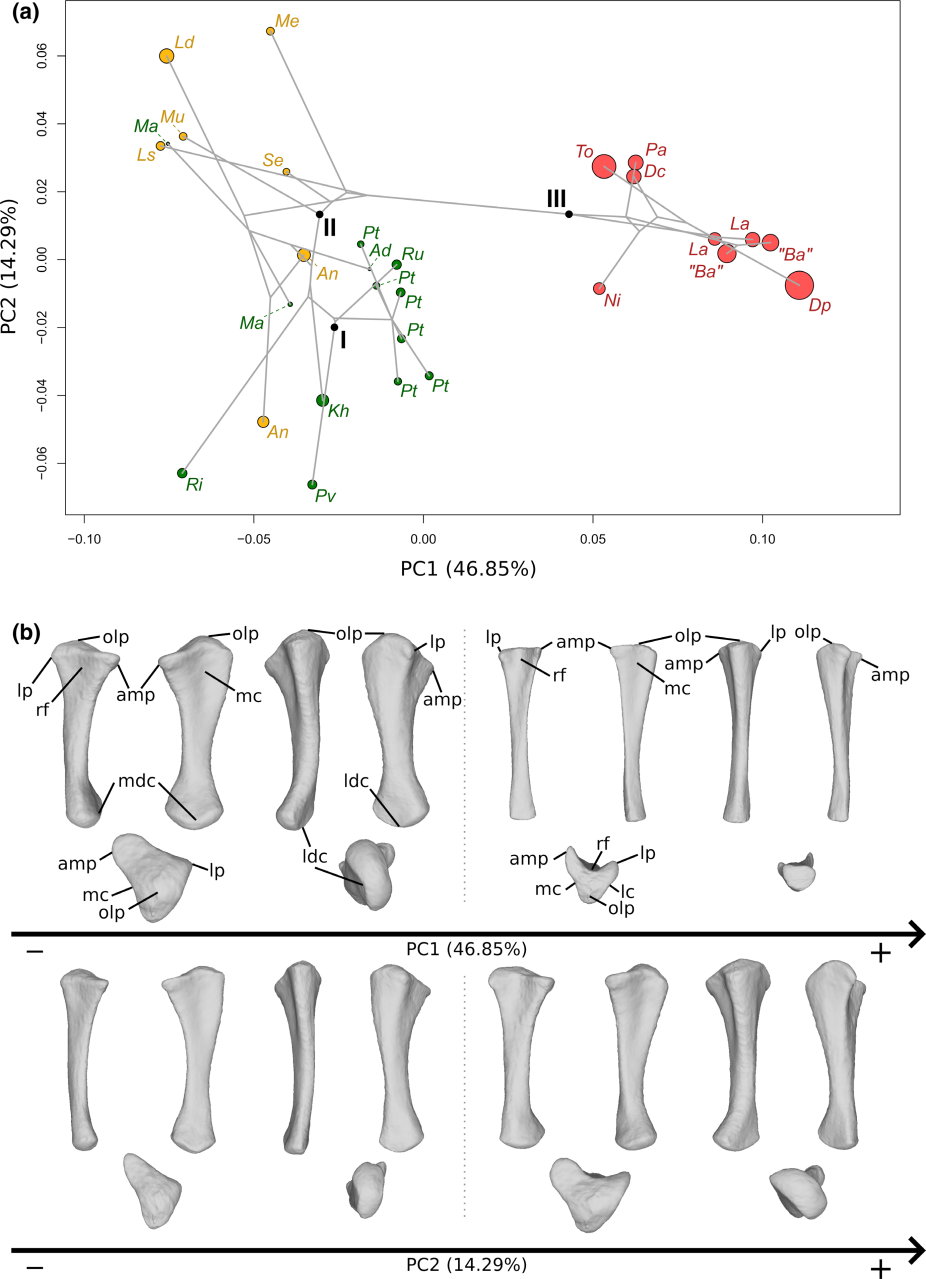
(a) Phylomorphospace of the ulna analysis along with the first two PCs. Green dots represent the non‐sauropodiform sauropodomorphs, yellow dots represent non‐sauropodan sauropodiforms and red dots represent sauropods. The diameter of the dots represents the centroid size of the specimen. Node I corresponds to the root of the tree, Node II to the estimated node Sauropodiformes and Node III to the estimated node Sauropoda. Taxonomic abbreviations: Ad: *Adeopapposaurus*; An: *Antetonitrus*; Ba: *Barosaurus africanus*; Dc: *Dicraeosaurus*; Dp: *Diplodocus*; Kh: *Kholumolumo*; La: *Lapparentosaurus*; Ls: *Lessemsaurus*; Ma: *Massospondylus*; Me: *Melanorosaurus*; Mu: *Mussaurus*; Ni: *Nigersaurus*; Ri: *Riojasaurus*; Ru: *Ruehleia*; Pa: *Patagosaurus*; Pt: *Plateosaurus*; Pv: *Plateosauravus*; Se: *Sefapanosaurus*; To: *Tornieria*. (b) Thin‐plate splines visualization of aligned theoretical shapes of the ulna analysis. The shape changes along with PC1 negative and positive shapes, and PC2 negative and positive shapes are observed. Each shape is represented, from left to right and top to down, in anterior, medial, posterior, lateral, proximal and distal views. In proximal and distal views, the top corresponds to the anterior side. Anatomical abbreviations: Amp: Anteromedial process; lc: lateral concavity; ldc: laterodistal concavity; lp: lateral process; mc: medial concavity; mdc: mediodistal concavity; olp: olecranon process; rf: radial fossa. PC, principal components

The first two axes of the PCA conducted on ulnae express 61.14% of the total variation (Figure 4a). The first PC contributes 46.85% of the total variation and clearly separates the non‐sauropodan sauropodomorphs on the negative side from the sauropods on the positive side. The non‐sauropodan sauropodiforms group is globally slightly more distant from the sauropod cluster than the non‐sauropodiform sauropodomorphs globally is. In the sauropod cluster, a slight separation occurs between a group formed by *Nigersaurus*, *Tornieria*, *Dicraeosaurus* and *Patagosaurus* on one side and *Diplodocus*, the two specimens belonging to *Lapparentosaurus* and the two specimens attributed to *B. africanus* on the other side. The shape of the negative extremity (Figure 4b) is proportionally considerably more robust than the shape of the positive extremity, especially around the ends (Figure 4b). The proximal end is subtriangular, radiating in three processes: the anteromedial process, the lateral process and the olecranon, posteriorly developed. This latter structure is also developed proximally, forming a domed process (the olecranon process), whereas it is markedly less developed in the positive shape, both posteriorly and proximally, so that the proximal surface of the end is nearly flat. The anteromedial process is, in the negative shape, thick and relatively rounded, and the lateral process is angled and less prominent. In the positive shape, the anteromedial process is developed but very thin, anteriorly curved, and the lateral process is similarly oriented and narrow but less developed. These two processes are well distinguishable from the rest of the proximal end. In the negative shape, the three processes of the proximal end, although developed, are not as clearly distinguishable as in the positive shape. The margin between the lateral process and the olecranon is relatively convex in proximal and lateral views, although a subtle concave depression marks the posterior border of the lateral process. The margins of the olecranon with the anteromedial process and the anteromedial process with the lateral process are concave in the proximal view. The former corresponds to a relatively consequent medial concavity, and the latter corresponds to the shallow fossa articulating with the radius, the radial fossa. In the positive shape, the margin between the olecranon and the anteromedial process is markedly concave, as is the margin between the olecranon and the lateral process, but to a substantially lesser extent. The latter margin is also convex in the lateral view. The margin between the anteromedial and lateral processes is strongly concave, forming a marked U, articulating with the radius. The radial fossa is hence considerably deep. The three concavities of the three margins and the development of the anteromedial and lateral processes give a clear triradiate shape to the proximal end. The shaft of the negative shape is proportionally considerably more robust than the positive one. It is curved medially, in anterior and posterior views, with three low ridges in its proximal half, corresponding to the development of the three processes of the proximal end. The posterior margin of the shaft is slightly sigmoid in medial and posterior views. Proximally, the shaft is also expanded anteroposteriorly, as well as laterally, to a lesser extent, complementary to the development of the proximal end. Distally, the shaft is expanded anteroposteriorly following the development of the distal end. In the positive shape, the shaft is almost straight, with some curvature associated with the decreasing flaring of the development of the ridges connected to the olecranon, posteriorly, and the anteromedial process, laterally. Three ridges are present in its proximal half, corresponding to the development of the three processes of the proximal end. The ridges below the anteromedial and lateral processes are acute, whereas the one below the olecranon is low. Distally, the shaft is extremely slender, and subtriangular in cross section, following the marked development of the ridges in its proximal part. Medially, a shallow proximal depression is discernible on both shapes, corresponding to the concavity observed on the margin between the olecranon and the anteromedial process but seems slightly deeper for the negative shape. The distal end of the negative shape is expanded anterolaterally to posteriorly. The anterolateral part is more developed than the posterior one. In medial and lateral views, the margins are convex. On the positive shape, the distal end is very poorly developed and is flat. It is subtriangular, with the three angles corresponding to the ridges on the shaft. A slight degree of torsion is discernible between the proximal and distal ends of the negative shape.

The second PC (Figure 4a) contributes 14.29% of the total variation and separates with strong overlaps, from the negative to the positive side, the non‐sauropodiform sauropodomorphs, the sauropods and the non‐sauropodan sauropodiforms. Most non‐sauropodiform sauropodomorphs are distributed on the negative side, whereas most of the non‐sauropodan sauropodiforms are distributed on the positive one. However, the two specimens of *Antetonitrus* plot with the non‐sauropodiform sauropodomorphs, and one of the two *Massospondylus* specimens plot with the non‐sauropodan sauropodiforms. Within the sauropods, a slight separation exists between a group formed by *Tornieria*, *Dicraeosaurus* and *Patagosaurus* and the other sauropods. The shape of the negative extremity (Figure 4b) is proportionally slightly less robust than the shape of the positive extremity, especially around the ends (Figure 4b). In the negative shape, the anteromedial process is narrow and triangularly developed, forming an acute angle in the proximal view. The lateral process is poorly developed. The olecranon is relatively developed in the proximal view, but relatively low elevated in the proximal view. In the positive shape, the anteromedial process is thick and very rounded in the proximal view. The lateral process is relatively less developed, as it is pinched in the proximal view, giving a sharp angle shape in this view, and forming a hook shape in anterior and lateral views. The olecranon is developed posteriorly and proximally, forming a domed process. On the negative shape, the margin between the anteromedial and the lateral processes is gently concave in the proximal view, so that the radial fossa is shallow. This also the case for the margin between the olecranon and the anteromedial process, with also has a shallow medial fossa. A subtle concave depression exists in the middle of the margin between the lateral process and the olecranon. The margin is, however, convex in lateral view. On the positive shape, the margin between the anteromedial process and the lateral process is concave in the proximal view, forming a concavity with a relatively deep radial fossa. The margin between the olecranon and the anteromedial process is concave in the proximal view, with a relatively marked fossa. The margin between the lateral process and the olecranon is relatively flat, although a subtle concave depression marks the posterior border of the lateral process. The relatively developed processes in the positive shape mark a relatively triradiate shape of the proximal end. This relatively triradiate shape is, however, less clear than in the shape of the negative extremity of the first PC, probably because of the substantially less marked concavities of the margins between each process, especially between the lateral process and the olecranon. The negative shape shows a different conformation: due to the gentle but clearly visible concavities, the olecranon and the anteromedial process are clearly distinguishable. This is not the case with the lateral process, given its poor development. The shape of the proximal end is, hence, subtriangular here. The shaft of the negative shape is proportionally relatively less robust and relatively less curved medially in the posterior view, whereas it is relatively more robust and curved in the positive one, but less expanded anteroposteriorly. On both shapes, there are three ridges in the proximal half, corresponding to the development of the three processes of the proximal end. In the negative one, the ridge below the anteromedial process is relatively acute, whereas the ridges are low below the olecranon and the lateral process. Conversely, in the positive shape, the ridges below the anteromedial and lateral processes are relatively acute, and the ridge below the olecranon is low. The posterior margin of the shaft of the negative shape is slightly sigmoid in the medial view, whereas it is only concave in the positive shape. A strong degree of torsion exists between the proximal and distal ends of the positive shape, whereas no substantial degree of torsion is discernible in the negative one. The distal end of the negative shape is anteroposteriorly expanded and oriented, whereas it is expanded laterally to anteriorly in the positive one. In the negative shape, the anterior part is developed and rounded, whereas the posterior part is less developed and more acute. In medial and lateral views, the margins are convex. In the positive shape, the anterior margin is poorly developed in the distal view, giving a half‐moon shape to the anterolateral part of the distal end. The posterior part is developed and suboval. In anteromedial and posterolateral views, the margins are convex.

### 3.4 Femur

**FIGURE 5:**
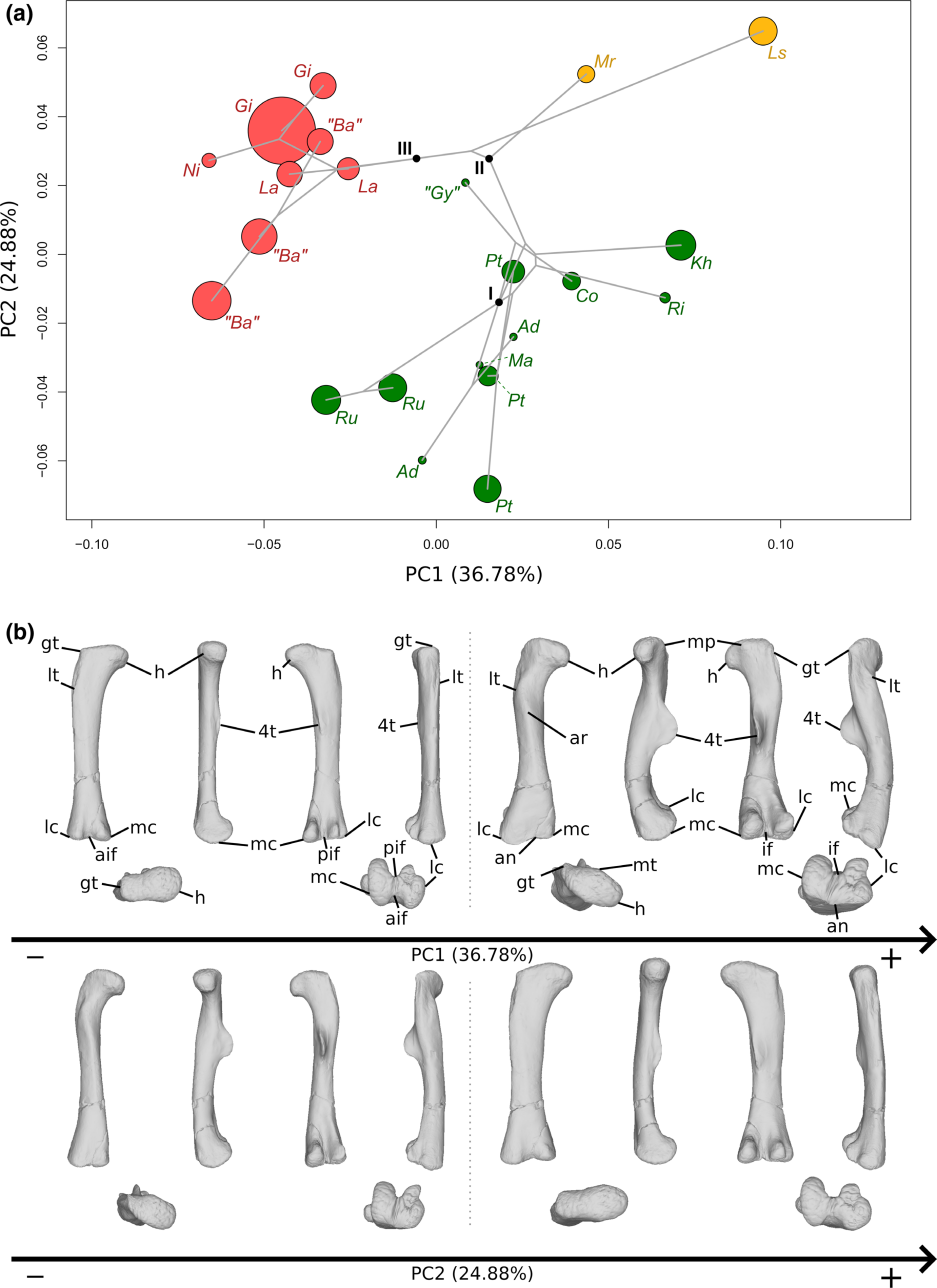
(a) Phylomorphospace of the femur analysis along with the first two PCs. Green dots represent the non‐sauropodiform sauropodomorphs, yellow dots represent non‐sauropodan sauropodiforms and red dots represent sauropods. Diameter of the dots represents the centroid size of the specimen. Node I corresponds to the root of the tree, Node II to the estimated node Sauropodiformes and Node III to the estimated node Sauropoda. Taxonomic abbreviations: Ad: *Adeopapposaurus*; Ba: *Barosaurus africanus*; Co: *Coloradisaurus*; Gi: *Giraffatitan*; Gy: *Gyposaurus sinensis*; Kh: *Kholumolumo*; La: *Lapparentosaurus*; Ls: *Lessemsaurus*; Ma: *Massospondylus*; Mr: *Meroktenos*; Ni: *Nigersaurus*; Ri: *Riojasaurus*; Ru: *Ruehleia*; Pt: *Plateosaurus*. (b) Thin‐plate splines visualization of aligned theoretical shapes of the femur analysis. The shape changes along with PC1 negative and positive shapes, and PC2 negative and positive shapes are observed. Each shape is represented, from left to right and top to down, in anterior, medial, posterior, lateral, proximal and distal views. In proximal and distal views, the top corresponds to the posterior side. Anatomical abbreviations: 4t: fourth trochanter; aif: anterior intercondylar fossa; an: anterior notch; ar: anterior ridge; gt: greater trochanter; h: Femoral head; lc: lateral condyle; lt: lesser trochanter; mc: medial condyle; pif: posterior intercondylar fossa. PC, principal components

The first two axes of the PCA conducted on the femora express 61.66% of the total variation (Figure 5a). The first PC contributes 36.78% of the total variation and separates with few overlap the sauropods on the negative side from the non‐sauropodan sauropodomorphs globally on the positive side. The shape of the negative extremity (Figure 5b) is proportionally slightly less robust than the shape of the positive extremity (Figure 5b), notably around the distal end. The femoral head is mediolaterally expanded in the negative shape, whereas it is expanded and oblique in the proximal view in the positive shape, departing from the anteromedial direction to the posterolateral one. The greater trochanter of the negative shape is a marked and developed lateral bump, not projecting on the shaft. On the lateral view, on the shaft, two ridges are discernible: a central one and an anterolateral one, the latter corresponding to the lesser trochanter. In the positive shape, the greater trochanter is discernible posterolaterally in the proximal view, as a marked bump, and laterally as a thick and low ridge projecting along the shaft. On the negative shape, the tip of the medial head, projected medially, is roughly at the same level as the lateral region of the greater trochanter. This medial part is well distinguishable in the proximal view, marked by an anterior and a posterior concavity in the proximal view. An anterior and a posterior development are discernible on this medial area in the proximal view. A shallow groove is discernible below the posterior concavity. On the positive shape, the femoral head is pointing slightly distally in the anterior view, so that its level is slightly below the level of the proximolateral margin of the greater trochanter. Anteriorly, the femoral head is convex. On the posterior margin, a developed central process, the medial tuber, is observable in proximal, medial and posterior views. The outline of the posterior margin is markedly concave between the greater trochanter and the medial tuber, and between the medial tuber and the tip of the femoral head. A marked depression below this latter concavity is discernible in medial and posterior views. The femoral shaft of the negative shape is fully straight in anteroposterior and mediolateral views, is mediolaterally expanded, and suboval in cross section, whereas it is, in the positive shape, sigmoid in lateral, anterolateral and medial views, and roughly subcircular in cross section. Right below the greater trochanter of the positive shape, anterolaterally located, a marked bump corresponds to the presence of a developed lesser trochanter. The two trochanters are separated by a marked groove. An anterior ridge, beginning below the femoral head, following the deflection of the shaft by contacting with the lesser trochanter, and terminating indistinctly on the distal half of the shaft, is discernible. Around the midshaft region, the negative shape shows a lowly developed ridge, the fourth trochanter. Its medial side, as well as the adjacent part of shaft, is marked by a shallow depression. In the positive shape, medially placed, the fourth trochanter is considerably developed. Its outline is regularly convex in the medial view, and sigmoid in the posterior view. A vast but shallow depression is noticeable on the medial face of the fourth trochanter and the adjacent shaft. On the negative shape, the distal end is developed antroposteriorly. Two condyles are discernible and well distinguishable because of deep posterior and anterior intercondylar fossae. On the positive shape, the distal end, and the portion of the shaft above it, are expanded mediolaterally and posteriorly. Two condyles are discernible and distinguishable because of a deep posterior intercondylar fossa and an anterior notch. They are less discernible than the shape of the negative extremity. The medial condyle of the two shapes is suboval, whereas the lateral condyle is particularly shaped: its lateral margin is markedly sigmoid in the negative shape, whereas is less marked in the positive one; the posterior part of this condyle is also markedly deviating laterally. In the negative shape, the lateral condyle is slightly less developed posteriorly and distally than the medial condyle, conversely to the positive one, where it is slightly more developed. The lateral condyle, in both shapes, projects on the posterior face of the shaft by an acute ridge. The medial condyle also projects on the posterior shaft, but by a less marked ridge. In the negative shape, the two condyles are also anteriorly projected by a relatively acute ridge. Two intercondylar fossae occur between these projections, anteriorly and posteriorly. On the positive shape, there is no projection of the condyles anteriorly. Hence, there is only one intercondylar fossa, posteriorly located. There is a noticeable degree of torsion existing between the ends of the positive shape, absent in the negative shape.

The second PC contributes 24.88% (Figure 5a) of the total variation and separates the two non‐sauropodan sauropodiforms on the positive extremity from the other taxa. Most of the sauropods are in the positive side of the axis and are less distant from the positive extremity than the non‐sauropodiform sauropodomorphs, which are more located and dispersed around the origin of the axis. The shape of the negative extremity (Figure 5b) is proportionally markedly more slender than the shape of the positive extremity (Figure 5b), which is more robust notably around the ends. Its femoral head is expanded and oblique in the proximal view, departing from the anteromedial direction to the posterolateral one, whereas the positive one is expanded mediolaterally. In the proximal view, the greater trochanter of the negative shape is discernible posterolaterally as a marked process, and in lateral view as a thick and low ridge projecting on the shaft. The greater trochanter of the positive shape is a marked and developed anteroposteriorly oblique lateral bump, not projecting on the shaft. On lateral view, two ridges are discernible: a central one and an anterolateral one, the latter being the lesser trochanter. The femoral head of the negative shape is anteriorly convex. On the posterior margin, the medial tuber is observable in proximal, medial and posterior views. The outline of the posterior margin is markedly concave between the greater trochanter and the medial tuber, and between the medial tuber and the tip of the femoral head. A marked depression below these two concavities is discernible in medial and posterior views. In the femoral head of the positive shape, the medial tip, projected medially, is more elevated than the lateral region of the greater trochanter. This medial part is well distinguishable in the proximal view, marked by an anterior and a posterior concavity in the proximal view. An anterior and a posterior development are discernible on this medial area in the proximal view. A shallow groove is discernible below the posterior concavity. The femoral shaft of the negative shape is almost straight in lateral and medial views, but sigmoid in anterolateral view, and roughly circular in cross section, whereas the positive one is fully straight in anterior and posterior views, and slightly curved posteriorly, as seen in medial and lateral views. It is also considerably mediolaterally expanded, and elliptical in cross section. In the negative shape, the developed lesser trochanter is anterolaterally located, right below the greater trochanter, materialized by a marked bump. The two trochanters are separated by a depression. An anterior ridge, beginning below the femoral head, following the deflection of the shaft by contacting with the lesser trochanter, and terminating indistinctly on the distal half of the shaft, is discernible. Above the midshaft, medially placed, the fourth trochanter is substantially developed. Its outline is regularly convex in the medial view, and sigmoid in the posterior view. A large but shallow depression is noticeable on the medial face of the fourth trochanter and the adjacent shaft. In the positive shape, the fourth trochanter is a slightly developed ridge. Its medial side, as well as the adjacent part of the shaft, is marked by a shallow depression. In the negative shape, the distal end, and the portion of shaft above it, is expanded posteriorly, whereas the distal end of the positive shape is only developed anteroposteriorly. Two condyles are discernible and distinguishable in both shapes, but differently: by the occurrence of a deep posterior intercondylar fossa and of an anterior notch in the negative shape, that of deep posterior and anterior intercondylar fossae in the positive one. They are less discernible than for the model of the negative extremity of the first PC. The medial condyle is suboval in both shapes, whereas the lateral condyle shows a posterior part markedly deviating laterally in the negative shape and a sigmoid lateral margin in the positive one. In the negative shape, the lateral condyle is slightly more developed posteriorly than the medial condyle; in the positive shape, the lateral condyle is slightly more developed laterally and distally than the medial condyle. In both shapes, the lateral condyle projects on the posterior face of the shaft by an acute ridge. The medial condyle also projects on the posterior shaft, but by a less marked ridge. In the positive shape, the two condyles are also anteriorly projecting by a very low bump. The intercondylar fossae are well marked along with the anterior and/or posterior projections in both shapes. A noticeable torsion exists between the two ends of the negative shape, absent between the two ends of the positive one.

### 3.5 Tibia

**FIGURE 6:**
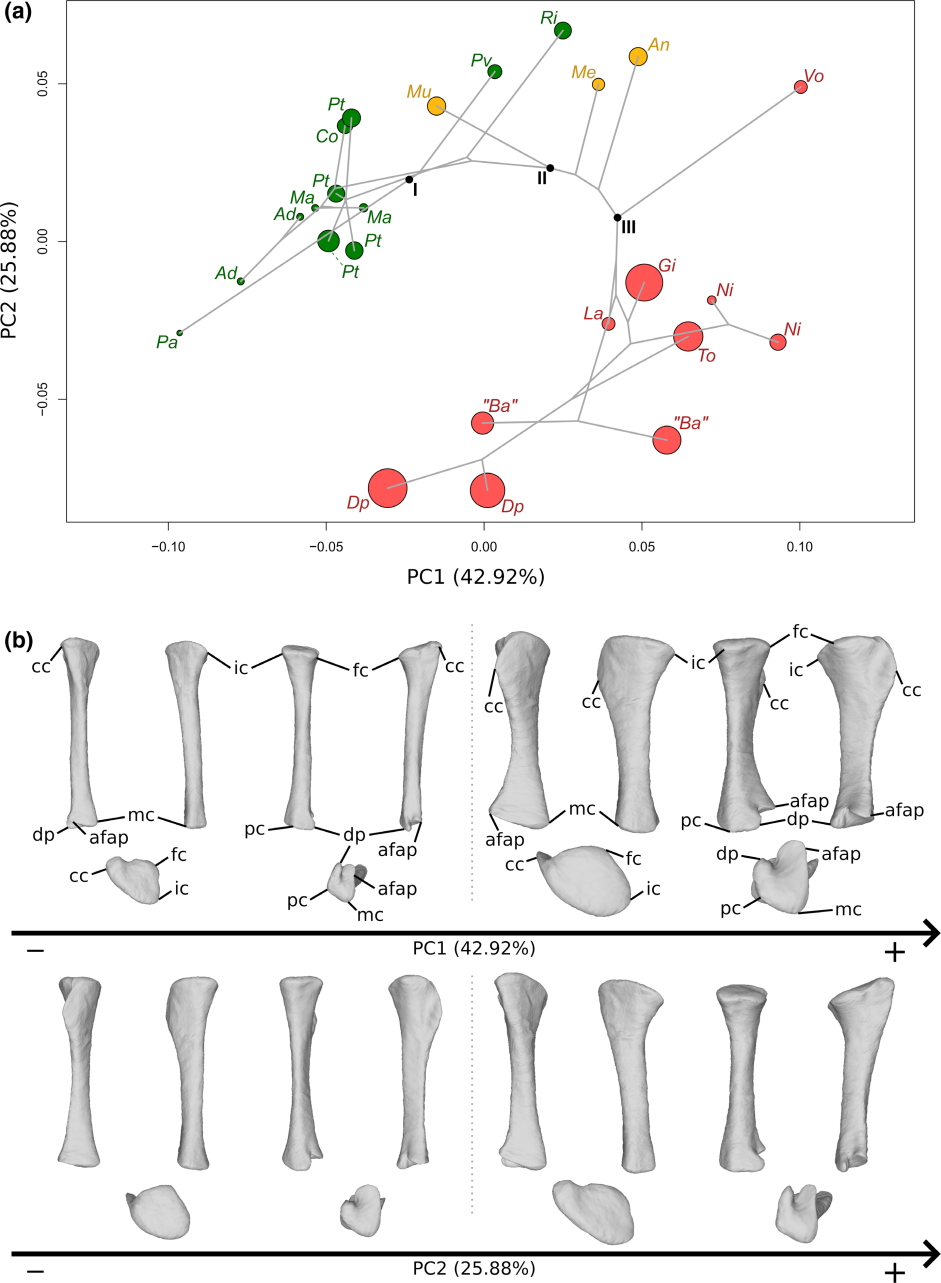
(a) Phylomorphospace of the tibia analysis along with the first two PCs. Green dots represent the non‐sauropodiform sauropodomorphs, yellow dots represent non‐sauropodan sauropodiforms and red dots represent sauropods. The diameter of the dots represents the centroid size of the specimen. The diameter of the dots represents the centroid size of the specimen. Node I corresponds to the root of the tree, Node II to the estimated node Sauropodiformes and Node III to the estimated node Sauropoda. Taxonomic abbreviations: Ad: *Adeopapposaurus*; An: *Antetonitrus*; Ba: *Barosaurus africanus*; Co: *Coloradisaurus*; Dp: *Diplodocus*; Gi: *Giraffatitan*; La: *Lapparentosaurus*; Ls: *Lessemsaurus*; Ma: *Massospondylus*; Me: *Melanorosaurus*; Mu: *Mussaurus*; Ni: *Nigersaurus*; Ri: *Riojasaurus*; Pa: *Panphagia*; Pt: *Plateosaurus*; Pv: *Plateosauravus*; To: *Tornieria*; Vo: *Volkheimeria*. (b) Thin‐plate splines visualization of aligned theoretical shapes of the tibia analysis. The shape changes along with PC1 negative and positive shapes, and PC2 negative and positive shapes are observed. Each shape is represented, from left to right and top to down, in anterior, medial, posterior, lateral, proximal and distal views. In proximal and distal views, the top corresponds to the lateral side. Anatomical abbreviations: Afap: Ascending process articular facet; cc: cnemial crest; dp: descending process; fc: fibular condyle; ic: internal condyle; mc: medial corner of the distal end; PC: posterior corner of the distal end. PC, principal components

The first two axes of the PCA conducted on tibiae express 68.80% of the total variation (Figure 6a). The general structure of the data, taking the phylogenetic relationships into account, follows a curve. The different groups are well discriminated only when taking the information of the two axes simultaneously into account. The first PC contributes 42.92% of the total variation. The non‐sauropodan sauropodomorphs and the sauropods are separated with considerable overlap along the axis, the latter group being more located on the positive side of the axis than the former one. The overlapping group is composed of all the non‐sauropodan sauropodiforms, *Plateosauravus*, *Riojasaurus*, one of the two specimens attributed to *B. africanus* and the two specimens of *Diplodocus*. The shape of the negative extremity (Figure 6b) is proportionally considerably more slender than the shape of the positive extremity (Figure 6b). The proximal end is developed in both shapes, but its surface is markedly oblique in lateral view in the negative shape. Three noticeable areas are discernible: the cnemial crest in the anterolateral part, the fibular condyle (or lateral condyle) in the posterolateral part, and the internal condyle, in the posteromedial part. The cnemial crest of the negative shape is particularly developed and constitutes the highest point of the end. It is projecting anterolaterally and is slightly deviating laterally. The cnemial crest comprises two small bumps corresponding to its two apexes: one pointing anterolaterally and one pointing posterolaterally. A ridge starts at this latter point and follows the medial margin, to terminate in the posterior part of the proximal end, merging with the medial margin. It delimits with the medial ridge a pronounced development forming an anteromedial bulge. In the positive shape, the cnemial crest is considerably thin. It is not properly developed at the same level as the fibular and the internal condyles, but more distally, along the shaft. A connection between the apex point of the cnemial crest and the proximal end, however, exists. It corresponds to a thin ridge, descending along the shaft. The base of this connection is slightly wider than the mediolateral width of the cnemial crest. The most anterolateral point of the cnemial crest is located globally at the middle of the crest in lateral view. The cnemial crest is projecting anterolaterally. The surface of the proximal end is almost flat. The fibular condyle of the negative extremity is very developed posterolaterally, as well as in its long axis. A small acute convexity, articulating with the fibula, is pointing anterolaterally, on the anterior margin of the fibular condyle. The fibular condyle of the positive shape is well developed but not clearly delimited in the proximal view. It is, however, marked by the existence of a deep fossa located on the anterior part, separating it from the cnemial crest, as seen in lateral view. This fossa and the anterior part of the fibular condyle correspond to the proximal area articulating with the fibula. The internal condyle of the negative shape is developed posteromedially, whereas it is more developed posteriorly in the positive one; it is not clearly distinguishable, despite the slight depression existing between it and the fibular condyle. The medial margin of the proximal end is convex in the proximal view in both shapes. In the negative one, the cnemial crest, fibular and internal condyles are well distinguishable. This is notably because of the marked concavities existing between the cnemial crest and the fibular condyle, and between the fibular and internal condyles. It roughly gives the negative shape only a T‐shape to the proximal end in the proximal view. In the positive shape, excepting the cnemial crest, the proximal end is suboval in the proximal view. The shaft of the negative shape is very slender and only expanded at the base of the proximal end. It is subcircular to subsquare in cross section. In the positive shape, the shaft is thick, expanded anteroposteriorly, and oval in cross section. In the negative shape, the concavity between the cnemial crest and the fibular condyle is relatively marked along the shaft, forming a fossa where part of the proximal fibula articulates. The cnemial crest is hence clearly distinguishable from the shaft in lateral view, contrarily to the medial view, where it is indistinguishable. In the positive shape, because of the deep anterolateral fossa, the cnemial crest is clearly distinguishable from the shaft in lateral view, contrarily to the medial view, where it is indistinguishable. The distal end of the negative shape is only slightly more developed compared to the shaft cross section, whereas it is, in the positive one, subtantially developed mediolaterally. In both shapes, it is composed of two parts, the articular facet for the ascending process of the astragalus, and the descending process (or posertoventral/posterolateral process), both laterally oriented. They are separated by a groove. In the negative shape, the articular facet of the ascending process is wider than the descending process, but they are equally developed laterally. In the positive shape in the distal view, the articular facet of the ascending process is wide and developed laterally, whereas the descending process is thin and poorly developed laterally. In both shapes, the descending process is more developed distally than the articular facet of the ascending process. Both distal ends comprise also a medial and a posterior corner. The medial corner is more developed than the posterior corner, especially in the positive shape. In the negative shape, the medial corner is well distinguishable as it is bordered by two concavities existing between this corner with the ascending process and between this corner and the posterior corner. In the positive shape, the medial corner is less distinguishable, as it is bordered by a subtly concave anteromedial margin and a convex posteromedial margin. In both shapes, some ridges departing from the ends are discernible along the shaft.

The second PC contributes 25.88% of the total variation (Figure 6a). No clear clustering appears on this axis alone. Within the non‐sauropodan sauropodomorphs, all the non‐sauropodan sauropodiforms are located on the positive extremity of the axis, with the non‐sauropodiform sauropodomorphs *Plateosauravus* and *Riojasaurus*, whereas the rest of the non‐sauropodiform sauropodomorphs are more located around the origin of the axis, on the positive side, with the exception of *Panphagia* and one of the two specimens of *Adeopapposaurus*. The sauropod group is divided into three subgroups: the specimens of *Diplodocus* and *B. africanus* on the negative extremity of the axis, *Volkheimeria* on the positive one and all the other ones around the origin. The shape of the negative extremity (Figure 6b) is proportionally less robust than the shape of the positive extremity (Figure 6b). The proximal end is developed in both shapes. Its surface is straight anteroposteriorly in the negative shape, whereas it is markedly oblique in the positive one. The cnemial crest of the negative shape is considerably thin. It is not properly developed at the same level as the fibular and internal condyles, but more distally, along the shaft. A very tenuous connection exists between the apex point of the cnemial crest and the proximal end. It corresponds to a very thin ridge, descending along the shaft. The base of this connection is as wide as the width of the cnemial crest. The most anterolateral point of the cnemial crest is located globally at the middle of the crest in the lateral view. The cnemial crest is projecting anterolaterally. The surface of the proximal end is almost flat, but the proximal surface of the fibular condyle is slightly more elevated. The cnemial crest of the positive shape is particularly developed and constitutes the highest point of the proximal end. It is projecting anterolaterally. The cnemial crest comprises two small bumps corresponding to its two apexes: one pointing anterolaterally and one pointing posterolaterally. A ridge starts at this latter point and follows the medial margin, to terminate in the posterior part of the proximal end, merging with the medial margin. It delimits with the medial ridge a pronounced development forming an anteromedial bulge. The fibular condyle of the negative shape is well developed laterally but not clearly distinguishable in the proximal view. On its anterolateral margin, a slight convexity disrupts the straight outline of the condyle. A deep fossa exists on the shaft below this area, as seen in lateral view. This fossa articulates with the fibula. The fibular condyle of the positive shape is relatively developed posterolaterally, but also in its long axis. A small convexity articulating with the fibula is pointing anterolaterally, on the anterior margin of the fibular condyle. The internal condyle of the positive shape is developed posteromedially, whereas it is not clearly distinguishable and posteriorly developed in the negative one. In both shapes, the medial margin of the proximal end is slightly convex in the proximal view. In the negative shape, excepting the cnemial crest, the proximal end is subtriangular in the proximal view. In the positive shape, the cnemial crest and the fibular and internal condyles are relatively well distinguishable. The fibular condyle is marked by two concavities on its anterior and posterior margins. They are, however, less marked than in the shape of the negative extremity of the first axis. The anterior concavity is marked and visible in proximal and lateral views. The posterior concavity is, however, only slightly marked in the proximal view and is better visualized in the lateral view. In the proximal view, the proximal end is very roughly T‐shaped. The shaft of the negative extremity is slender, expanded anteroposteriorly and oval in cross section, whereas it is, in the positive one, proportionally more robust and relatively circular in cross section. In both shapes, the cnemial crest is clearly distinguishable from the shaft in the lateral view, contrarily to the medial view, where it is indistinguishable. In the positive shape, the concavity between the cnemial crest and the fibular condyle is relatively marked along with the shaft, forming a fossa where the fibula articulates; the shaft is expanded anteroposteriorly at the base of the proximal end and is expanded mediolaterally at the base of the distal end. The distal end is developed mediolaterally in both shapes, especially in the negative one, compared to the shaft cross section. The articular facet of the ascending process of the astragalus and the descending process is both laterally oriented and separated by a groove. In the negative shape, in the distal view, the articular facet of the ascending process is wide, and the descending process is thin and poorly developed. In the positive shape, the articular facet of the ascending process is wider than the descending process, but it is only slightly more developed laterally. In both shapes, the descending process is more developed distally than the articular facet of the ascending process, and the medial corner is more developed than the posterior corner. In the negative shape, the posterior corner is reduced to a regular convexity, and its outline is barely indistinguishable. The medial corner of the positive shape is well distinguishable as it is bordered by two concavities existing between this corner and the articular facet of the ascending process and between this medial corner and the posterior corner. In the negative shape, the medial corner is less distinguishable as it is bordered by two convex margins. In both shapes, some ridges departing from the ends are discernible along the shaft.

### 3.6 Fibula

**FIGURE 7:**
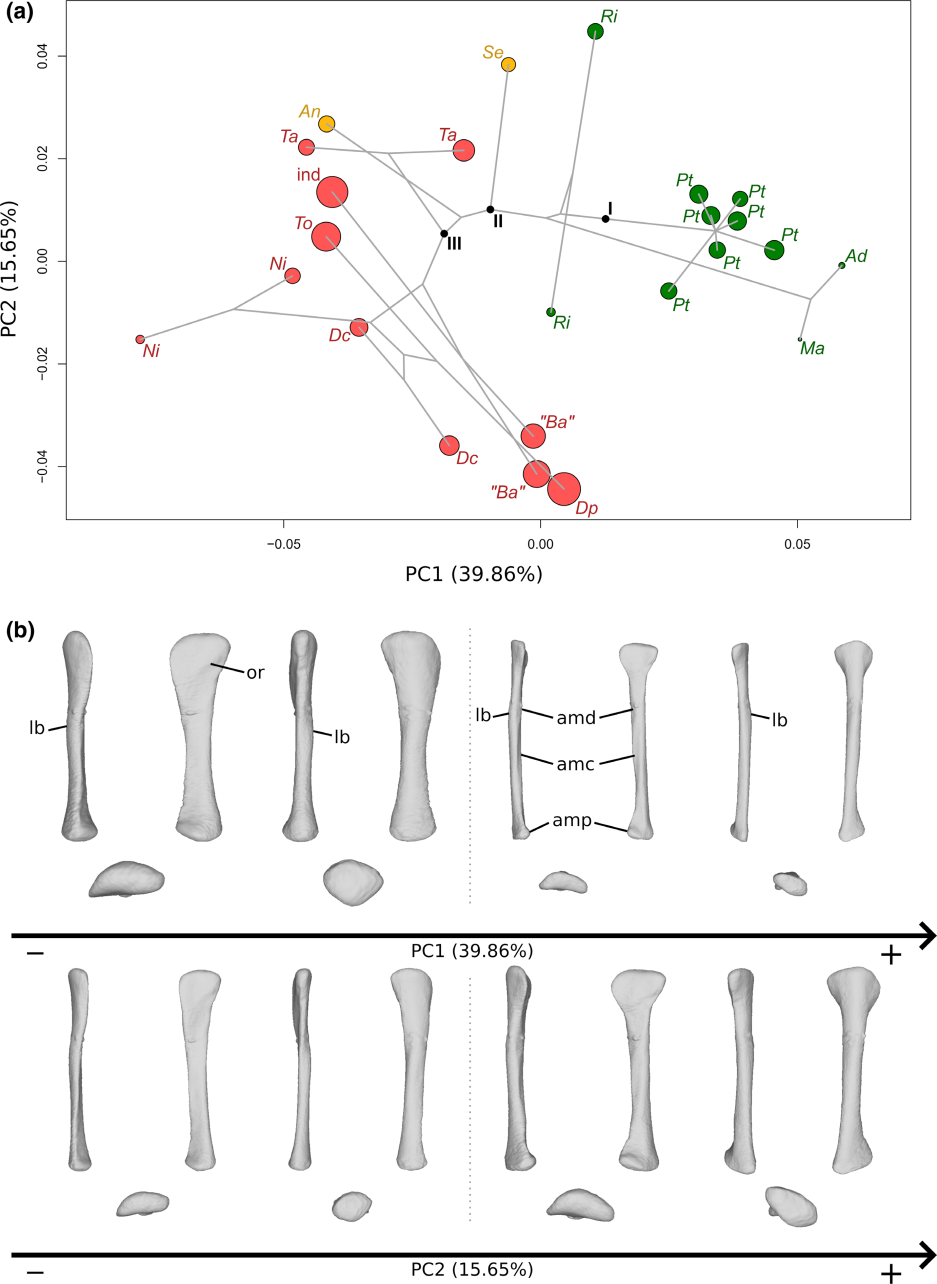
(a) Phylomorphospace of the fibula analysis along with the first two PCs. Green dots represent the non‐sauropodiform sauropodomorphs, yellow dots represent non‐sauropodan sauropodiforms and red dots represent sauropods. The diameter of the dots represents the centroid size of the specimen. Node I corresponds to the root of the tree, Node II to the estimated node Sauropodiformes and Node III to the estimated node Sauropoda. Taxonomic abbreviations: Ad: *Adeopapposaurus*; An: *Antetonitrus*; Ba: *Barosaurus africanus*; Dc: *Dicraeosaurus*; Dp: *Diplodocus*; Gi: *Giraffatitan*; ind: indeterminate sauropod from Tendaguru; La: *Lapparentosaurus*; Ma: *Massospondylus*; Ni: *Nigersaurus*; Pt: *Plateosaurus*; Ri: *Riojasaurus*; Se: *Sefapanosaurus* Ta: *Tazoudasaurus*; To; *Tornieria*. (b) Thin‐plate splines visualization of aligned theoretical shapes of the fibula analysis. The shape changes along with PC1 negative and positive shapes, and PC2 negative and positive shapes are observed. Each shape is represented, from left to right and top to down, in anterior, medial, posterior, lateral, proximal and distal views. In proximal and distal views, the top corresponds to the lateral side. Anatomical abbreviations: amc: anteromedial concavity; anteromedial depression; amp: amteromedial process; lb: lateral bulge; or: oblique ridge. PC, principal components

The first two axes of the PCA conducted on fibulae express 55.51% of the total variation (Figure 7a). The general structure of the data, taking the phylogenetic relationships into account, follows a curve. The different groups are well discriminated only by taking the information of the two axes simultaneously into account. The first PC contributes 39.86% of the total variation. The sauropods and the two sauropodiforms are overlapping on the negative side of the axis. The non‐sauropodiform sauropodomorphs are relatively well separated from the other specimens on the positive side and around the origin of the axis. The shape of the negative extremity (Figure 7b) is proportionally considerably more robust than that of the positive extremity (Figure 7b). The proximal end of the negative shape is globally anteroposteriorly developed, but the anterior part is deviating anteromedially, and the posterior tip is pointing posterolaterally. The lateral margin of the end is convex in the proximal view, whereas the lateral margin is anteriorly concave and posteriorly convex. The proximal end of the positive shape is anteroposteriorly expanded and slightly incurved medially, forming a crescent shape in the proximal view. The medial margin of the end is indeed concave, whereas the lateral one is convex. In the negative shape, the anterior part of the end is pinched mediolaterally. In lateral view, the outline of the proximal end is very softly convex on its posterior part and is markedly convex on its anterior half. The anterior part of the proximal end of the positive shape is slightly pinched mediolaterally, and the posterior part is rounded. In the lateral view, the proximal end is regularly convex. In the negative shape, the slope of the anterior junction of the proximal end with the shaft is relatively soft, the junction is not clearly discernible from the shaft. No depression is discernible in this area. The slope of the posterior junction is steeper, the junction is a relatively thick crest discernible from the shaft. The joining point with the shaft is not discernible in the lateral view. A very small depression is discernible in the posterior view at the junction point between the proximal end and the shaft. In the medial view, the surface articulating with the tibia is marked by a markedly oblique ridge. The surface is subtriangular. In the positive shape, the anterior and posterior projections of the proximal end are joining the shaft with a relatively steep slope. The junctions are thin ridges, located medially in anterior and posterior views, their joining point with the shaft are not discernible in the lateral view. Two subsequent depressions are discernible in these two areas. In the medial view, the surface articulating with the tibia is marked by a slightly oblique ridge. An anterocentral soft bump is discernible along the medial side of the proximal end. The shaft of the negative shape is straight and suboval in cross section, since it is anteroposteriorly expanded, whereas in the positive one, it is relatively straight, with a slight medial curvature and a subcircular cross section. In the positive shape only, a slight depression is observed on the anteromedial side of the proximal half. In both shapes, a slight degree of torsion on the shaft is discernible, materialized slightly above the midshaft by a lateral bump, so that the outline of the midshaft region is sigmoid in the posterior view. A depression is discernible in the negative shape, posterior to that region. In the negative shape, the anterior side of the shaft is an acute ridge, whereas the posterior margin is thick and circular. A large part of the anteromedial part of the shaft right above the distal end is slightly concave. In the positive shape, the anteromedial part of the shaft is slightly concave on its distal half. This concavity is bordered by an anterior and a medial ridge. A last anteromedial concavity is discernible at the distalmost part of the shaft, bordering the distal end. The posteromedial part of the shaft is flat. In the negative shape, the distal end is roughly square in the distal view and convex in the lateral view. The lateral margin is convex in the distal view. The anteriormost point of the distal end is linked to the shaft by a ridge. The medial part is developed but not developing a process pointing proximally. In the positive shape, the distal end is expanded and oblique anteromedially to posterolaterally. It is suboval in the distal view. An anterior part, more developed, is slightly discernible from a posterior part because of a slight medial concavity. The anterior part of the distal end is linked to the shaft by an acute ridge. In the medial view, an anteromedial process is discernible, developed anteromedially and pointing proximally. In the positive shape, a slight degree of torsion occurs between the proximal and distal ends, whereas it is nearly not discernible between the proximal and distal ends of the negative shape.

The second PC contributes 15.65% of the total variation (Figure 7a). The two specimens attributed to *B. africanus*, *Diplodocus* and one of the two specimens attributed to *Dicraeosaurus* are separated on the negative extremity from the other specimens, located from around the origin of the axis to the positive extremity. The two non‐sauropodan sauropodiforms and the holotype of *Riojasaurus* are the only non‐sauropodan sauropodomorphs located on the positive extremity, whereas the two specimens of *Tazoudasaurus* are the sauropods the closest to the positive extremity. The shape of the negative extremity (Figure 7b) is roughly as robust as the shape of the positive extremity (Figure 7b) but slightly less expanded around the ends. In the negative shape, the proximal end is globally anteroposteriorly developed, but the anterior part is deviating anteromedially, and the posterior tip is pointing posterolaterally. The lateral margin of the end is convex in the proximal view, it is anteriorly slightly concave and posteriorly slightly convex. The anterior part of the end is slightly pinched mediolaterally. The proximal end of the positive shape is slightly roughly crescent shaped, with a softly concave medial margin and a markedly convex lateral margin. The anterior part is deviated anteromedially and pinched anteroposteriorly. The posterior part is rounded. In the lateral view, the proximal end of the negative shape is very softly convex on its posterior part and is markedly convex on its anterior half, whereas it is, on the positive one, relatively regularly convex. In the negative shape, the slope of the anterior junction of the proximal end with the shaft is relatively soft, the junction is not clearly discernible from the shaft. A slight depression is discernible in this area. The slope of the posterior junction is steeper, the junction is a relatively thick crest discernible from the shaft. The anterior and posterior junction points with the shaft are not discernible in the lateral view. A small depression is observable in the posterior view at the junction point between the proximal end and the shaft. In the medial view, the surface articulating with the tibia is marked by a markedly oblique and curved ridge. The surface is subtriangular to crescent‐like shaped. In the positive shape, the anterior and posterior projections of the proximal end are joining the shaft with a relatively steep slope. The junctions are thin ridges, located medially in anterior and posterior views; their joining points with the shaft are not discernible in the lateral view. Two subsequent soft depressions are discernible in these two areas. In the medial view, the surface articulating with the tibia is marked by a slightly oblique ridge. An anterocentral proximodistal bump is discernible along with the medial side of the proximal end. The shaft of the negative shape is straight and suboval in cross section, since it is anteroposteriorly expanded, whereas it is, in the positive shape, relatively straight, with a slight medial curvature and subcircular cross section. In the positive shape only, depression is observed on the anteromedial side of the proximal half. In both shapes, a slight degree of torsion on the shaft is discernible, materialized slightly above the midshaft by a lateral bump, so that the midshaft outline is sigmoid in the posterior view. In the negative shape, the lateral bump is more marked and the sigmoidicity is slighter than in the positive shape. Moreover, a marked depression is discernible in the negative shape, posterior to that region. In the negative shape, the anterior side of the shaft is an acute ridge, whereas the posterior margin is thick and circular. A large section of the anteromedial part of the shaft right above the distal end is concave. In the positive shape, the anteromedial part of the shaft is slightly concave on its distal half. This concavity is bordered by an anterior and a medial ridge. A last anteromedial concavity is discernible at the distalmost part of the shaft, bordering the distal end. The posterior part of the shaft is relatively rounded. In the negative shape, the distal end is subsquare in the distal view and slightly convex in the lateral view. The lateral margin is convex in the distal view. In both shapes, the anterior part of the distal end is linked to the shaft by an acute ridge. The distal end of the positive shape is expanded and oblique anteromedially to posterolaterally and is suboval in the distal view. The anterior half is more developed than the posterior half. In the medial view, an anteromedial process is discernible, in the positive shape only, substantially developed anteromedially and pointing proximally, whereas no anteromedial process pointing proximally is discernible in the negative one. A slight degree of torsion is discernible between the proximal and distal ends. In the negative one, the distal end is anterolaterally directed in the distal view, whereas the positive one is anteromedially directed.

### 3.7 Allometry

Based on Procrustes ANOVA results (see Table S3), we found a significant effect of size (*p* < 0.05) on shape variation in the humerus, the radius, the ulna and the tibia. A *p*‐value between 0.1 and 0.05 was found for the femoral (*p* = 0.054) and the fibular (*p* = 0.0806) shape variation. Also, we found a significant delimitation between the non‐columnar sauropodomorphs and the columnar sauropods for the six bones investigated (*p* < 0.0001). The homogeneity of slopes test failed to reject the hypothesis of parallel slopes (i.e. a common allometric trend between the two groups hypothesis) for the humerus, radius, ulna and femur (*p* > 0.05). The homogeneity was, however, rejected for the tibia and the fibula (*p* < 0.05). The intercept test, performed when homogeneity was not rejected, failed to reject the null hypothesis (same intercept for the two allometric groups) for the humerus, the radius and the ulna. The null hypothesis was, however, rejected for the femur.

**FIGURE 8:**
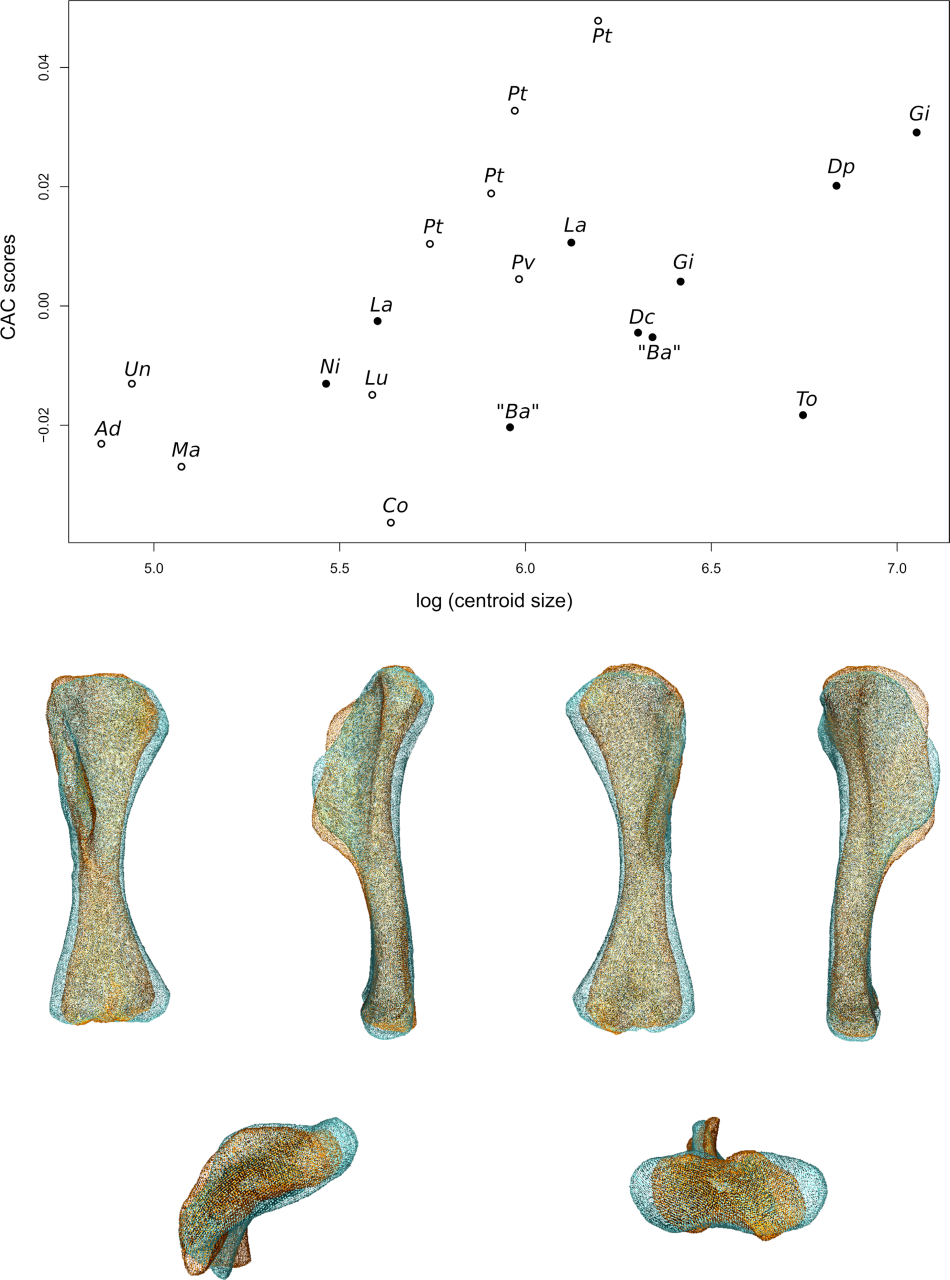
Allometric pattern of the humerus: Top: bivariate plot showing the CAC scores against the natural logarithm of the centroid size. Open circles represent the non‐columnar sauropodomorphs, filled circles the columnar sauropods. Bottom: Thin‐plate splines visualizations of shape changes along with the CAC. Shape changes at minimal size are displayed in cyan, whereas shape changes at maximal size are displayed in orange. Shape variation is represented, from left to right and top to down, in anterior, medial, posterior, lateral, proximal and distal views. In the proximal view, the top corresponds to the posterior side; in the distal view, the top corresponds to the anterior side. Taxonomic abbreviations: Ad: *Adeopapposaurus*; Ba: *Barosaurus africanus*; Co: *Coloradisaurus*; Dc: *Dicraeosaurus*; Dp: *Diplodocus*; Gi: *Giraffatitan*; La: *Lapparentosaurus*; Lu: *Lufengosaurus*; Ma: *Massospondylus*; Ni: *Nigersaurus*; Pt: *Plateosaurus*; Pv: *Plateosauravus*; To: *Tornieria*; Un: *Unaysaurus*. CAC, common allometric component

A pooled regression was performed on bones for which the homogeneity of slopes was not rejected (i.e. humerus, radius, ulna, femur), in order to visualize the shape variation associated with their common allometry. Conversely, the allometric patterns for the groups in the tibia and fibula analyses are significantly different, preventing us from estimating a consistent common allometric component. The allometry for these bones was visualized separately for both groups. When size increases in the humerus (Figure 8), the main observed pattern is a mediolateral narrowing, especially of the shaft and distal end. On the proximal end, an increase in size is notably marked by a reduction of the medial tuberosity. A barely evident anteroposterior narrowing occurs. The posterior profile of the bone is slightly more sigmoid. Conversely, the lateral tubercle is wider and slightly more anteriorly oriented. The deltopectoral crest is more distally located, more medially oriented and with a more distally placed apex. The posterior olecranon fossa is deeper.

**FIGURE 9:**
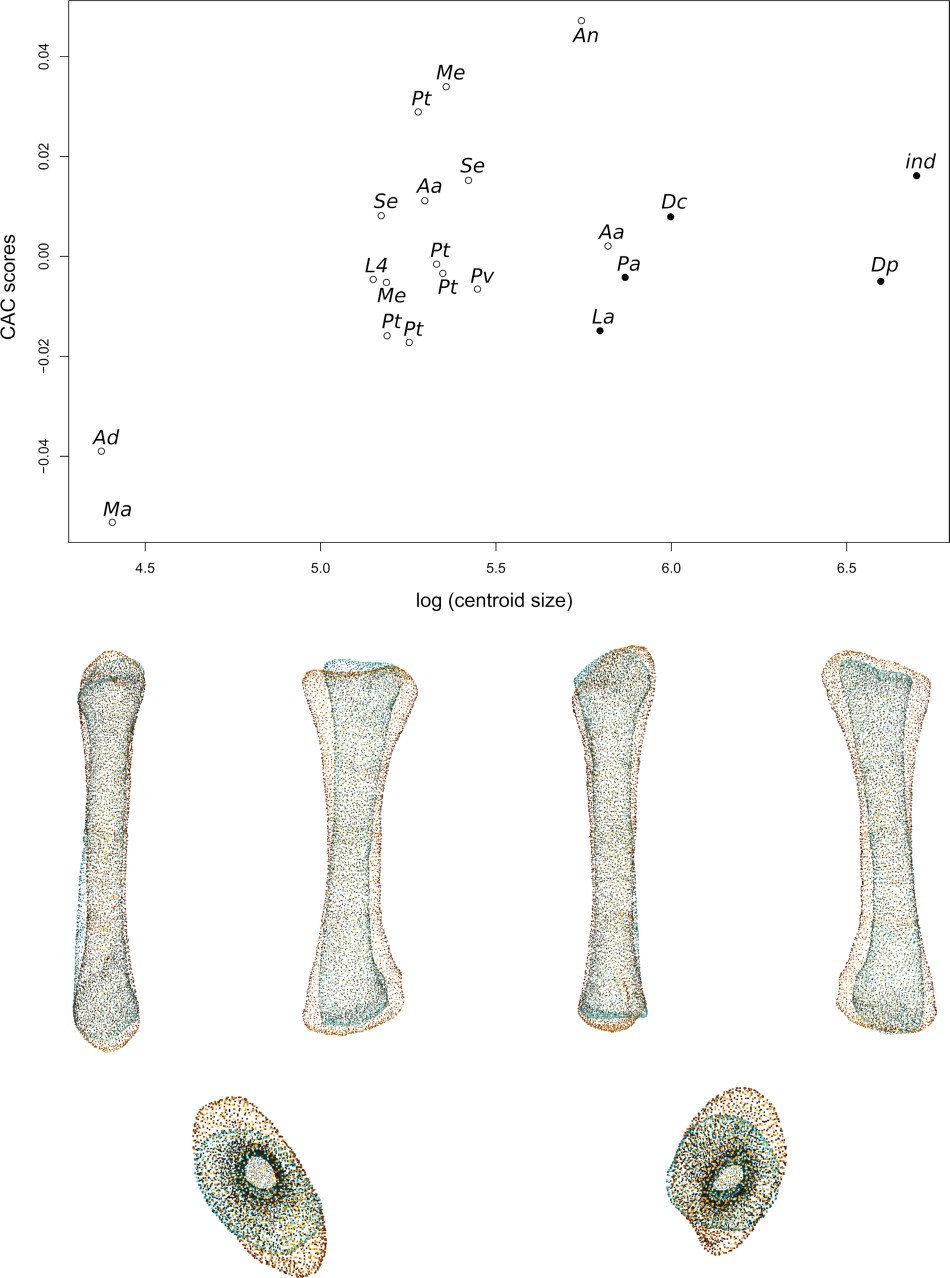
Allometric pattern of the radius: Top: bivariate plot showing the CAC scores against the natural logarithm of the centroid size. Open circles represent the non‐columnar sauropodomorphs and filled circles the columnar sauropods. Bottom: Thin‐plate splines visualizations of shape changes along with the CAC. Shape changes at minimal size are displayed in cyan, whereas shape changes at maximal size are displayed in orange. Shape variation is represented, from left to right and top to down, in anterior, medial, posterior, lateral, proximal and distal views. In proximal and distal views, the top corresponds to the anterior side. Taxonomic abbreviations: Aa: *Aardonyx*; Ad: *Adeopapposaurus*; An: *Antetonitrus*; Dc: *Dicraeosaurus*; Dp: *Diplodocus*; *ind: indeterminate sauropod from Tendaguru;* L4: MNHN.F.LES.400; La: *Lapparentosaurus*; Ma: *Massospondylus*; Me: *Melanorosaurus*; Pa: *Patagosaurus*; Pt: *Plateosaurus*; Pv: *Plateosauravus*; Se: *Sefapanosaurus*. CAC, common allometric component

In the radius (Figure 9), when size increases, the main observed pattern is a general anteroposterior widening, especially around the ends, and a mediolateral straightening of the shaft. A subtle mediolateral widening occurs.

**FIGURE 10:**
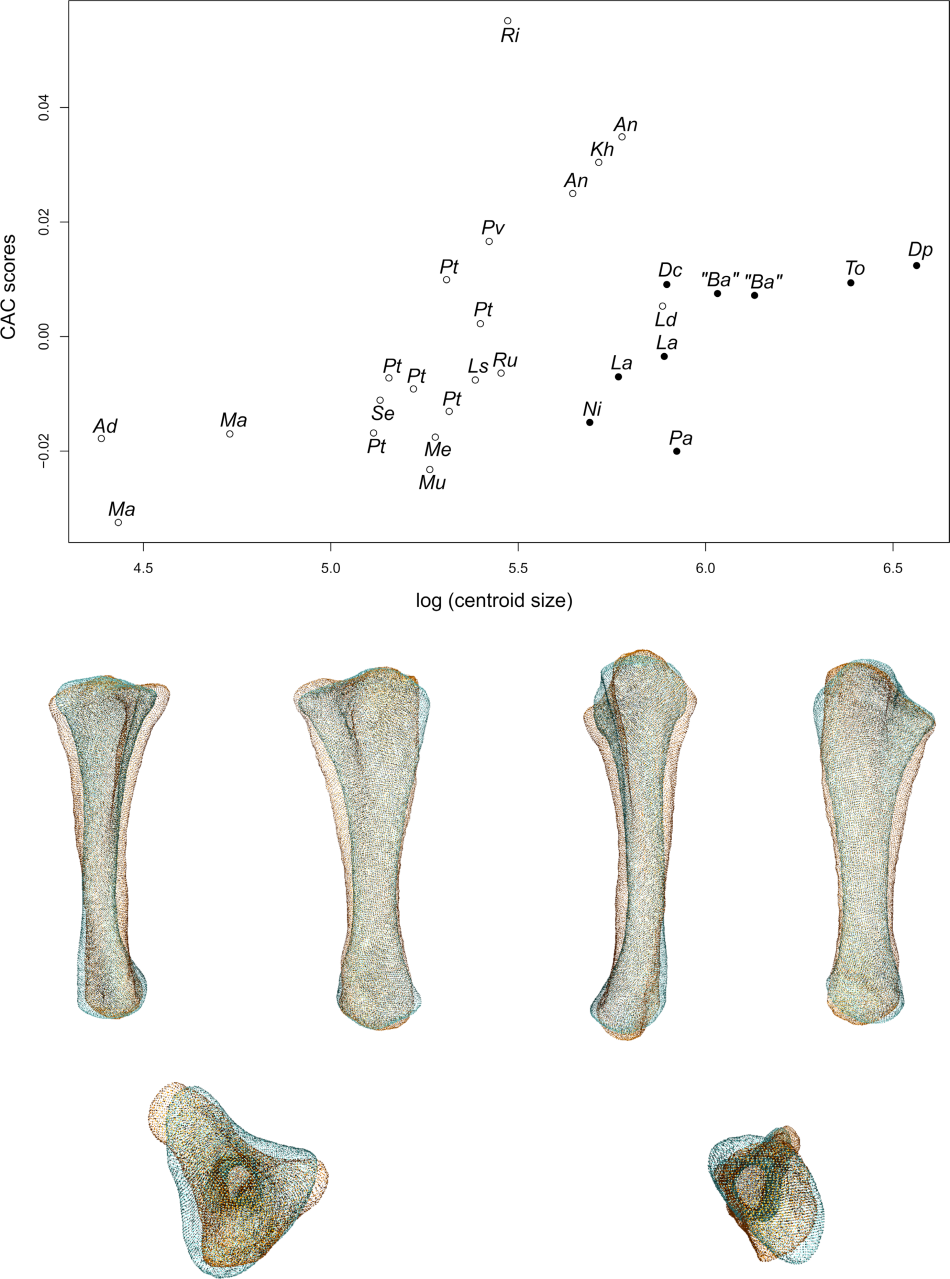
Allometric pattern of the ulna: Top: bivariate plot showing the CAC scores against the natural logarithm of the centroid size. Open circles represent the non‐columnar sauropodomorphs and filled circles the columnar sauropods. Bottom: Thin‐plate splines visualizations of shape changes along with the CAC. Shape changes at minimal size are displayed in cyan, whereas shape changes at maximal size are displayed in orange. Shape variation is represented, from left to right and top to down, in anterior, medial, posterior, lateral, proximal and distal views. In proximal and distal views, the top corresponds to the anterior side. Taxonomic abbreviations: Ad: *Adeopapposaurus*; An: *Antetonitrus*; Ba: *Barosaurus africanus*; Dc: *Dicraeosaurus*; Dp: *Diplodocus*; Kh: *Kholumolumo*; La: *Lapparentosaurus*; Ls: *Lessemsaurus*; Ma: *Massospondylus*; Me: *Melanorosaurus*; Mu: *Mussaurus*; Ni: *Nigersaurus*; Ri: *Riojasaurus*; Ru: *Ruehleia*; Pa: *Patagosaurus*; Pt: *Plateosaurus*; Pv: *Plateosauravus*; Se: *Sefapanosaurus*; To: *Tornieria*. CAC, common allometric component

When size increases in the ulna (Figure 10), no straightforward pattern of robustness increases or reduction is discernible: the proximal end becomes more slender, with a triradiate shape, that is with more marked concavities, radial fossa included, associated with a lengthening of the anterior and lateral processes, the olecranon is slightly more developed proximally. The shaft is proportionally more robust, following the anterior and lateral process projections development. The shaft is also slightly more curved mediolaterally. The distal end is reduced anteroposteriorly and slightly anteriorly twisted.

**FIGURE 11:**
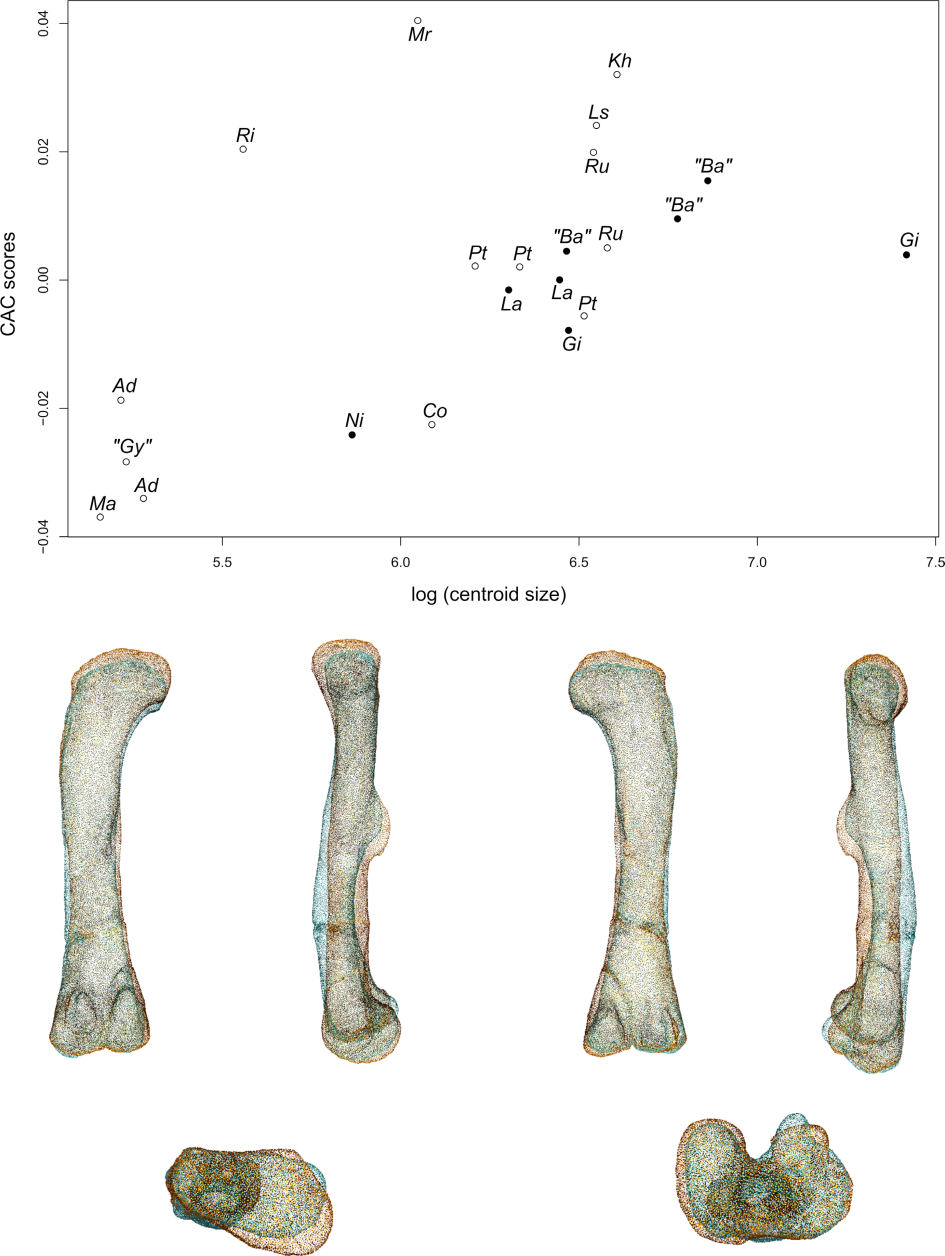
Allometric pattern of the femur: Top: bivariate plot showing the CAC scores against the natural logarithm of the centroid size. Open circles represent the non‐columnar sauropodomorphs and filled circles the columnar sauropods. Bottom: Thin‐plate splines visualizations of shape changes along with the CAC. Shape changes at minimal size are displayed in cyan, whereas shape changes at maximal size are displayed in orange. Shape variation is represented, from left to right and top to down, in anterior, medial, posterior, lateral, proximal and distal views. In proximal and distal views, the top corresponds to the posterior side. Taxonomic abbreviations: Ad: *Adeopapposaurus*; Ba: *Barosaurus africanus*; Co: *Coloradisaurus*; Gi: *Giraffatitan*; Gy: *Gyposaurus sinensis*; Kh: *Kholumolumo*; La: *Lapparentosaurus*; Ls: *Lessemsaurus*; Ma: *Massospondylus*; Mr: *Meroktenos*; Ni: *Nigersaurus*; Ri: *Riojasaurus*; Ru: *Ruehleia*; Pt: *Plateosaurus*. CAC, common allometric component

In the femur (Figure 11), when size increases, the shaft is straighter, and the fourth trochanter is more posteriorly and distally placed. The lesser trochanter is less anteriorly developed. The femoral head is more massive and slightly more anteromedially placed. A posterior development points out the posterior outline of the head. The distal end is slightly wider mediolaterally. The medial condyle is more developed anteroposteriorly and medially, the lateral condyle is more developed anteriorly and laterally. The intercondylar area is more developed anteroposteriorly.

**FIGURE 12:**
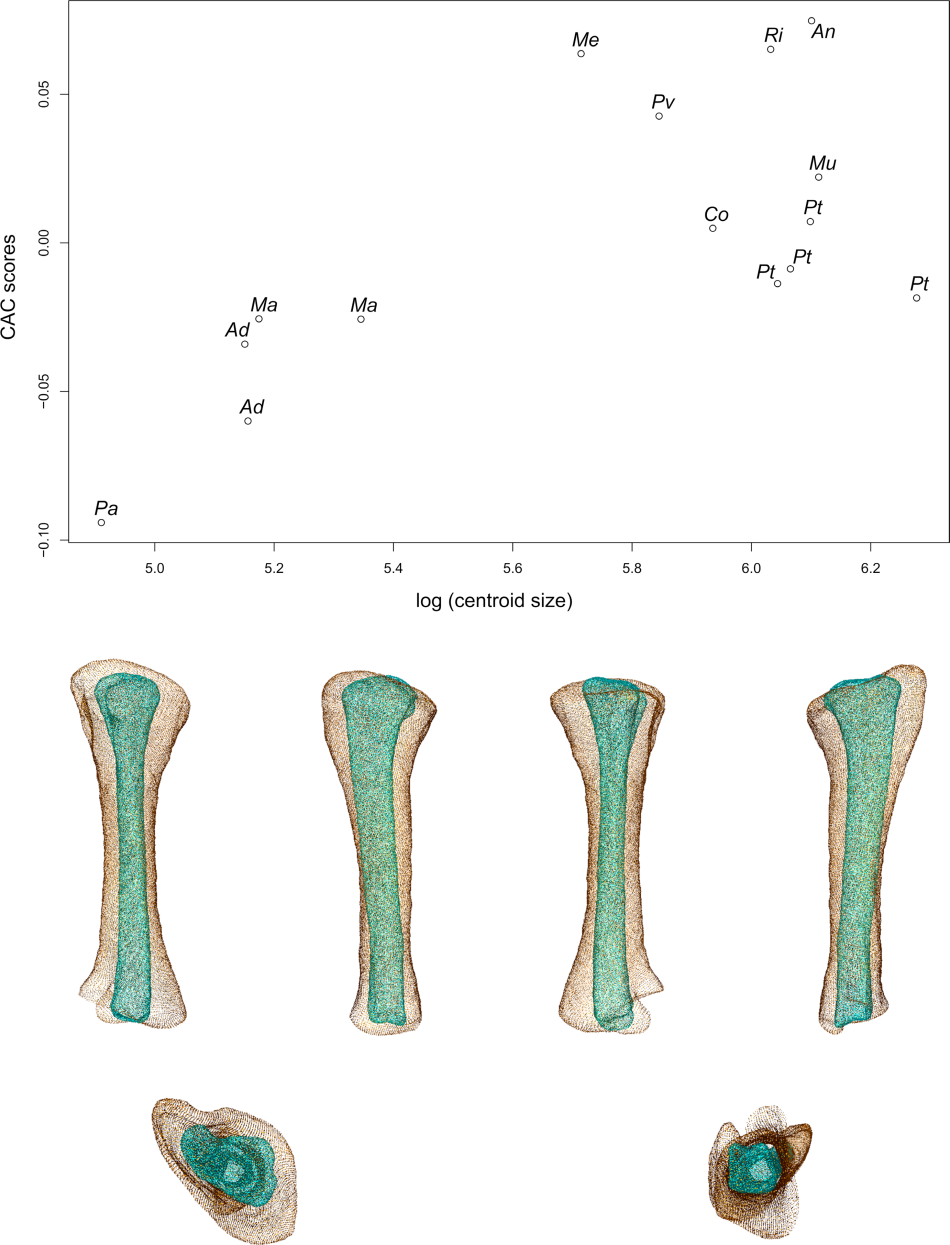
Allometric pattern of the tibia for non‐columnar non‐sauropodan sauropodomorphs: Top: bivariate plot showing the CAC scores against the natural logarithm of the centroid size. Open circles represent the non‐columnar sauropodomorphs and filled circles the columnar sauropods. Bottom: Thin‐plate splines visualizations of shape changes along with the CAC. Shape changes at minimal size are displayed in cyan, whereas shape changes at maximal size are displayed in orange. Shape variation is represented, from left to right and top to down, in anterior, medial, posterior, lateral, proximal and distal views. In proximal and distal views, the top corresponds to the lateral side. Taxonomic abbreviations: Ad: *Adeopapposaurus*; An: *Antetonitrus*; Co: *Coloradisaurus*; Ls: *Lessemsaurus*; Ma: *Massospondylus*; Me: *Melanorosaurus*; Mu: *Mussaurus*; Ri: *Riojasaurus*; Pa: *Panphagia*; Pt: *Plateosaurus*; Pv: *Plateosauravus*. CAC, common allometric component

When size increases, the allometric patterns in the tibia are different between the non‐columnar and the columnar sauropodomorphs. In non‐columnar sauropodomorphs (Figure 12), the tibia is proportionally considerably more robust when size increases, especially around the ends. Both proximal and distal ends are more oblique, with a slightly more laterally twisted proximal end, and, in the distal end, a more developed articular facet of the ascending process of the astragalus, descending process, and medial corner.

**FIGURE 13:**
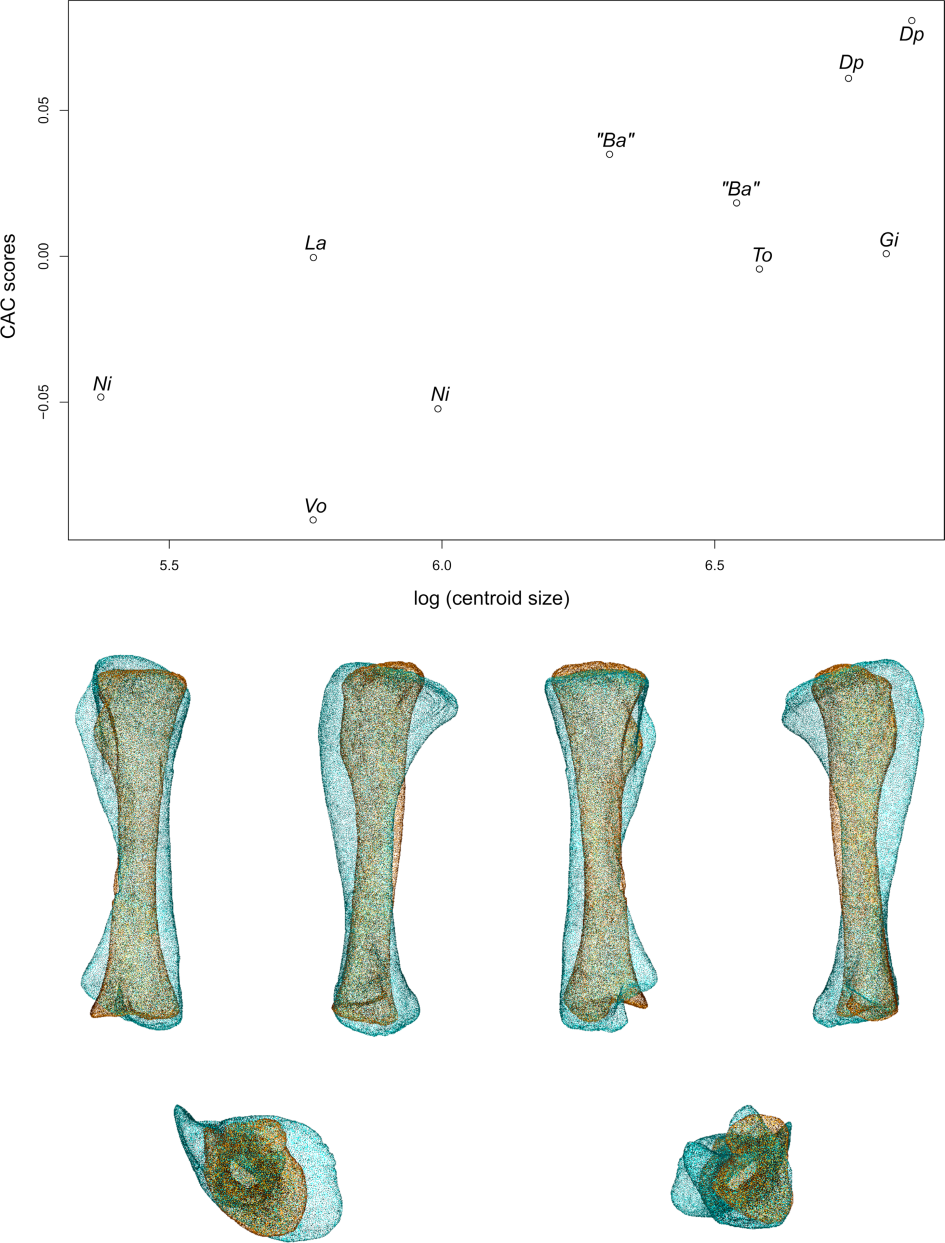
Allometric pattern of the tibia for columnar sauropods: Top: bivariate plot showing the CAC scores against the natural logarithm of the centroid size. Open circles represent the non‐columnar sauropodomorphs and filled circles the columnar sauropods. Bottom: Thin‐plate splines visualizations of shape changes along with the CAC. Shape changes at minimal size are displayed in cyan, whereas shape changes at maximal size are displayed in orange. Shape variation is represented, from left to right and top to down, in anterior, medial, posterior, lateral, proximal and distal views. In proximal and distal views, the top corresponds to the lateral side. Taxonomic abbreviations: Ba: *Barosaurus africanus*; Dp: *Diplodocus*; Gi: *Giraffatitan*; La: *Lapparentosaurus*; Ni: *Nigersaurus*; To; *Tornieria*; Vo: *Volkheimeria*. CAC, common allometric component

In columnar sauropods (Figure 13), the overall tibia is proportionally more slender, especially around the ends. Both the proximal and distal ends are flatter. The proximal end is more circular rather than elongated. The cnemial crest is less developed and is slightly more distally placed. The medial side and the lateral part of the proximal end contacting the fibula are developed, whereas the posterior part of the end is reduced. In the distal end, the articular facet of the ascending process of the astragalus is more developed relative to the rest of the end, whereas the descending process is reduced.

**FIGURE 14:**
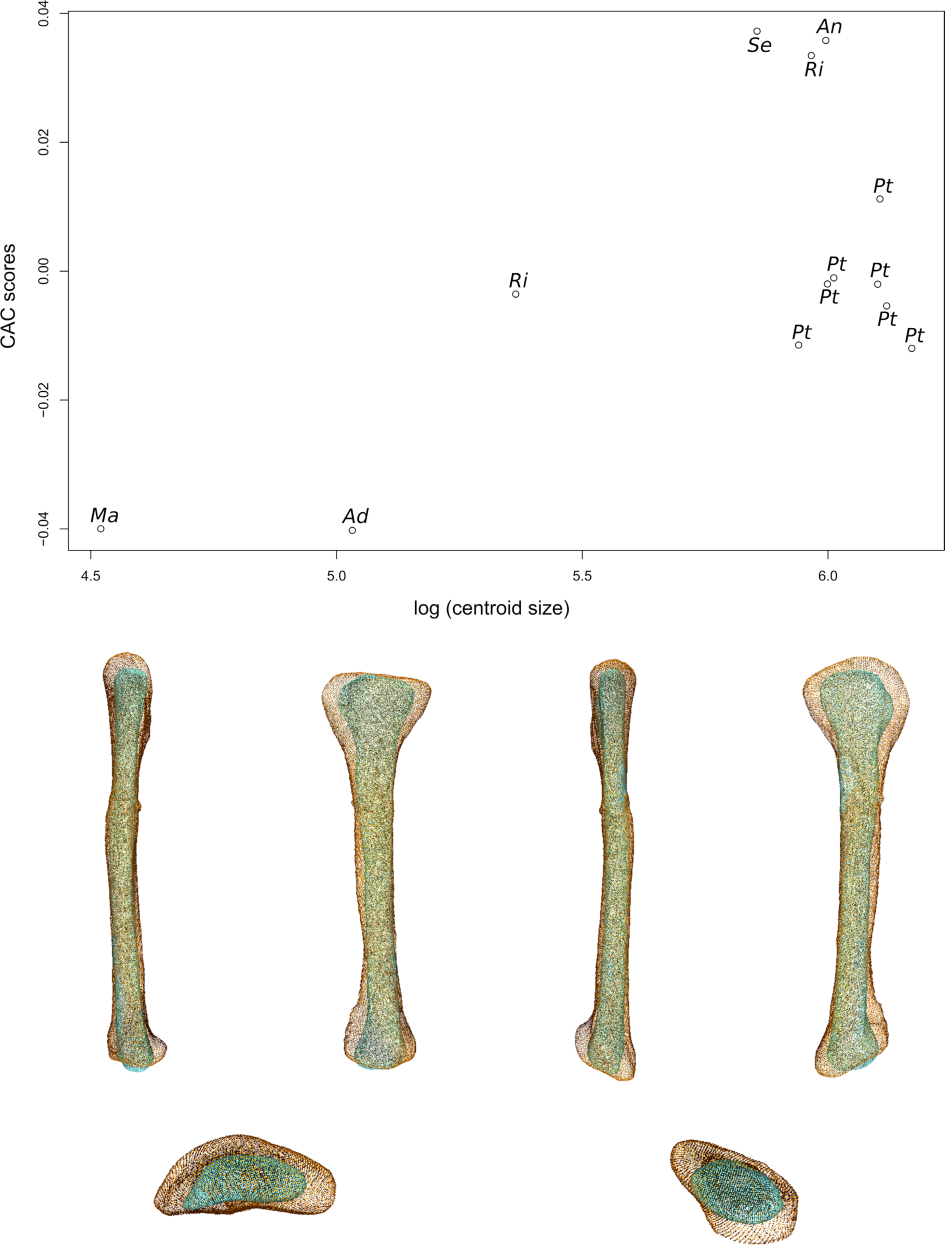
Allometric pattern of the fibula for non‐columnar non‐sauropodan sauropodomorphs: Top: bivariate plot showing the CAC scores against the natural logarithm of the centroid size. Open circles represent the non‐columnar sauropodomorphs and filled circles the columnar sauropods. Bottom: Thin‐plate splines visualizations of shape changes along with the CAC. Shape changes at minimal size are displayed in cyan, whereas shape changes at maximal size are displayed in orange. Shape variation is represented, from left to right and top to down, in anterior, medial, posterior, lateral, proximal and distal views. In proximal and distal views, the top corresponds to the lateral side. Taxonomic abbreviations: Ad: *Adeopapposaurus*; An: *Antetonitrus*; Ma: *Massospondylus*; Ri: *Riojasaurus*; Pt: *Plateosaurus*; Se: *Sefapanosaurus*. CAC, common allometric component. Note that the associated test of the effect of size on shape failed to reject the null hypothesis (cf. Table S3)

**FIGURE 15:**
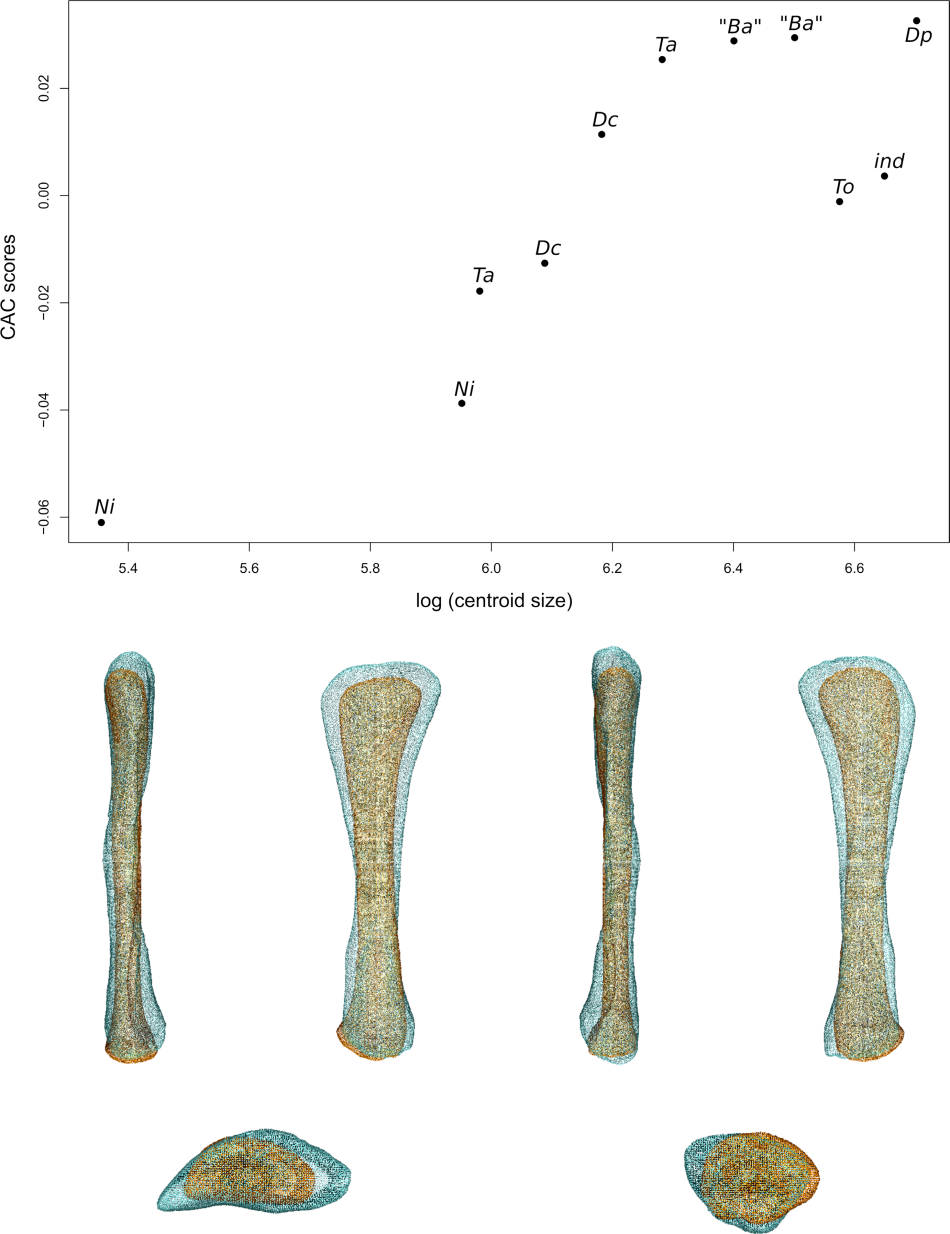
Allometric pattern of the fibula for columnar sauropods: Top: bivariate plot showing the CAC scores against the natural logarithm of the centroid size. Open circles represent the non‐columnar sauropodomorphs and filled circles the columnar sauropods. Bottom: Thin‐plate splinesvisualizations of shape changes along with the CAC. Shape changes at minimal size are displayed in cyan, whereas shape changes at maximal size are displayed in orange. Shape variation is represented, from left to right and top to down, in anterior, medial, posterior, lateral, proximal and distal views. In proximal and distal views, the top corresponds to the lateral side. Taxonomic abbreviations: Ba: *Barosaurus africanus*; Dc: *Dicraeosaurus*; Dp: *Diplodocus*; Gi: *Giraffatitan*; *ind: indeterminate sauropod from Tendaguru;* La: *Lapparentosaurus*; Ni: *Nigersaurus*; Ta: *Tazoudasaurus*; To; *Tornieria*. CAC, common allometric component

When size increases, the allometric patterns in the fibula are different between the non‐columnar and the columnar sauropodomorphs, following the same global pattern observed in the tibia. When size increases in the non‐columnar sauropodomorphs (Figure 14), the proximal end and the shaft are proportionally more robust, with a developed bump on the lateral side at midshaft. The distal end is more robust and oblique. In the columnar sauropods (Figure 15), when size increases, the overall fibula is proportionally more slender, the shaft is straighter, the lateral bump at midshaft is reduced. The distal end is slightly more developed anteriorly and noticeably less developed posteriorly.

## 4 DISCUSSION

### 4.1 Quantitative distinction of columnar‐limbed sauropods

Our analyses show that the distinction between the columnar‐limbed sauropods and the non‐columnar‐limbed non‐sauropodan sauropodomorphs is unambiguous for all phylomorphospaces. This observation is statistically supported since a significant difference in shape related to limb architecture is reported for every limb long bone (‘groups’ in Table S3). This differentiation relies on several morphological features already widely pointed out in the literature (Allain & Aquesbi, 2008; Carrano, 2005; McPhee & Choiniere, 2018; Rauhut et al., 2011; Salgado et al., 1997; Upchurch, 1998; Wilson, 2005a; Wilson & Sereno, 1998), such as the reduction of the deltopectoral crest, the olecranon process and the fourth trochanter, the development of a deep radial fossa, and the straightening of the femoral shaft. Our study quantifies the detailed anatomical features associated with the emergence of the sauropod limb bauplan.

#### 4.1.1 Forelimb features of columnar‐limbed sauropods

In sauropods, the forelimb is proportionally more slender, with less bulky and flatter proximal and distal ends. The shafts of the humerus and ulna are straighter, whereas that of the radius is slightly curved. In the humerus, in addition to the deltopectoral crest, the medial tuberosity is also considerably reduced. The anterior concavity is more acutely marked by an angled lateral tubercle, associated with a marked posterolateral ridge. The cuboid fossa is absent and replaced, roughly at the same position, by accessory condyles, and the olecranon fossa is markedly deeper. In the radius, the proximal and distal processes are absent. In the ulna, the olecranon process is reduced. In addition, the shape of the proximal end is triradiate (i.e. showing for each margin a noticeable concavity) and it shows a clearly U‐shaped radial fossa, characterized by the peculiar development of the anteromedial and lateral processes bracing the radial proximal end. The distal ends of the three bones are considerably reduced, and the angle of torsion between the proximal and distal ends is diminished, although the precise magnitude of variation should be taken with caution, given the sensitivity of this parameter to taphonomic processes (Lefebvre et al., 2020).

#### 4.1.2 Hindlimb features of columnar‐limbed sauropods

In sauropods, the hindlimb bones present globally flatter proximal and distal ends. The femoral shaft is straight, with considerably reduced lesser and fourth trochanters. The distal end shows a deep anterior intercondylar fossa. The femoral and fibular torsions are diminished. Although less wide, the cnemial crest of the tibia is still developed anterolaterally. The descending process is less developed laterally than the articular facet for the ascending process of the astragalus, which is anteroposteriorly thick. Although the overall distinction between clusters displayed in the phylomorphospaces and their associated features is clear and mostly congruent with the literature, a few minor discrepancies are noticeable. Some species show odd placements or wide distribution of their specimens in the phylomorphospace, which may be due to an error in the taxonomic attribution, ontogenetic variation, a possible residual taphonomic noise or a mixture of these three factors. However, the potential magnitude of a taphonomic bias in our study remains relatively limited, given our preliminary selection of the best‐preserved bones. Our analyses seem to provide useful information for tibiae, fibulae and probably for ulnae attributed to *B. africanus*. Indeed, most of these specimens plot close to the *Diplodocus*’ specimens, corroborating the previously assumed affinities of these particular specimens (Janensch, 1961; see Remes, 2009).

### 4.2 Evolution of the sauropod limb bauplan

The phylomorphospace patterns are similar between the radius and the ulna and between the tibia and the fibula. These observations suggest integrated evolutionary scenarios within the pair of bones of each zeugopod, but different ones between forelimb and hindlimb couples. The evolutionary scenarios suggested by the phylomorphspace patterns for the stylopod bones are less clear, probably due to the scarcer taxonomic sampling for these two bones.

#### 4.2.1 Humerus

For the humerus, a rather clear separation exists between the non‐sauropodiform sauropodomorphs and the sauropod clusters. Also, some smaller morphological changes (e.g. thinner deltopectoral crest, slightly more slender and sigmoidal shaft) clearly separate the Plateosauridae from the other non‐sauropodiform sauropodomorphs and, more putatively, *Giraffatitan* from the other sauropods.

The sauropod humerus is considerably different from the non‐sauropodan sauropodomorph one, notably by the reduced deltopectoral crest, which necessarily implies less space for insertion sites of *pectoralis* and *deltoideus clavicularis* muscles, notably involved in the abduction, adduction and flexion (Otero, 2018). Despite this reduction, the proximolateral region, comprising the lateral tubercle, is still developed. The deltopectoral crest is acutely bent, with a complementary developed posterolateral ridge. This area is the probable insertion site of the *latissimus dorsi* and the *teres major* muscles, which are involved in the extension and the supination of the bone (Christiansen, 1997; Otero, 2018). The presence of this crest thus hints at a substantial involvement of these muscles in sauropod locomotion. The medial tuberosity is much reduced in sauropods, suggesting a correlated diminution of the action of the *subcoracoscapulares* muscles, involved in adduction and humeral pronation (Otero, 2018). The shaft of the sauropod humerus is straighter and shows nearly no torsion. As for the other bones showing this condition, the straightening of shafts is related to the management of mechanical stresses involved in weight support and, as in large mammals, is probably associated with a diminution of the locomotor repertoire (Carrano, 2001). Similarly, to what is seen in some other bones (e.g. femur, see Carrano, 2000) a reduced humeral torsion (i.e. smaller angle between the long axes of the proximal and distal ends) may have an impact on the role of muscles originating or inserting in the affected area, such as the numerous muscle origins located in the humeral distal end (see Otero, 2018). Distally, our analyses highlight the deepening of the olecranon fossa in sauropods. In extant archosaurs, this fossa receives the intercotylar process (Fujiwara et al., 2010) and not the olecranon process, which is the insertion site of the *m. triceps brachii* (Fujiwara et al., 2010). Hence, the deepening of the olecranon fossa does not directly signify an amelioration of the extension capabilities of the sauropod forelimb; moreover, considering the reduction of the olecranon process (Carrano, 2005), the extension capabilities of the forelimb may have even been decreased (Christiansen, 1997). However, as the deepening of the olecranon fossa is marked in a relatively large portion of the shaft, it probably left more space for the *triceps brachii* muscles. An increase or the conservation of efficient extension capabilities in the sauropod forelimb is, thus, plausible. Conversely, the loss of the cuboid fossa and the development of distal condyles have probably drastically diminished the flexion possibilities of sauropods (Ballell et al., 2022; Bonnan, 2003; Bonnan & Senter, 2007). Our observations suggest that at least one evolutionary episode of strong morphological changes occurred, which requires the input of key taxa in order to be confirmed. The qualitative observation of incomplete humeri of *Antetonitrus* (BP/1/4952; see McPhee et al., 2014), *Ingentia* (PVSJ 1086; closely related to *Antetonitrus* in Apaldetti et al., 2018) and *Tazoudasaurus* (MHNM To1‐380 and 93; see Allain & Aquesbi, 2008) refines the characterization of the evolution of the sauropod humerus. The first two taxa indeed share numerous traits with non‐sauropodiform sauropodomorphs, notably a relatively high humeral torsion. Although sensitive to taphonomic distortion (Lefebvre et al., 2020), this feature recalls the condition of the largest sampled *Plateosaurus* specimen (SMNS 80664; see Galton, 2001; Lefebvre et al., 2020), which is the largest non‐sauropodiform sauropodomorph of our humerus analysis, possibly suggesting a common, size‐related trend within non‐sauropodan sauropodomorphs. Conversely, the humerus of *Tazoudasaurus* shows affinities with the other sauropod humeri, such as a reduced deltopectoral crest, no humeral torsion, no cuboid fossa and a deep olecranon fossa. These observations suggest a rapid emergence of the sauropod humerus, marked by strong changes only found in sauropods. The integration of additional complete and well‐preserved specimens of non‐sauropodan sauropodiforms and early sauropods into the quantitative analysis would add robustness to this inference.

#### 4.2.2 Forelimb zeugopod

Both radius and ulna phylomorphospaces show a clear separation between the sauropod and the non‐sauropodan sauropodomorphs clusters. Within non‐sauropodan sauropodomorphs, non‐sauropodan sauropodiforms and non‐sauropodiform sauropodomorphs show some overlap. Non‐sauropodan sauropodiforms are differentiated from non‐sauropodiform sauropodomorphs principally by their variation in robustness and, for the ulna, by a more marked radial fossa and lateral process. The non‐sauropodan sauropodiforms are more distant from the sauropods than the non‐sauropodiform sauropodomorphs. The sauropod cluster is, in both analyses, very distant from the non‐sauropodan sauropodomorph taxa. The ulnae are proportionally markedly more slender in sauropods than in non‐sauropodan sauropodomorphs. They show a clearly triradiate and U‐shaped ulnar proximal end bracing the reduced radial proximal end. They also show reduced distal ends. In both analyses, the disparity of the sauropod cluster is relatively small, indicating little morphological variation among the specimens of this clade, despite the sampling of early taxa (*Patagosaurus*, *Lapparentosaurus*), suggesting that no straightforward stepwise process is discernible in our analyses of the evolution of the sauropod antebrachium. Its emergence therefore likely took place in a short period of time.

The qualitative observation of incomplete bones of *Tazoudasaurus* tends to corroborate this scenario. The ulnae (MHNM To1‐374 and Pt‐24; see Allain & Aquesbi, 2008; Rauhut et al., 2011) show a slender profile as in the sampled sauropods. The triradiate condition of the proximal ulnar end has been pointed out by several authors (e.g. Wilson & Sereno, 1998; Yates et al., 2010) as a keystone feature characterizing the obligatory quadrupedality of sauropods. This feature contributes to the tight association of the ulna and the radius (Bonnan, 2003; Wilhite, 2003, 2005; Wilson & Sereno, 1998). Yates et al. (2010) proposed that this triradiate shape originated in non‐sauropodan sauropodiforms. According to Yates et al. (2010), a triradiate condition was incipient in *Aardonyx*; it was also observed later in *Mussaurus* (Otero & Pol, 2013). This so‐called incipient condition is based on the observation of a lateral process, associated with a supposed progressive deepening of the radial fossa. Hence, the evolution of this feature has been proposed to possibly represent an intermediate trend of facultative or habitual quadrupedality in non‐sauropodan sauropodomorphs (McPhee et al., 2014; McPhee & Choiniere, 2018; Otero et al., 2017), in the context of a mosaic evolution towards the sauropod forelimb (Bonnan & Yates, 2007; Yates et al., 2010). The presence of this feature in taxa inferred as habitually biped such as *Mussaurus* have also led to suggest that this feature could have been non‐adaptive and co‐opted for obligatory quadrupedality in sauropods (Otero & Pol, 2013). However, the interpretation of this development and its comparison with the sauropod condition has been recently discussed (Peyre de Fabrègues, 2016; see also McPhee & Choiniere, 2018).

Our results highlight a more complex diversity of the ulnar proximal end. If the lateral process is developed in sauropods, it is also well incurved anteriorly. The anteromedial process is also considerably more developed, thin and incurved anteriorly, giving a characteristic U‐shape to the radial fossa, firmly bracing the radial proximal end. This condition is not seen in non‐sauropodan sauropodomorphs: the anteromedial process remains in most cases thick but poorly *expanded* anteriorly and is either rounded (e.g. in *Mussaurus*, *Melanorosaurus*, *Ledumahadi*, *Lessemsaurus*) or subtriangular (e.g. in *Plateosaurus*, *Ruehleia*, *Adeopaposaurus*). *Antetonitrus* (see McPhee et al., 2014) and *Sefapanosaurus* (see Otero et al., 2015) show an anteromedial process more developed but not incurved anteriorly, as observed in sauropods. Some non‐sauropodiform sauropodomorphs also show a subtle radial fossa (e.g. *Rojasaurus*, *Kholumolumo*, some specimens of *Plateosaurus*; see also e.g. *Plateosaurus* in Mallison, 2010b and *Massospondylus* in Barrett et al., 2019) and, although not necessarily well developed, their lateral process is also noticeable. In ornithischian dinosaurs, Maidment and Barrett (2012) showed that the development of the lateral process, although correlating well with posture, may also be related to size, given the lack of size overlap between unambiguous bipeds and quadrupeds. This might also be the case for sauropodomorphs, since the results of our analysis of the ulnar allometry tend to show that the deepening of the radial fossa is size related. These elements, although not necessarily contradicting the functional interpretations made on the deepening of the radial fossa, suggest that this morphological feature alone is not sufficient to estimate the locomotor abilities of sauropodomorph taxa. Indeed, a substantial gap exists between non‐sauropodan and sauropod forms. The shaft of the ulna in sauropods is considerably slender, almost straight, and the distal end is also considerably reduced. Combined with the U‐shaped proximal end, it results in a tight association with the radius, so that the posterior surface of the radius is nearly completely contacting the ulna proximal and distal thirds (Bonnan, 2003; Wilhite, 2003). This close association is not observed in non‐sauropodan sauropodomorphs, where the shafts and distal ends are not or poorly contacting (Apaldetti et al., 2018; Bonnan & Senter, 2007; Mallison, 2010a, 2010b; Otero et al., 2017). Conversely, the radial and ulnar distal ends in neosauropods are coalescent (Hutson, 2015). Following Hennig (1925) arguing that this particular condition permitted more support, Hutson (2015) added that this feature, convergently observed in stegosaurs, may be correlated with the development of a semi‐tubular manus, allowing more efficient vertical weight‐bearing with a not fully pronated manus. This hypothesis is consistent with the fossil track record (Goussard, 2009; Lallensack et al., 2019; Wilson, 2005a; Xing et al., 2015). The distal end of the ulna of *Tazoudasaurus* (MHNM To1‐374; qualitative observation) is more elongated than in most sampled sauropods (e.g. *Tornieria*, *Diplodocus*), which would thus imply a more elongated manus articulation. This is consistent with the observation that the manus of *Tazoudasaurus* is not semi‐tubular (Allain & Aquesbi, 2008). However, a similarly elongated distal ulna is also seen, in our sample, in *Patagosaurus* and *Nigersaurus*. This fact implies that this morphological feature may be plesiomorphic and show convergence and/or reversion within sauropods, or that these observations are made on specimens of relatively small size. The relatively reduced shape of the radius in these two sauropods is, however, considerably differentiating them from non‐sauropodan sauropodomorphs.

All these aspects tend to confirm a profound difference between the non‐sauropodan and the sauropod antebrachium, with associated changes in their manus. The non‐sauropodan sauropodiforms, often considered potential locomotor intermediates between the habitually bipedal non‐sauropodiform sauropodomorphs and the obligatory quadrupedal sauropods (e.g. Bonnan & Yates, 2007; Yates et al., 2010), show a majority of functional traits of the non‐sauropodiform sauropodomorphs. Despite a more marked radial fossa, their ulnae display a robust overall shape, with a medially curved shaft, and a poorly developed contact area with the radius (e.g. in *Ingentia* PVSJ 1087, qualitative observation of the connected antebrachium; see Apaldetti et al., 2018). Thus, these results suggest that any assessment putting on the same functional level the locomotor habits of non‐sauropodan sauropodiforms and sauropod taxa (e.g. McPhee et al., 2018) should be regarded with caution. Indeed, no study has been performed on the locomotor performances of an inferred quadrupedal non‐sauropodan sauropodiform, testing the viability of habitually quadrupedal locomotion in such taxa. Quantified biomechanical studies estimating the performance of such taxa, comparatively to sauropods, would refine our knowledge of the locomotor diversity in sauropodomorphs.

#### 4.2.3 Femur

For the femur, the sauropod cluster is well separated from the non‐sauropodan sauropodomorph one, although to a lesser extent than in the forelimb zeugopod analyses. No gradient pattern is discernible. The non‐sauropodiform sauropodomorphs, the non‐sauropodan sauropodiforms and the sauropods are well separated. However, this separation may be an artefact of the low number of sampled non‐sauropodan sauropodiforms (e.g. lack of *Antetonitrus*) and early sauropods (e.g. lack of *Tazoudasaurus*, *Volkheimeria*). It does not allow an accurate estimate of the occupation of their respective cluster in the phylomorphospace. This sample issue hinders an assessment of the evolution of the femoral shape with the same level of confidence as for the forelimb (see above) and hindlimb (see below) zeugopod analyses. Moreover, the delimitation of two specimens of *B. africanus* is similar to what is observed in the hindlimb zeugopod (see below), assuming that the involved specimens belong to the same genus. These two specimens show a rather slender shaft, contrasting with the more eccentric (i.e. elliptical in cross section) pattern seen in other sauropods.

The femur is generally interpreted as a keystone element in the emergence of the sauropod bauplan. Indeed, the progressive increase of the straightening and the eccentricity of the femoral shaft, as well as the reduction and displacement of the femoral trochanters (e.g. Carrano, 2005; McPhee & Choiniere, 2018; Wilson & Sereno, 1998; Yates et al., 2010) have been highlighted as strongly related to the emergence of the sauropod columnar limb. Our analyses show that the sauropod femur is markedly different from that of non‐sauropods. In sauropods, the shaft is near completely straight in the lateral view. Such a condition is not seen in most non‐sauropodan sauropodomorphs, with the noticeable exception of the non‐sauropodiform sauropodomorph *Ruehleia* (see Lefebvre et al., 2020) and, to a lesser extent, in the non‐sauropodan sauropodiform *Meroktenos* (see Peyre de Fabrègues & Allain, 2016). Outside of the specimens included in our analysis, the non‐sauropodan sauropodiform *Camelotia* also shows such a straight shaft (Galton, 2005).

The femoral head is anteromedially to medially oriented in non‐sauropodan sauropodomorphs, whereas it is strictly medially oriented in sauropods. This change in orientation is coupled in sauropods with an enlargement of the ilium, particularly in its anterior part (Carrano, 2000, 2005), contributing to a shift in the locomotor abilities of this group, where the predominance of protraction and retraction would have been increased over abduction and adduction (Carrano, 2000). Sauropods also show a lateral migration of the lesser trochanter, giving a lateral position to the *iliofemoralis externus*, which retain a role in abduction (Carrano, 2005). Moreover, the distal displacement of the fourth trochanter participates in longer lever arms of the *caudofemoralis* muscles (Ballell et al., 2022; Carrano, 1999; Gatesy, 1990, 1995; McPhee & Choiniere, 2018; Persons & Currie, 2019), resulting in slower movements but involving more force (Ballell et al., 2022; Bonnan, 2007; Carrano, 1999; Yates et al., 2010), and the increase of eccentricity results in femora larger mediolaterally than anteroposteriorly, given that less skeletal bending resistance is needed in this last direction (Wilson & Carrano, 1999). Our analyses however show that these two morphological features are also seen in the non‐sauropodan sauropodiforms sampled, *Lessemsaurus* and *Meroketnos*, which noticeably show an even more distal position of the fourth trochanter than in sauropods. The distal end of the sauropod femur noticeably differs from that of the non‐sauropodan sauropodomorphs, because the condyles are only connected by a thin transversal ridge, the anterior part being marked by a pronounced intercondylar fossa.

Our results show a clear variation in the locomotor abilities between non‐sauropodan sauropodomorphs’ and sauropods’ femora, by the morphological features described above. They are also present in the early sauropod *Tazoudasaurus* (MHNM To1‐105, 256, 381; qualitative observation; see Allain & Aquesbi, 2008). However, several of these features are also punctually observed in some non‐sauropodan sauropodomorph taxa. Notably, the distal location of the fourth trochanter and the increased eccentricity of the shaft are observed in the sampled non‐sauropodan sauropodiforms (*Lessemsaurus*, *Meroktenos*), which suggest a stepwise evolution of the sauropod femur. However, other features, such as the straight shaft, observed in a non‐sauropodiform sauropodomorph (i.e. *Ruehleia*) indicate a certain degree of homoplasy in the evolution of the femoral features across sauropodomorph evolution. Indeed, femoral straightening is already known to be convergently found across dinosaurs (Carrano, 2000). However, columnar sauropods constitute the sole clade to show the combination of all the features discussed in this subsection, in addition to the medial orientation of the femoral head, the lateral position of the lesser trochanter and the anterior intercondylar fossa in the distal end. Our results suggest a mosaic nature of the evolution of the sauropod femur, with some features appearing before the emergence of the columnar limb, and a substantial number of other features appearing at the node Sauropoda. Given the substantial part of homoplasticity for this bone, a stabilization of the phylogenetic framework is essential in order to disentangle the sequence of steps that led to the emergence of the sauropod femur.

#### 4.2.4 Hindlimb zeugopod

For the hindlimb zeugopod, the specimens are distributed along with a curved gradient globally congruent with phylogenetic relationships. In non‐sauropodan sauropodomorphs, the morphology of both tibia and fibula is roughly stable, even when considering the earliest taxon of the group (*Panphagia*). A continuous shape variation occurs across the entire gradient: a proportional increase in robustness is observed, with relatively slender forms in non‐sauropodiform sauropodomorphs (e.g. *Panphagia*, *Plateosaurus*, *Massospondylus*) to noticeably more robust forms in some non‐sauropodan sauropodiforms (such as *Antetonitrus*) more closely related to sauropods. Sauropods are markedly morphologically different from non‐sauropodan sauropodomorphs but are also showing intra‐group variation. Some early sauropods (e.g. *Tazoudasaurus*, *Volkheimeria*) plot close to non‐sauropodan sauropodiforms and show a similar high degree of robustness. Conversely, a decrease in robustness is observed in other sauropods (e.g. *Lapparentosaurus*, *Giraffatitan*, *Tornieria*), with an extremum in *Diplodocus* and some of the specimens attributed to *B. africanus* and to *Dicraeosaurus*. If the slender specimens attributed to *B. africanus* were correctly identified (as belonging to the genus *Barosaurus*), a large part of the trend depicted here could be influenced by these specimens and those attributed to *Diplodocus* and to *Dicreaosaurus*. Thus, this trend could represent an evolutionary episode more nested in Diplodocoidea rather than occurring through all sauropods. This inversion of trend in the robustness of the hindlimb zeugopod throughout sauropodomorph evolutionary history is also found in our allometry analysis, which shows a shift in allometric trend between the non‐sauropodan sauropodomorphs and the sauropods (see below).

Our analyses seem to delineate successive steps in the evolution of the sauropod columnar hindlimb zeugopod. The proximal end of the tibia changes from an anteroposteriorly expanded and oblique surface, to a more circular and straight one (e.g. see *Diplodocus* in Hatcher, 1901). The bulky cnemial crest seen in non‐sauropodan sauropodomorphs is consistently reduced in its width but still anterolaterally developed in sauropods. Ballell et al. (2022) also recently pointed out the particular prominence of the cnemial crest in non‐sauropod plateosaurians and in *Efraasia*, differing from the proximally less pronounced condition seen in sauropods. In sauropods the anterolateral development varies, from being slightly to strongly developed. The cnemial crest is more distally placed along the shaft, so that the apex is below the proximal end surface in lateral view, in taxa such as *Lapparentosaurus* (see Ogier, 1975), *Tornieria* (see Remes, 2006), *B. africanus* (see Remes, 2009). Despite the degree of anterolateral development and the distal displacement, all sampled sauropods display a cnemial crest expanded along the long axis of the shaft. A similar condition (cnemial crest still developed anterolaterally but reduced in width) is observed in *Tazoudasaurus*, the earliest sauropod of our analysis (MHNM To1‐76 and 380; qualitative observations; see Allain & Aquesbi, 2008), although the top of the crest is at the same level as the proximal surface. It is, moreover, still very proximal, but less anterolaterally developed, in *Volkheimeria*. These conditions seen in the earliest sauropod taxa of our sample suggest that the distal displacement of the cnemial crest along the shaft occurs after the emergence of the sauropod clade. The sauropod condition of the cnemial crest (still developed in its long axis and projected anterolaterally) should have conserved a substantial muscle insertion site, despite the reduction in width. It is thus plausible that the extensor muscles attached to this crest were still substantially represented, and hence, significantly involved in locomotion, but also in other plausible movements such as rearing (Mallison, 2011). Any conclusions about non‐locomotor movements are, however, too preliminary at this stage, and would require further study. In addition, the distal displacement of the cnemial crest would have provided longer lever arms, generating more power per stride (Carrano, 1999; Hildebrand, 1982). Therefore, the knee extension capabilities of the sauropods probably have retained a more important role in their movements than previously stated (Carrano, 2005). Our interpretations are congruent with a recent biomechanical study finding stronger moment arm in knee flexion and extension in sauropods than in the non‐sauropodiform sauropodomorph *Plateosaurus* (Klinkhamer et al., 2018). In its distal end, the descending process of the tibia is markedly less developed, as pointed out in Wilson and Sereno (1998) and especially Yates (2004). This condition is also seen in *Antetonitrus* (see McPhee et al., 2014) and in a specimen attributed to *Melanorosaurus* (BP/I/5090), despite little damages. The articular facet of the ascending process is, however, anteroposteriorly massively developed in sauropods only. *Tazoudasaurus* (MHNM To1‐380, qualitative observation) also shows this combination of a massively developed articular facet and a poorly developed descending process. This variation seems tightly correlated with the stepwise evolution of the astragalus in sauropodomorphs (Bonnan, 2005; Wilson & Sereno, 1998). A noticeable exception to this combination of features is the two sampled specimens of *Nigersaurus*, showing a thin descending process and a thinner articular facet of the ascending process, both of them equally developed laterally.

The fibula, like the tibia, follows a similar trend of increase and decrease in shaft robustness, also associated with an allometric shift episode (non‐sauropodan sauropodomorphs' fibulae are more robust in relation to the size increase, whereas the opposite occurs in sauropods). The proximal end of the fibula in sauropods is considerably different from that in non‐sauropodan sauropodomorphs. Sauropods notably show a characteristic triangular medial scar (Allain & Aquesbi, 2008; Wilson & Sereno, 1998), which is also plausibly seen in *Antetonitrus*, and more ambiguously in some other non‐sauropod sauropodomorphs such as *Adeopapposaurus*. This suggests that this particular scar appeared in sauropodomorph evolutionary history before the emergence of Sauropoda. Near the midshaft, non‐sauropodan sauropodomorphs display an anteromedial to anterior depression, interpreted as the insertion of the *iliofibularis* muscle (Langer, 2003; Lefebvre et al., 2020; McPhee et al., 2014). A lateral bump is observable in some non‐sauropodan sauropdomorphs and in sauropods (e.g. ‘lateral tuberosity’ in *Sefapanosaurus* [Otero et al., 2015]; see also Cooper, 1981 and McPhee et al., 2014 for other non‐sauropodan‐sauropodomorphs; ‘lateral trochanter’ in sauropods in Wilson & Sereno, 1998 and Allain & Aquesbi, 2008). In all sampled sauropods except *Tazoudasaurus* this lateral bump is proximodistally covered by a flattened to shallow and oblique depression. This depression is interpreted by some authors as being the insertion of the *iliofibularis* (Powell, 1992; Wilhite, 2003), which would traduce in a distal displacement of this muscle, allowing a longer lever arm than the more proximal plesiomorphic insertion. However, other authors (Borsuk‐Bialynicka, 1977; Wilson & Sereno, 1998) interpreted this area as the origin of the *flexor digitorum*. As no clear anterior depression is discernible in the sampled sauropods, and given the mechanical advantage provided by a more distal muscle insertion (Wilhite, 2003), we consider the first hypothesis more likely. The absence of depression in *Tazoudasaurus* does not permit firm conclusions on the evolution of the muscle scars on the fibular midshaft.

To summarize, our results show that the evolution of the sauropod limb bauplan was complex and not uniform. Our analyses highlight a marked morphological gap between the sauropod and non‐sauropodan sauropodomorph limb long bones, except for the fibula. It strongly suggests that the setting of many characteristic morphological features of sauropods (e.g. reduced and pinched medial tuberosity, U‐shaped ulnar proximal end, absence of radial proximal and distal processes, medially oriented femoral head, reduced cnemial crest width), took place in a very short period of time. These changes happened before the end of the Early Jurassic, given the age and placement of the earliest sampled sauropods in the phylomorphospaces (*Patagosaurus* and *Volkheimeria* have been recently dated from the Toarcian [Cúneo et al., 2013; Pol et al., 2020]). Our qualitative analyses of *Tazoudasaurus* limb bones corroborates this affirmation because this taxon is found in the Pliensbachian‐Toarcian of Morocco (Allain & Aquesbi, 2008). These changes likely contributed to the success of the sauropod bauplan, following the faunal turnover that occurred during the Pliensbachien‐Toarcian leading to the dominance of these taxa in post‐Toarcian ecosystems (Allain & Aquesbi, 2008; Apaldetti et al., 2021; Pol et al., 2020).

However, the evolution of some morphological features appears not to be directly linked to this evolutionary episode. Indeed, several features classically associated with the sauropod limb bauplan, such as the femoral shaft straightness or the distal location of the fourth trochanter, occurred earlier than the node Sauropoda in sauropodomorph evolutionary history. Other traits evolved in a more stepwise way, diversifying the observed patterns. This is the case for the evolution of the robustness in the hindlimb zeugopod, the position and development of the cnemial crest, and the shape of the distal ulnar end. Therefore, we interpret the emergence of the sauropod overall limb bauplan as a mosaic evolutionary episode, with a complex pattern of rates of morphological changes. The inspection of the detailed morphological features depicts a differential evolutionary scenario for the forelimb and the hindlimb. Indeed, the forelimb tends to show an abrupt appearance of traits associated with sauropods (contrasting with the more progressive evolution previously assessed for this limb [Bonnan & Yates, 2007; Yates et al., 2010]), whereas the hindlimb includes a larger number of morphological features evolving in a stepwise fashion, although it also displays some strong and rapid changes. This statement is made with a high degree of confidence for the zeugopods, whereas the lack of key taxa in stylopod analyses slightly lessens the degree of confidence in this assertion for these bones.

### 4.3 Allometric patterns in the variation of body size in sauropodomorphs

A significant impact of size on shape is observed in our analyses (Table S3). It is unambiguous (*p* < 0.05) for the forelimb bones and the tibia. The test is, however, more ambiguous for the femur (*p* = 0.054) and the fibula (*p* = 0.0806), and should be taken with more caution and confirmed with a larger sample. No significant difference between group allometries was found here for the forelimb bones. A significant difference in the allometric group intercepts was detected in the femur, and in the allometric group slopes were detected in the tibia and in the fibula. This result indicates that the allometric pattern between columnar‐limbed and non‐columnar‐limbed sauropodomorphs is different, and furthermore suggests that the evolutionary history of the sauropodomorph hindlimb bones is marked by an allometric shift. The visualization of allometric patterns, however, must be taken with caution compared to the PCs visualizations, as it represents a common allometric trend for both columnar and non‐columnar groups at the same time. Because groups with a lesser range of variation contribute less to the regression (Klingenberg, 2016), and since the number of specimens per group can be unbalanced, the estimation of the allometry‐linked shape variation can be driven to a slightly larger extent by the largest group. This situation would thus more reflect the allometric variation seen in this group than the general common pattern of both groups.

#### 4.3.1 Humerus

For the humerus, the major trend related to size is the global narrowing of the bone. Proximally, it seems to be associated in sauropods with the reduction of the medial tuberosity. In non‐sauropodan‐sauropodomorphs, it corresponds to a lowering of this tuberosity against the shaft, as seen in *Plateosaurus*. In the distal end, the narrowing of the condyles participates in the global narrowing of the bone. The trend can be more driven, in non‐sauropodan sauropodomorphs, by *Plateosaurus* (or Plateosauridae), since the larger specimens of the non‐sauropodan sauropodomorphs group belong to this genus. The deltopectoral crest is more centred transversally and more distally placed along the shaft. This placement results in better leverage of the muscles inserting on the crest (Carrano, 1999), involved primarily in forearm abduction and adduction (Otero, 2018). The olecranon fossa is deeper, which indicates an increase or at least a conservation of the extension abilities of the forearm. Our analysis finds that the shaft is more sigmoidal in larger specimens, which seems inconsistent with the straightening trend seen in sauropods, and more generally in dinosaurs (Carrano, 2000). It can mean that this observation is more influenced by the sigmoidicity observed in non‐sauropodan sauropodomorphs, which may increase with size. This observation, inconsistent with the rest of the analysis, bring us to take the result of the allometric pattern in the humerus with some caution.

#### 4.3.2 Forelimb zeugopod

In the radius, the proportional increase in robustness with size strongly recalls the morphologies of non‐sauropodan sauropodomorphs, as sauropod radii are more gracile. This is likely due to the fact that they are more numerous than sauropod radii in our analysis. Our results therefore probably do not reflect a common trend between non‐sauropodan sauropodomorphs and sauropods. Conversely, the visualization of the common allometric pattern of the ulna depicts variations relatively consistent with what is seen in the humerus. The proximal end is triradiate, but not clearly U‐shaped, and the distal end is anteroposteriorly reduced. The increasing robustness is, however, not consistent with patterns seen in the phylomorphospace. As for the radius, the trends here depicted may reflect more an allometric pattern in non‐sauropodan sauropodomorphs rather than in sauropods. This could also mean that the reduction of robustness and the other features seen only in sauropods, such as the characteristically U‐shaped proximal ulnar end, is not related to a common size‐related trend and is rather characterizing uniquely non‐sauropodan sauropodomorphs. This interpretation is consistent with the fact that the shape of the forelimb zeugopod of sauropods is diagnostic of the clade.

#### 4.3.3 Femur

For the femur, we observe some features classically associated with weight‐bearing, namely shaft straightening and distal displacement of the fourth trochanter. The shaft straightness is size‐related, corroborating the affirmation that this variation is more related to size increase rather than to posture (Carrano, 2001). The distal displacement of the fourth trochanter suggests the lengthening of the lever arm related to the *caudofemoralis longus*, allowing a greater extension (see above). The femoral head orientation slightly variates, suggesting that the strictly medial orientation seen in all sauropods is specific to this morphofunctional group within Sauropodomorpha (Carrano, 2000). On the proximal end, larger specimens tend to have a posteriorly developed area. In non‐sauropodan sauropodomorphs, it seems to correspond to the ‘medial tuber’ (Langer, 2003) or the ‘tuberosity’ laterally bounding the ligament of the femoral head (Novas, 1996). Novas (1996) noted that this feature is present in non‐sauropodan sauropodomorphs, and is developed in the large‐sized *Plateosaurus*. We generalize this observation as size‐related among non‐sauropodan sauropdomorphs. In sauropods, the sulcus of the ligament of the femoral head is indistinguishable from the rest of the head (Tsai et al., 2018). This might correspond to a convergent posterior development in larger taxa, probably optimizing support in both groups, but not with the same structure. On the distal end, the medial side tends to be more developed with increasing size compared to the other parts of the end. This medial reinforcement possibly increases abilities in weight‐bearing (Mallet et al., 2019).

#### 4.3.4 Hindlimb zeugopod

For the hindlimb zeugopod, the significant differences between the allometric trajectories of the non‐columnar sauropodomorphs and the columnar sauropods match with the stepwise interpretation of the pattern seen in the phylomorphospace. The antagonist trend of robustness is also observed. The greater the size of non‐sauropodomorphs, the more robust the hindlimb zeugopod, whereas it is the contrary in sauropods. An allometric shift has also been observed in various large‐sized mammals compared to smaller relatives (Bertram & Biewener, 1990). However, the proportional decrease in robustness is particularly surprising, as the global trend for large mammals is an increase in robustness (e.g. Bertram & Biewener, 1990; Christiansen, 2007; Mallet et al., 2019). This latter trend suggests that size increase was not associated with a proportional mass increase (see below). This may also be linked to the fact that the sampled taxon with the greater centroid size is *Diplodocus*, for which the body mass estimation is relatively low compared with other sauropods (Campione, 2017). If the sampled specimens for the tibia and the fibula attributed to *B. africanus* are correctly taxonomically assigned, the size relatedness of this observation would in fact correspond or interact with a peculiar allometric trend of a subgroup within Diplodocoidea (a large clade comprising notably *Diplodocus* and *Dicraeosaurus*) rather than be a trend general to Sauropoda. A larger sample of sauropods, including more specimens of large size from Diplodocoidea and other groups (e.g. non‐neosauropodan sauropods, Brachiosauridae, *Camarasaurus*) would permit us to clarify this observation. In any case, this would suggest that other morphoanatomical features were stronger than bone robustness to cope with an increase in size and, *a fortiori*, mass. Indeed, in sauropods, a relatively subtle shaft straightening is observed and seems not related to taphonomy, as seen in well‐preserved specimens of *Nigersaurus* (MNHN.F.GDF2094) and *Lapparentosaurus* (MNHN.F.MAA66). A similar straightening is retrieved in large mammals and non‐avian dinosaurs (Bertram & Biewener, 1990; Carrano, 2001). Some morphological features of the zeugopod remain well developed in large sauropods. In the tibia, this is the case of the lateral margin of the proximal end, contacting with the proximal end of the fibula by its rugose medial scar. This suggests a tighter association of the proximal ends in larger sauropods, somewhat similar to the tight association of the radius and ulna. Distally, the medial corner of the distal end is more developed. Again, this may represent a medial reinforcement in relation to a more efficient weight‐bearing (Mallet et al., 2019). Also, some variation of obliquity is observed in the proximal and distal ends of the tibia, and in the non‐sauropodan sauropodomorph fibular distal end, which seems correlated with robustness variation: the more robust the bone (in larger non‐sauropodan sauropodomorphs and in smaller sauropods), the more oblique these ends. The tibial distal end hence tends to be flatter in larger sauropods. This trend seems to be again observed in large Rhinocerotidae, jointly with an astragalus flattening (Etienne et al., 2020; Mallet et al., 2019). The astragalus is of particular importance in the limb architecture as the tibial and fibular distal ends are directly articulated with its proximal and lateral faces (Wilhite, 2003; Wilson & Sereno, 1998). A quantitative assessment of the shape variation of the sauropodomorph astragalus and of its link with size would complete our understanding of the evolutionary pattern occurring in the distal hindlimb during the emergence of sauropods.

### 4.4 Sketching out the morphofunctional limb diversity in sauropodomorph dinosaurs

#### 4.4.1 Weight‐bearing in sauropods

The peculiarities of the sauropod limbs fit largely with the classic definitions of ‘graviportality’ (Coombs, 1978; Gregory, 1912; Hildebrand, 1982; Osborn, 1929; Polly, 2007). Indeed, the fore and hindlimbs of sauropods are columnar, with elongated and straight limb bones and with more distally placed muscle attachments, conferring longer lever arms, and hence, more limb power per stride (Carrano, 1999). The sauropod forelimb is, however, proportionally very gracile compared to non‐sauropodan sauropodomorphs, with particularly reduced ends. The sauropod forelimb may have had a more or slightly more limited role in weight‐bearing than in quadrupedal mammals, such as elephants, where the forelimb has a greater support role than the hindlimb (Christiansen, 1997; Henderson, 2006). The functional comparison between sauropod and elephant forelimb is however not simple, since sauropods show a unique semi‐tubular arrangement of the manus, without any extant analogue. The weight support in most sauropods relied at least slightly more on the hindlimbs than on the forelimbs, with a more caudal centre of mass (Carrano, 2001; Henderson, 2006; Mallison, 2011) than in mammals (Henderson, 2006; Hildebrand, 1982). The presence of a fleshy pad only in the pes and not in the manus (Jannel et al., 2019; Wilson, 2005a) tends to support this assessment for weight distribution.

Our results, however, show a counter‐intuitive decrease in tibia and fibula robustness related to size increase among sauropods, especially in *Diplodocus* and some specimens attributed to *B. africanus*. This suggests that some morphological traits are more important in locomotion and weight‐bearing than the sole robustness metric. Columnarity, and bone shape features, such as the development, or absence of reduction, of the medial part of the hindlimb bones when size increases, may have been sufficient for weight‐bearing in sauropods. The decrease of the tibial obliquity and curvature may have also been important, allowing a more aligned hindlimb, probably more efficient in weight‐bearing. The more developed lateral part of the tibial proximal end, together with the characteristic scar seen in the fibular medial proximal end, may have permitted a tighter association of the two ends, and *a fortiori*, of the two bones. In a different way, this could also indicate that mass increase in sauropods was relatively less important than size increase. Indeed, mass increases proportionally to the cube of length increase for geometrically similar bones (e.g. Alexander, 1998). But if robustness proportionally decreases with size increase, the mass increase should be consequently diminished. This hypothesis is consistent with the post‐cranial pneumatization observed across Sauropodomorpha, and particularly in sauropods (Wedel, 2003, 2005; Yates et al., 2012), which participate to lower the body mass estimations inferred for sauropods (Wedel, 2005).

#### 4.4.2 Locomotion in sauropods

Sauropod and non‐sauropodan sauropodomorph dinosaurs show markedly different limb bones, which directly impacts the morphofunctional inferences related to their locomotion. Sauropod obligate quadrupedal locomotion seems to principally rely on protraction and especially retraction of the stylopodial elements (Carrano, 2005; Christiansen, 2007). Our results corroborate this statement, with notably a more distally placed deltopectoral crest, and a prominent humeral posterolateral ridge, conferring longer stride length. The fourth trochanter, although reduced, is more distally placed. The retraction capabilities of the hindlimb may have been still of major importance in sauropod locomotion, given the still largely developed condition of sauropod tails (Díez et al., 2020). Moreover, the anteriorly and posteriorly developed ilium of sauropods indicates that protraction and retraction were important in this clade (Carrano, 2000, 2005). Despite the apparent reduction of the olecranon process and cnemial crest, which may have decreased the extension abilities of the sauropod limbs (Carrano, 2005), our results show that extensors muscles may have been still powerful in sauropods, presenting a deepened olecranon fossa and a somewhat anterolaterally developed and more distally placed cnemial crest. This statement for the latter structure is corroborated by a recent biomechanical study (Klinkhamer et al., 2018) highlighting larger moment arms in knee extension (and flexion) in sauropod taxa than in the non‐sauropodan sauropodomorph *Plateosaurus*. Such extension capacities may have been paramount to keep the limb in a columnar position, counteracting against gravity that tends to flex the limbs (Henderson et al., 2017; Milne, 2016). In the forelimb, Milne (2016) and Henderson et al. (2017) showed that quadrupedal terrestrial mammals present a posterior curvature in the ulna, counteracting the habitual stress of the *triceps* extensor action. This statement is also observed in our analyses of sauropods and corroborates our interpretation of a somewhat powerfully extended sauropod elbow. The sauropod manus is mainly dedicated to body support, with a phalangeal reduction and a characteristic arrangement, from relatively open to semi‐tubular (Allain & Aquesbi, 2008; Wilson & Sereno, 1998), possibly corresponding to the observation of coalescent and tightly associated radial and ulnar distal ends (Hutson, 2015; Wilhite, 2003). The locomotor role of the forelimb was probably less important than that of the hindlimb, which was the principal actor of forward propulsion (Christiansen, 1997; Díez et al., 2020), based on the retraction of tail muscles, notably the *caudofemoralis longus* (Díez et al., 2020; Gatesy, 1990, 1995).

#### 4.4.3 Comparison with the non‐sauropodan sauropodomorphs

Comparatively, non‐sauropodan sauropodomorphs’ limbs show an overall pattern markedly different: it is globally more robust, with developed proximal and distal ends. In the humerus, the olecranon fossa is markedly less developed, whereas a cuboid fossa is present, allowing more flexion than in sauropods (Ballell et al., 2022; Bonnan, 2003; Bonnan & Senter, 2007). The proximal end of the ulna is bulky, with no to a poorly developed radial fossa, compared to sauropods. Added to a very limited contact of the distal part of the ulna and of the radius (see Apaldetti et al., 2018; Otero et al., 2017), the association of these two bones is very loose in non‐sauropodan sauropodmorphs (Otero et al., 2017), compared to sauropods (Wilhite, 2003). Moreover, although a slight posterior curvature exists, the ulna is also particularly medially bent. This medial bending seems not to be as efficient as the condition of terrestrial quadrupedal mammals in order to counteract the action of the *triceps* (see Milne, 2016). The medially straighter condition of the sauropod ulna, with its posterior bending, seems to be similar to the condition of terrestrial quadrupedal mammals. Also, in non‐sauropodan sauropodomorphs, the manus displays more numerous phalanges, with a characteristic first digit, interpreted to be able of grasping (Galton & Upchurch, 2004; Otero et al., 2017), and hence, that is probably not confined to an exclusive role of locomotion and weight‐bearing, conversely to in sauropods. These observations, although not totally precluding inferences of quadrupedality in non‐sauropodan sauropodomorphs, tend to question its efficiency as the principal mode of locomotion for these taxa. Indeed, given that the shape of the antebrachium bones of sauropods is drastically different from that of non‐sauropodan sauropodomorphs, the quadrupedal locomotor abilities of the latter group may be less performant than those of the sauropods, in the context of quadrupedality as the main mode of locomotion.

In the hindlimb, the gap between the non‐sauropodan sauropodomorphs and the sauropods is less marked. Morphologically, for the femur, several features characterizing sauropods are found separately in some non‐sauropodan sauropodomorphs, suggesting a substantial part of homoplasticity in the sauropod femur. The evolution of the sauropod hindlimb zeugopod seems to be a stepwise process, involving continuous variation of features, such as robustness, and punctual episodes of strong changes, notably related to the emergence of the sauropod bauplan. Non‐sauropodan sauropodomorphs tend to display morphological features traditionally associated with a pattern implying faster locomotion, notably the proximal position of the cnemial crest, conferring smaller lever arms (Carrano, 1999; Klinkhamer et al., 2018) and possibly affecting the range of knee flexion (Ballell et al., 2022). More putatively, the *iliofibularis* muscle, involved in knee flexion (Carrano, 2000), is also found more proximally along the fibular shaft. The fourth trochanter is also generally proximally placed, though also found more distally placed in some non‐sauropodan sauropodiforms (see also Ballell et al., 2022; Yates et al., 2010). These features tend to separate the locomotor capabilities seen in non‐sauropodan sauropodomorphs, more oriented towards an efficient fast bipedal locomotion, from the sauropod profile, with more traits favouring efficient weight‐bearing rather than fast locomotion. This is congruent with the differential allometric patterns seen between these two locomotor groups. While large non‐sauropodan sauropodomorphs tend to present more robust tibia and fibula, large sauropods present bone shape variations (e.g. medial reinforcements, flattening of the tibial distal end) and a surprising decrease in tibial and fibular robustness. The distribution of this latter trend may need to be clarified within sauropods.

The synthesis of the features highlighted in this study (Table 1) suggests a differential evolutionary history for the fore and hindlimbs associated with the emergence of sauropods.

**TABLE 1:** Summarized table of the main features discussed morphofunctionally in this study

Location	Bone	Feature	State distribution	Inferred implication
FL and HL		Proximodistal position of muscle insertions	More distally placed deltopectoral crest (SR), fourth trochanter (SR), sauropod cnemial crest (SR)	Stronger lever arms, more powerful movements for less velocity
		Reduction of stylopod limb muscle insertions	Reduced deltopectoral crest and femoral trochanters in SPD	Possible diminution of the limb movement repertoire in SPD (but see details in text)
		Limb Torsion	Reduced in SPD for humerus, radius, ulna, femur, fibula	Possible locomotor shifts of muscles related to subsequent origin/insertion orientation shifts
		Shaft straightening	SPD humerus, ulna, a tibia (SR), femur in all taxa (SR)	Reduces bone stresses related with locomotion and weight‐bearing
FL	FL	Forelimb robustness	Slender in SPD, considerably more robust in NSS	Probable lesser weight‐bearing capacity than hindlimb
	H	Humeral medial tuberosity	Reduced and pinched in SPD	Reduction of adduction and humeral pronation
		Humeral proximolateral region	Sharply bent in SPD, associated with a marked posterolateral ridge	Substantial ability in extension and supination
		Humeral olecranon fossa	Deep in SPD, shallow in NSS; deeper in larger taxa (SR)	Increase (SR)/conservation (SPD) of efficient elbow extension
		Humeral cuboid fossa	Absence in SPD, presence in NSS	Diminution of flexion in SPD
	U	Ulnar olecranon process	Reduced in SPD (but see olecranon fossa)	Diminution of elbow extension
		Ulnar proximal end	Clearly triradiate and U‐shaped in SPD, variably different in NSS (see text); triradiate in larger taxa (SR)	More efficient body support and locomotion in SPD
		Ulnar radial fossa	Deep in SPD, variably shallow in NSS; deeper in larger taxa (SR)	
		Ulnar shaft	Almost straight and slightly posteriorly curved in SPD; curved medially in NSS	Counteract stress occasioned by *triceps* muscles in SPD
	R and U	Radius and ulna distal ends	Reduced and more tightly associated in most SPD	More efficient body support
HL	HL	Medial reinforcement	Femoral medial condyle (SR), tibial proximal end (SR)	Potentially increases weight‐bearing
	Fm	Femoral head	Convergent posterior development (SR)	Possible more efficient weight‐bearing
		Femoral lesser trochanter	More laterally placed but reduced in SPD	Substantial abduction/adduction abilities
		Femoral eccentricity	More important in most SPD and some NSS	Variation related to skeletal bending resistance
	T	Tibial proximal end	Less expanded, oblique and more circular in SPD (SR)	Better configuration for weight‐bearing (or less important mass increase; see text)
		Tibia, cnemial crest	In SPD, reduced width (see text), crest below the proximal end level	Substantial extension abilities in SPD
		Tibial proximal and distal ends obliquity	More oblique in larger NSS (SR), less oblique in larger SPD (SR)	More efficient weight‐bearing in SPD
	T and Fb	Tibia and fibula, shaft robustness	More robust in NSS, probably less robust in SPD (SR)	Better configuration for weight‐bearing (or less important mass increase; see text)
		Tibia and fibula, proximal end	In SPD only, increase of development of contacting area	Probable tighter association and better weight‐bearing

Abbreviations: Fb, fibula; FL, forelimb; Fm, femur; H, humerus; HL, hindlimb; NSS, non‐sauropodan sauropodomorphs; R, radius; SPD, Sauropods; SR, size‐related (feature visualized in the allometry analysis, some of them are also seen in the principal component analysis); T, tibia; U, Ulna.

The evolutionary scenario highlighted in our analysis tends to reject the hypothesis involving a progressive evolution of the sauropod forelimb (Bonnan & Yates, 2007; Yates et al., 2010). Instead, the forelimb bones of non‐sauropodan sauropodmorphs, especially the antebrachium ones, are markedly different from those of the sauropods, questioning their efficiency in the context of an inferred quadrupedality. Conversely, the morphological features of the hindlimb mostly evolved in a continuous way, with features already present in closely related non‐sauropodan sauropodomorphs, such as the distal placement of the fourth trochanter, and traits marking the emergence of sauropods, such as the particular condition of the cnemial crest, or the shift of allometric pattern in the tibia and fibula. This suggests a shift from bones allowing fast bipedal locomotion to bones showing more features in relation to weight‐bearing. Some other features appear in more nested groups of sauropods and, hence, are not directly associated with the emergence of the columnar sauropods, such as the distal displacement of the cnemial crest.

## 5 CONCLUSION AND PERSPECTIVES

The present study is the first investigation of the emergence of the sauropod bauplan focusing on the six limb long bones. Using 3D Geometric Morphometrics with curves and surface sliding semilandmarks, we were able to quantitatively analyze the detailed shape changes occurring during this evolutionary episode and to investigate their relation with size. Our results permitted us to highlight that the limb long bones of the columnar sauropods were different from those of non‐columnar non‐sauropodan sauropodomorphs. This distinction is based on numerous shape differences, including several morphological features not or poorly highlighted before, such as a deep olecranon fossa, or a particular configuration of the proximolateral area of the humerus. The evolution of the sauropod limb bauplan is not uniform: while the emergence of the sauropod forelimb zeugopod is abrupt, the pattern for the hindlimb zeugopod appears to be more gradual. The stylopod bones seem to follow the same evolutionary scenario seen in their respective zeugopods. Also, the allometric patterns seen for the six bones are not uniform. While the hypothesis of a similar allometric pattern is not rejected for the forelimb, an allometric shift episode was detected in the hindlimb, so that the hindlimb zeugopod allometry is different in the non‐sauropodan sauropodomorphs and in the sauropods. This is notably materialized by an increase of robustness in large non‐sauropodan sauropodomorphs, while a different pattern is seen in large sauropods, including a surprising decrease in robustness. Sauropods may have then relied more on the columnar architecture of their limb, and on other shape particularities related to the increase of size, which may have been sufficient for weight‐bearing, rather than on an increase in robustness. This observation is congruent with the assumption that the increase in mass in relation to the increase in size may have also been proportionally smaller than expected in sauropods. We conclude with the differential nature of the emergence of the sauropod bauplan between the forelimb and the hindlimb. Our study tends to show that the sauropod forelimb, especially the antebrachium, is particularly different from the non‐sauropodan sauropodomorph one. Even if it does not preclude a possibility of quadrupedality in the latter group, it seriously questions the efficiency of such a locomotor habit performed on a regular basis. Consequently, our study does not favour the hypothesis of a mosaic emergence at the scale of the forelimb taken alone. Conversely, the emergence of the sauropod hindlimb seems to be more gradual, starting from a non‐sauropodan sauropodomorph hindlimb more efficient in fast locomotion to a hindlimb more dedicated to efficient body support, and punctuated by an episode of important morphological and allometric change. Thus, the mosaic emergence of the sauropod limb bauplan only appears when both forelimb and hindlimb bones are taken into account, suggesting that a complex mixture of evolutionary rates occurred during this evolutionary episode.

The emergence of the sauropod bauplan is the starting point of the diversification of this clade. Further investigations on other episodes of the limb evolution in Sauropodomorpha (e.g. within sauropods) should expand our knowledge of the locomotor diversity occurring within this clade. In a larger context, sauropods are not the only vertebrates showing columnar limbs. Investigation of a larger number of groups showing large body size and mass, with or without columnar limbs, should refine our knowledge of the impact of this peculiar architecture, helping us in understanding how such extreme gigantism has been reached.

## AUTHOR CONTRIBUTIONS

Rémi Lefebvre, Alexandra Houssaye, Ronan Allain, Raphaël Cornette designed the study. Rémi Lefebvre and Heinrich Mallison acquired the data. Rémi Lefebvre performed the analyses with the help of Raphaël Cornette. Rémi Lefebvre, Alexandra Houssaye, Ronan Allain, Raphaël Cornette analyzed and interpreted the results. Rémi Lefebvre drafted the manuscript. All authors revised and approved the manuscript.

## Supporting information


Data S1

## Data Availability

Most of the data that support the findings of this study are or will be made available in respective museum repositories and/or by curators, and unless otherwise decided, deposited in Morphosource.
