# Supplementary material for: A path to gigantism: Three‐dimensional study of the sauropodomorph limb long bone shape variation in the context of the emergence of the sauropod bauplan
Source: J Anat. 2022 Mar 6;241(2):297–336. doi: 10.1111/joa.13646 (PMC9296025; doi:10.1111/joa.13646)
Supplement: Supplementary file 1 — Data S1 [file JOA-241-297-s001.docx]

# Supplementary Material

**Table S1**

**Material sampled in this study**

Institutional abbreviations: BP, Evolutionary Studies Institute (formerly Bernard Price Institute), University of the Witwatersrand, Johannesburg, South Africa; GPIT Institute for Geosciences, Eberhard-Karls-Universität Tübingen, Tübingen, Germany; IVPP, Institute of Vertebrate Paleontology and Paleoanthropology, Beijing, China; MACN, Museo Argentino de Ciencias Naturales Bernardino Rivadavia, Buenos Aires, Argentina; MB.R Museum für Naturkunde, Berlin, Germany; MHNM Muséum d’Histoire naturelle, Marrakech, Morroco; MLP, Museo de La Plata, La Plata, Argentina; MNHN, Muséum National d’Histoire Naturelle, Paris, France; PVL, Instituto Miguel Lillo, Universidad Nacional de Tucumán, San Miguel de Tucumán, Argentina; PVSJ, Instituto y Museo de Ciencias Naturales, Universidad Nacional de San Juan, San Juan, Argentina; SAM, Iziko South African Museum, Cape Town, South Africa; SMNS Staatliches Museum für Naturkunde, Stuttgart, Germany; UFSM, Universidade Federal de Santa Maria, Santa Maria, Rio Grande do Sul, Brazil.

Other abbreviations:

Ori.: Orientation: Left (L) or Right (R)

Dig.: Digitization technique employed: CT-Scan (CT); Photogrammetry (P); Surface Scanner (SS)

Cp: Qualitative evaluation of the completeness of the anatomical landmarks (Lk) and curves (Cu) locations: A: The incompleteness does not alter the placement of anatomical landmarks / the digitization of the outlines; B: The incompleteness alters softly the placement of anatomical landmarks / the digitization of the outlines; C: The incompleteness alters moderately the placement of anatomical landmarks / the digitization of the outlines

**Humerus**

| Taxon | Collection number | Ori. | Dig. | Cp Lk | Cp Cu | Comments |
| --- | --- | --- | --- | --- | --- | --- |
| *Adeopapposaurus mognai* | PVSJ 610 | L | SS | A | A | Small missing part on the shaft of the anteromedial virtually reconstructed (estimation very well constrained by surrounding bone shape) |
| *“Barosaurus africanus”* | MB.R.2709 | R | P | A | B | Minor hole on the 3D model virtually filled |
| *“Barosaurus africanus”* | MB.R.2639 | R | P | A | B |  |
| *Coloradisaurus brevis* | PVL 5904 | R | SS | A | B |  |
| *Dicraeosaurus sp.* | MB.R.2655 | L | P | A | C | Minor hole on the 3D model virtually filled |
| *Diplodocus sp.* | GPIT-PV-31370 | L | P | B | C |  |
| *Giraffatitan brancai* | MB.R.2658 | L | P | A | C |  |
| *Giraffatitan brancai* | MB.R.2682 | L | P | B | C |  |
| *Lapparentosaurus madagascariensis* | MNHN.F.MAA164 | R | SS | A | A |  |
| *Lapparentosaurus madagascariensis* | MNHN.F.MAA160 | R | SS | B | C |  |
| *Lufengosaurus huenei* | IVPP V15 | L | P | A | B | Mounted skeleton; missing part on the 3D model hidden by the structure virtually filled |
| *Massospondylus sp.* | BP/1/4860 | L | P | A | A |  |
| *Nigersaurus taqueti* | MNHN.F.GDF2097 | L | SS | A | A |  |
| *Plateosauravus cullingworthi* | SAM-PK-3342 | L | P | A | B |  |
| *Plateosaurus sp.* | SMNS 91310 (F65d512) | R | SS | A | B |  |
| *Plateosaurus sp.* | SMNS 80664 | L | SS | A | A |  |
| *Plateosaurus sp.* | SMNS 12949 | L | SS | B | B |  |
| *Plateosaurus sp.* | SMNS 91296 (F10) | L | SS | A | A |  |
| *Tornieria africana* | MB.R.2673 | L | P | A | B |  |
| *Unaysaurus tolentinoi* | UFSM 11069 | R |  | B | C |  |

Other species considered but not used in the quantitative analysis due to incompleteness and/or deformations:

*Amargasaurus cazui, Antetonitrus ingenipes,* “*Bothriospondylus madagascariensis”,* BP/1/7811, *Cetiosauriscus stewarti Efraasia minor, “Euskelosaurus browni”, Kholumolumo ellenbergerorum, Lessemsaurus sauropoides*, *Ingentia prima, Melanorosaurus sp.,* MNHN.F.LES400*, Mussaurus patagonicus, Patagosaurus fariasi, Riojasaurus incertus, Sanpasaurus yaoi, Sefapanosaurus zastronensis, Tazoudasaurus naimi, Vouivria damparisiensis*

**Radius**

| Taxon | Collection number | Ori. | Dig. | Cp Lk | Cp Cu | Comments |
| --- | --- | --- | --- | --- | --- | --- |
| *Aardonyx celestae* | BP/1/5379 | L | P | B | C |  |
| *Aardonyx celestae* | BP/1/6321 | L | P | A | B |  |
| *Adeopapposaurus mognai* | PVSJ610 | R | SS | B | B |  |
| *Antetonitrus ingenipes* | BP/1/4952 | R | P | C | C |  |
| *Dicraeosaurus sp.* | MB.R.2622 | L | P | B | B |  |
| *Diplodocus sp.* | GPIT-PV-31370 | L | P | A | B |  |
| “Ha Noosi Prosauropod” | MNHN.F.LES400 | L | SS | B | C | Piece of matrix removed and resulting hole refilled virtually |
| *Lapparentosaurus madagascariensis* | MNHN.F.MAA 32 | L | SS | A | A |  |
| *Massospondylus sp.* | SAM-PK-391 | R | P | B | B |  |
| *Melanorosaurus readi* | SAM-PK-3449 | R | P | B | B |  |
| *Melanorosaurus sp.* | BP/1/5090 | L | P | B | B | Virtually merged |
| *Patagosaurus fariasi* | MACN-CH-932 | R | SS | B | B |  |
| *Plateosauravus cullingwothi* | SAM-PK-3347 | L | P | A | A |  |
| *Plateosaurus sp.* | SMNS 91296 (F10) | L | SS | A | B |  |
| *Plateosaurus sp.* | SMNS 91296 (F10) | L | SS | A | B |  |
| *Plateosaurus sp.* | GPIT II | L | CT | B | C |  |
| *Plateosaurus sp.* | SMNS 12949 | L | SS | A | B |  |
| *Plateosaurus sp.* | SMNS 91310 (F65) | R | SS | A | B |  |
| Sauropoda indet. | MB.R.2607 | L | P | B | C |  |
| *Sefapanosaurus zastronensis* | BP/1/7435 | R | P | C | C | Virtually merged |
| *Sefapanosaurus zastronensis* | BP/1/7436 | R | P | B | C |  |

Other species considered but not used in the quantitative analysis due to incompleteness and/or deformations:

*Amargasaurus cazui, Efraasia minor,* *Eucnemesaurus sp., Kholumolumo ellenbergerorum, Lessemsaurus sauropoides*, *Ingentia prima,* MNHN.F.LES400*, Mussaurus patagonicus, Riojasaurus incertus, Ruehleia bedheimensis, Sanpasaurus yaoi, Tazoudasaurus naimi, Unaysaurus tolentinoi*

**Ulna**

| Taxon | Collection number | Ori. | Dig. | Cp Lk | Cp Cu | Comments |
| --- | --- | --- | --- | --- | --- | --- |
| *Adeopapposaurus mognai* | PVSJ610 | R | SS | A | A |  |
| *Antetonitrus ingenipes* | BP/1/4952 | R | P | B | B |  |
| *Antetonitrus ingenipes* | BP/1/4952 | R | P | B | B |  |
| *“Barosaurus africanus”* | MB.R.2604 | L | P | A | A |  |
| *“Barosaurus africanus”* | MB.R.2602 | R | P | A | B |  |
| *Dicraeosaurus sp.* | MB.R.2601 | R | P | A | B |  |
| *Diplodocus sp.* | GPIT-PV-31370 | L | P | A | A |  |
| *Kholumolumo ellenbergerorum* | MNHN.F.LES159 | R | SS | B | B |  |
| *Lapparentosaurus madagascariensis* | MNHN.F.MAA31 | L | SS | A | B |  |
| *Lapparentosaurus madagascariensis* | MNHN.F.MAA162 | R | SS | A | A |  |
| *Ledumahadi mafube* | BP/1/5339 | R | P | B | B |  |
| *Lessemsaurus sauropoides* | PVL 4822/54 | L | SS | A | A | Small missing part of the projection on the shaft of the anteromedial process virtually reconstructed (estimation very well constrained by surrounding bone shape) |
| *Massospondylus sp.* | BP/1/4860 | L | P | A | A |  |
| *Massospondyus sp.* | MNHN.F.LES226 | L | SS | B | B |  |
| *Melanorosaurus readi* | SAM-PK-3449 | R | P | A | B |  |
| *Mussaurus patagonicus* | MLP 68-II-27-1 | R | SS | B | B |  |
| *Nigersaurus taqueti* | MNHN.GDF.242.3 | R | SS | B | B |  |
| *Patagosaurus fariasi* | MACN-CH-932 | R | SS | A | B |  |
| *Plateosauravus cullingworthi* | SAM-PK-3351 | L | P | A | A |  |
| *Plateosaurus sp* | SMNS 91306 (F48) | L | SS | A | A |  |
| *Plateosaurus sp* | GPIT II | L | CT | A | A |  |
| *Plateosaurus sp* | SMNS 91296 (F10) | R | SS | A | B |  |
| *Plateosaurus sp* | SMNS 91310 (F65) | R | SS | B | B |  |
| *Plateosaurus sp.* | SMNS 12950 | R | SS | A | A |  |
| *Plateosaurus sp.* | SMNS 13200 | R | SS | A | A |  |
| *Riojasaurus incertus* | PVL 3808 | R | SS | B | C |  |
| *Ruehleia bedheimensis* | MB.R.4718.58 | R | P | A | A |  |
| *Sefapanosaurus zastronensis* | BP/1/7437 | L | P | A | A |  |
| *Tornieria africana* | MB.R.2586 | R | P | A | B |  |

Other species considered but not used in the quantitative analysis due to incompleteness and/or deformations:

*Aardonyx celestae, Amargasaurus cazui, “Botrhiospondylus madagascariensis”, Efraasia minor,* *Eucnemesaurus sp., Giraffatitan brancai, “Gryponyx africanus”, Ingentia prima,* MNHN.F.LES400*, Pulanesaura eocollum, Sanpasaurus yaoi, Tazoudasaurus naimi, Unaysaurus tolentinoi, Vouivria damparisiensis*

**Femur**

| Taxon | Collection number | Ori. | Dig. | Cp Lk | Cp Cu | Comments |
| --- | --- | --- | --- | --- | --- | --- |
| *Adeopapposaurus mognai* | PVSJ569 | L | SS | B | B |  |
| *Adeopapposaurus mognai* | PVSJ610 | L | SS | B | B |  |
| *“Barosaurus africanus”* | MB.R.2637 | L | P | B | B | Minor holes on the 3D model virtually filled |
| *“Barosaurus africanus”* | MB.R.2641 | L | P | A | A |  |
| *“Barosaurus africanus”* | MB.R.2662 | L | P | A | B |  |
| *Coloradisaurus brevis* | PVL 5904 | R | SS | A | A |  |
| *Giraffatitan brancai* | MB.R.HMN SII | R | P | A | B | Mounted skeleton; missing part on the 3D model hidden by the structure virtually filled |
| *Giraffatitan brancai* | MB.R.2633 | R | P | B | B |  |
| *“Gyposaurus sinensis”* | IVPP V26-10 | R | P | A | B |  |
| *Kholumolumo ellenbergerorum* | MNHN.F.LES371 | L | SS | B | B |  |
| *Lapparentosaurus madagascariensis* | MNHN.F.MAA167 | L | SS | B | B | Moderately important reconstruction of the lateral side of the shaft under the fourth trochanter (estimation very well constrained by surrounding bone shape) |
| *Lapparentosaurus madagascariensis* | MNHN.F.MAA166 | R | SS | A | A |  |
| *Lessemsaurus sauropoides* | PVL 4822/65 | R | SS | C | C |  |
| *Massospondylus carinatus* | BP/1/4266 | R | P | A | B |  |
| *Meroktenos thabanensis* | MNHN.F.LES16c | R | SS | C | C |  |
| *Nigersaurus taqueti* | MNHN.F.GDF75 | R | SS | C* | C* | Landmarks and curve for the 4^th^ trochanter estimated on the basis of the extension of the *caudofemoralis longus* muscle insertion. |
| *Plateosaurus sp.* | SMNS 91306 (F48) | R | SS | C | C |  |
| *Plateosaurus sp.* | GPIT I | R | CT | B | C |  |
| *Plateosaurus sp.* | SMNS 13200 | R | SS | A | A |  |
| *Riojasaurus incertus* | PVL 3662 | L | SS | A | A |  |
| *Ruehleia bedheimensis* | MB.R.4718.98 | L | P | A | A | Minor hole on the 3D model virtually filled; small reconstruction of the medial side of the shaft under the femoral head (estimation very well constrained by surrounding bone shape) |
| *Ruehleia bedheimensis* | MB.R.4753 | R | P | A | B |  |

Other species considered but not used in the quantitative analysis due to incompleteness and/or deformations:

*Aardonyx celestae, Amargasaurus cazui, Amargatitanis macni Antetonitrus ingenipes, “Botrhiospondylus madagascariensis”,* BP/1/7811, *Cetiosauriscus stewarti, Chromogisaurus novasi, Diplodocus sp., Dicraeosaurus sp. Efraasia minor,* *Eucnemesaurus sp., “Euskelosaurus browni”, “Gryponyx africanus”, Ledumahadi mafube, Melanorosaurus sp.,* MNHN.F.LES400*, Mussaurus patagonicus, Patagosaurus fariasi, Sanpasaurus yaoi, Tazoudasaurus naimi,“Thecodontosaurus sp.”, Volkheimeria chubutensis, Vouivria damparisiensis*

**Tibia**

| Taxon | Collection number | Ori. | Dig. | Cp Lk | Cp Cu | Comments |
| --- | --- | --- | --- | --- | --- | --- |
| *Adeopapposaurus mognai* | PVSJ 569 | L | SS | A | B |  |
| *Adeopapposaurus mognai* | PVSJ 610 | R | SS | B | A |  |
| *Antetonitrus ingenipes* | BP/1/4952 | L | P | B | C |  |
| *“Barosaurus africanus”* | MB.R.2597 | L | P | A | B | Minor hole on the 3D model virtually filled |
| *“Barosaurus africanus”* | MB.R.2599 | R | P | A | A |  |
| *Coloradisaurus brevis* | PVL 5904 | R | SS | A | A |  |
| *Diplodocus carnegii* | MNHN.F.1908.18 | R | P | A | C | Mounted skeleton; missing part on the 3D model hidden by the structure virtually filled |
| *Diplodocus sp.* | GPIT-PV-31370 | R | P | A | B |  |
| *Giraffatitan brancai* | MB.R.2676 | R | P | B | B |  |
| *Lapparentosaurus madagascariensis* | MNHN.F.MAA66 | L | SS | A | A |  |
| *Massospondylus sp.* | BP/1/5006 | L | P | A | A | Virtually merged |
| *Massospondylus sp.* | SAM-PK-K391 | R | P | B | B |  |
| *Melanorosaurus sp.* | BP/1/5090 | L | P | C | C | Virtually merged |
| *Mussaurus patagonicus* | MLP 68-II-27-1 | R | SS | B | B |  |
| *Nigersaurus taqueti* | MNHN GDF | L | SS | A | A |  |
| *Nigersaurus taqueti* | MNHN GDF 2047 | R | SS | B | C | Virtually merged, missing part of the shaft reconstructed (estimation very well constrained by surrounding bone shape) |
| *Panphagia protos* | PVSJ 874 | R | SS | A | A |  |
| *Plateosauravus cullingworthi* | SAM-PK-3341 | R | P | A | A |  |
| *Plateosaurus sp.* | SMNS F48 (91306) | L | SS | B | B |  |
| *Plateosaurus sp.* | SMNS 91310 (F65) | R | SS | C | C |  |
| *Plateosaurus sp.* | GPIT I | R | CT | A | A |  |
| *Plateosaurus sp.* | SMNS 13200 | R | SS | A | A |  |
| *Riojasaurus incertus* | PVL 3808 | L | SS | B | C |  |
| *Tornieria africana* | MB.R.2572 | L | P | A | A | Virtually merged |
| *Volkheimeria chubutensis* | PVL 4077 | L | SS | B | C | Virtually merged, small part of the cnemial crest reconstructed (estimation very well constrained by surrounding bone shape) |

Other species considered but not used in the quantitative analysis due to incompleteness and/or deformations:

*Amargasaurus cazui, Blikanasaurus cromptoni, “Botrhiospondylus madagascariensis”,* BP/1/7811, *Cetiosauriscus stewarti, Cetiosaurus oxoniensis, Chromogisaurus novasi, Dicraeosaurus sp., Efraasia minor,* *Eucnemesaurus sp., “Euskelosaurus browni”, Giraffatitan brancai, “Gryponyx africanus”, “Gyposaurus sinensis”, Kholumolumo ellenbergerorum, Lessemsaurus sauropoides,* MNHN.F.LES400*, Mussaurus patagonicus, Patagosaurus fariasi, Pulanesaura eocollum, Sanpasaurus yaoi, Tazoudasaurus naimi, “Thecodontosaurus sp.”, Vouivria damparisisensis*

**Fibula**

| Taxon | Collection number | Ori. | Dig. | Cp Lk | Cp Cu | Comments |
| --- | --- | --- | --- | --- | --- | --- |
| *Adeopapposaurus mognai* | PVSJ 569 | R | SS | B | / |  |
| *Antetonitrus ingenipes* | BP/1/4952 | L | P | C | / |  |
| *“Barosaurus africanus”* | MB.R.2616 | L | P | A | / |  |
| *“Barosaurus africanus”* | MB.R.2623 | L | P | C | / |  |
| *Dicraeosaurus sp.* | MB.R.2628 | R | P | A | / | Missing part of the shaft reconstructed (estimation very well constrained by surrounding bone shape); small hole on the mesh virtually filled |
| *Dicraeosaurus sp.* | MB.R.2618 | L | P | C | / |  |
| *Diplodocus carnegii* | MNHN.F.1908.18 | R | P | C | / | Mounted skeleton; missing part on the 3D model hidden by the structure virtually filled |
| *Massospondylus sp.* | SAM-PK-K394 | L | P | A | / |  |
| *Nigersaurus taqueti* | MNHN GDF 2055 | R | SS | C | / | Virtually merged |
| *Nigersaurus taqueti* | MNHN GDF 2095 | R | SS | A | / |  |
| *Plateosaurus sp.* | SMNS F48 (91306) | L | SS | A | / |  |
| *Plateosaurus sp.* | GPIT uncat. | L | SS | A | / |  |
| *Plateosaurus sp.* | SMNS F14 (91297) | R | SS | B | / | Virtually merged |
| *Plateosaurus sp.* | GPIT I | R | CT | B | / |  |
| *Plateosaurus sp.* | GPIT uncat. | R | SS | A | / |  |
| *Plateosaurus sp.* | SMNS 13200 | R | SS | B | / |  |
| *Plateosaurus sp.* | SMNS 13200a+e | R | SS | B | / |  |
| *Riojasaurus incertus* | PVL 3808 | L | P | B | / | Virtually merged |
| *Riojasaurus incertus* | PVL 3663 | R | SS | A | / |  |
| Sauropoda indet. | MB.R.2588 | L | P | B | / |  |
| *Sefapanosaurus zastronensis* | BP/1/7447 | L | P | B | / |  |
| *Tazoudasaurus naimi* | MHNM To1-637 | L | SS | B | / |  |
| *Tazoudasaurus naimi* | MHNM To1-377 | L | SS | C | / |  |
| *Tornieria africana* | SMNS 12142 | R | SS | C | / | Virtually merged |

Other species considered but not used in the quantitative analysis due to incompleteness and/or deformations:

*Aardonyx celestae, Amargasaurus cazui, Blikanasaurus cromptoni, “Botrhiospondylus madagascariensis”,* BP/1/7811, *Cetiosauriscus stewarti, Coloradisaurus brevis, Efraasia minor,* *Giraffatitan brancai, “Gryponyx africanus”, “Gyposaurus sinensis”, Kholumolumo ellenbergerorum, Ingentia prima, Lessemsaurus sauropoides,* MNHN.F.LES400*, Mussaurus patagonicus, Patagosaurus fariasi, Sanpasaurus yaoi, Vouivria damparisisensis*

**Table S2**

**List of landmarks in this study**

**Number of landmarks per bone**

|  | Anatomical landmarks | Curve sliding semiladmarks | Surface sliding semilandmarks | Total |
| --- | --- | --- | --- | --- |
| Humerus | 7 | 164 | 396 | 567 |
| Radius | 3 | 91 | 407 | 501 |
| Ulna | 6 | 126 | 310 | 442 |
| Femur | 14 | 116 | 470 | 600 |
| Tibia | 8 | 124 | 382 | 514 |
| Fibula | 8 | 0 | 494 | 502 |

**Definition and location of landmarks in each type of bone**

For each type of bone, anatomical landmark definitions are here provided, and anatomical landmarks (red spheres), curve sliding (blue spheres) and surface sliding (green spheres) semilandmarks locations are illustrated:

**Humerus**

| # | Anatomical Landmark Definition |
| --- | --- |
| 1 | Maximum of concavity between the lateral tubercle and the humeral head outline in the delimitation between the proximal end and the shaft |
| 2 | Most caudal point in proximal view of the humeral head |
| 3 | Maximal inflexion point on the outline of the posterior margin between the humeral head and the medial tuberosity |
| 4 | Most medial point of the medial tuberosity in the delimitation between the proximal end and the diaphysis |
| 5 | Maximum of concavity of the anterior curve delimiting the proximal end, between the anterior face of the humeral head and the anteromedial part of the deltopectoral crest |
| 6 | End of the deltopectoral crest (break of slope in medial view) |
| 7 | Maximum of concavity on the posterior side of the limit between the distal end and the shaft |


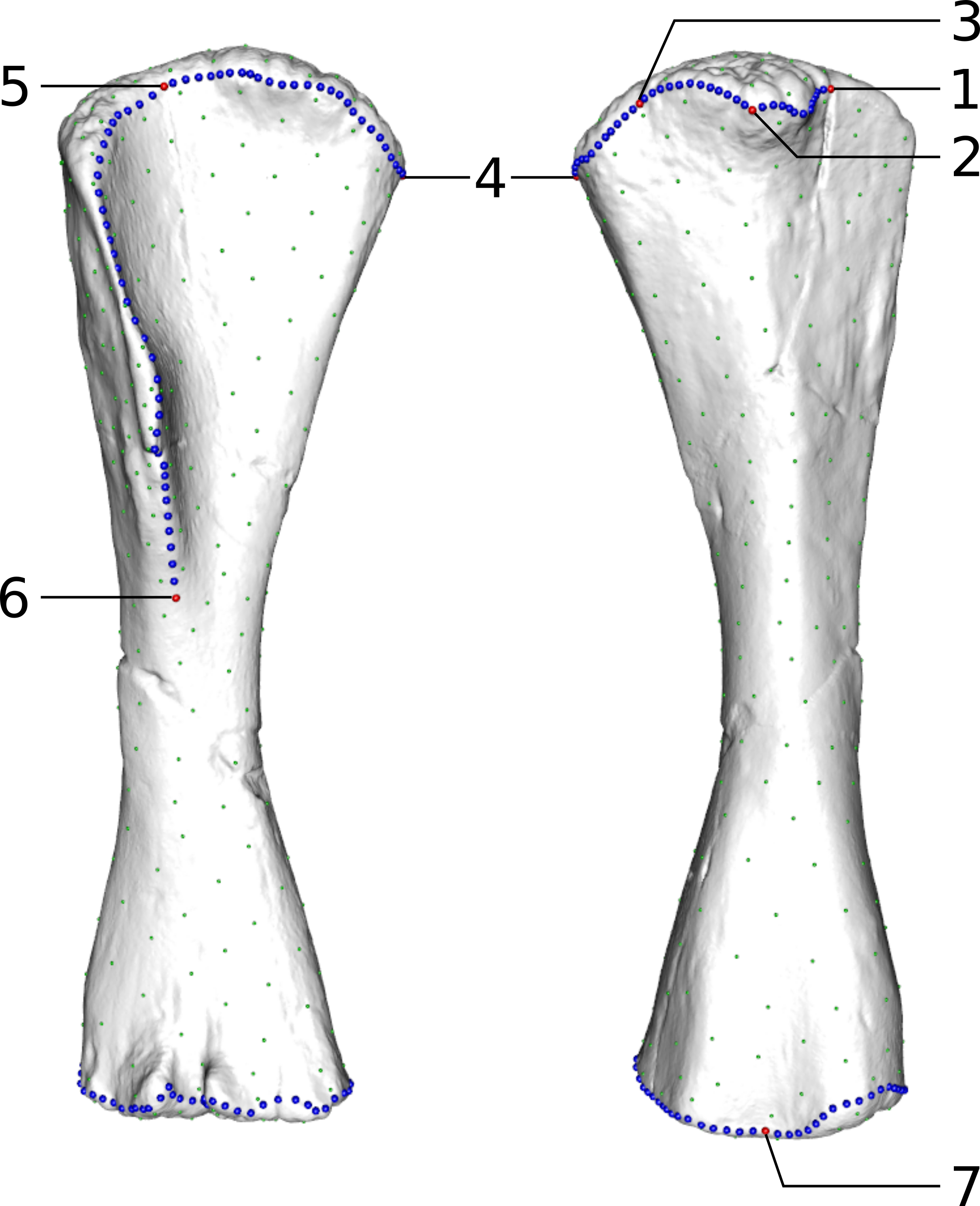


**Radius**

| # | Anatomical Landmark Definition |
| --- | --- |
| 1 | Most anteromedial point of the margin of the proximal end |
| 2 | Most posterolateral point of the margin of the proximal end |
| 3 | Apex point of the medial half margin of the distal end |


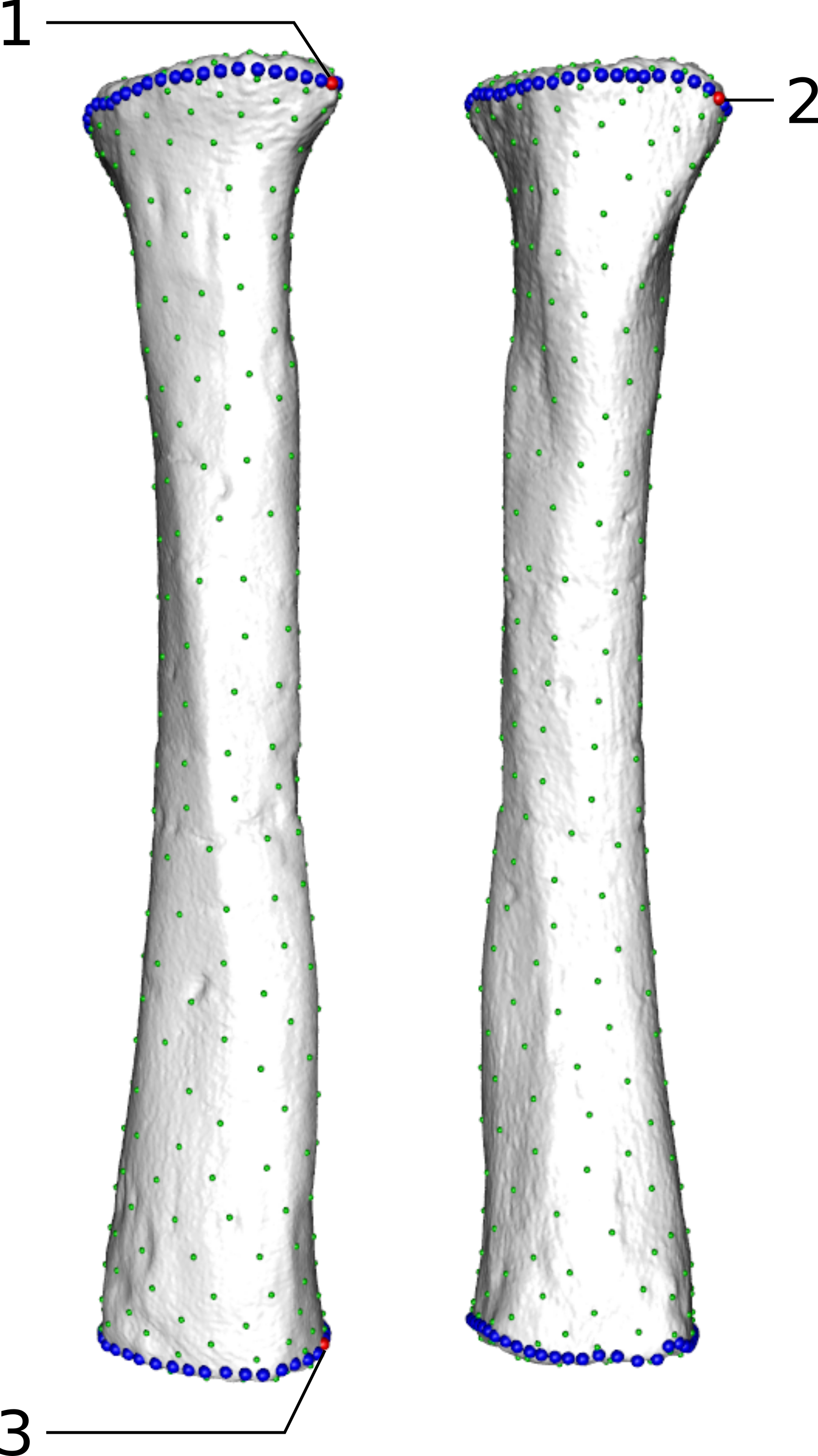


**Ulna**

| # | Anatomical Landmark Definition |
| --- | --- |
| 1 | Tip of the margin of the lateral process of the proximal end |
| 2 | Most posterior point of the margin of the olecranon |
| 3 | Maximum of curvature of the humeral cotyle on the medial side of the proximal end margin |
| 4 | Tip of the margin of the anteromedial process of the proximal end |
| 5 | Most anterolateral point of the distal end margin |
| 6 | Most posterior point of the distal end margin |


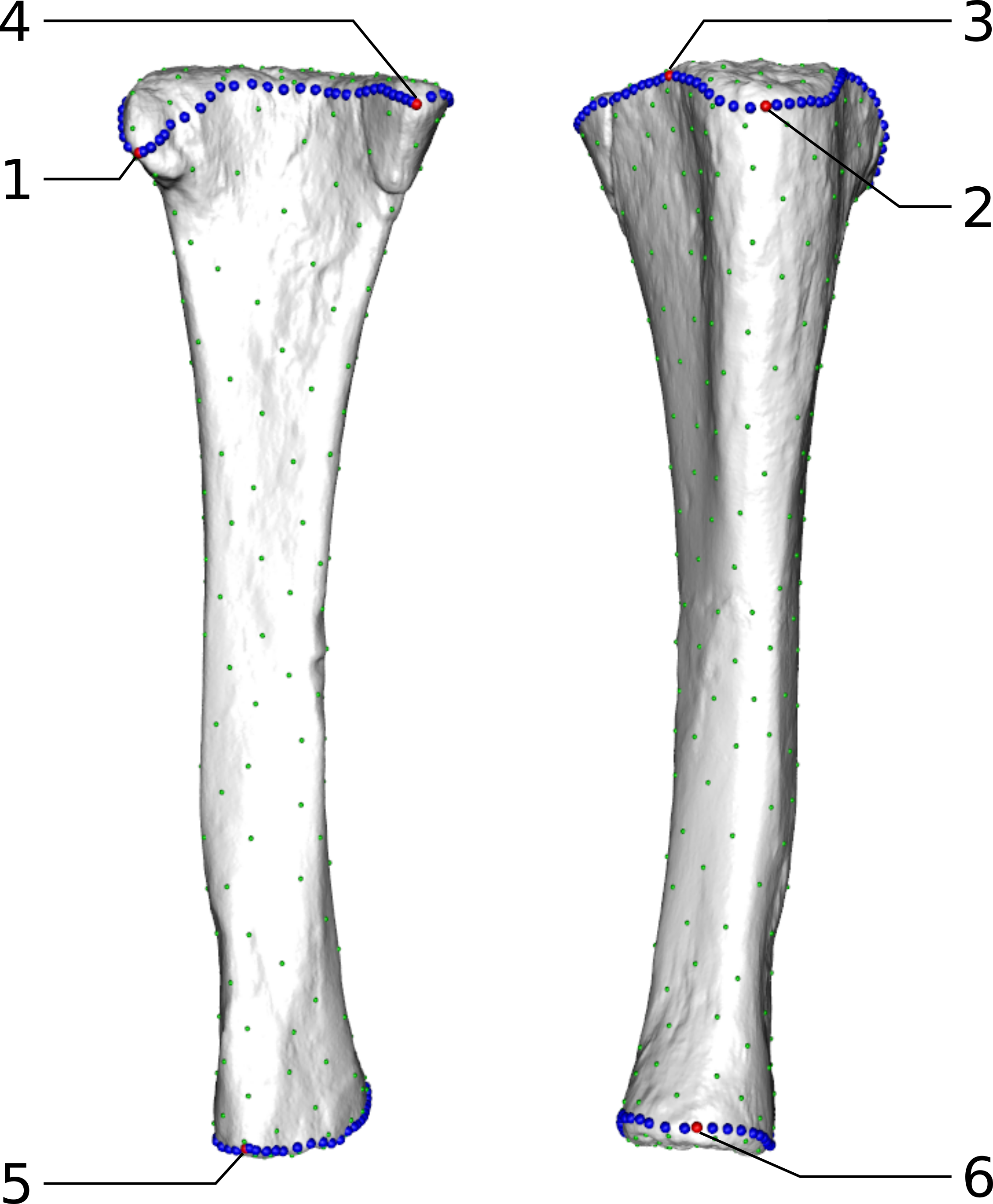


**Femur**

| # | Anatomical Landmark Definition |
| --- | --- |
| 1 | Most lateral point of the proximal end |
| 2 | Most posterior point of the proximal end |
| 3 | Most medial point of the proximal end |
| 4 | Most anterior point of the proximal end |
| 5 | Beginning of the fourth trochanter |
| 6 | End of the fourth trochanter |
| 7 | Intersection of the medial condyle and the intercondylar fossa on the margin of the distal end in the delimitation between the distal end and the shaft |
| 8 | Intersection of the lateral condyle and the intercondylar fossa on the margin of the distal end in the delimitation between the distal end and the shaft |
| 9 | Most posterior point of the lateral condyle |
| 10 | Maximum of curvature of the lateral border of the lateral condyle in the delimitation between the distal end and the diaphysis |
| 11 | Most lateral point of the distal end |
| 12 | Maximum of curvature of the anterior margin of the distal end between the two condyles |
| 13 | Maximum of curvature of the medial border of the medial condyle in the delimitation between the distal end and the diaphysis |
| 14 | Most posterior point of the medial condyle |


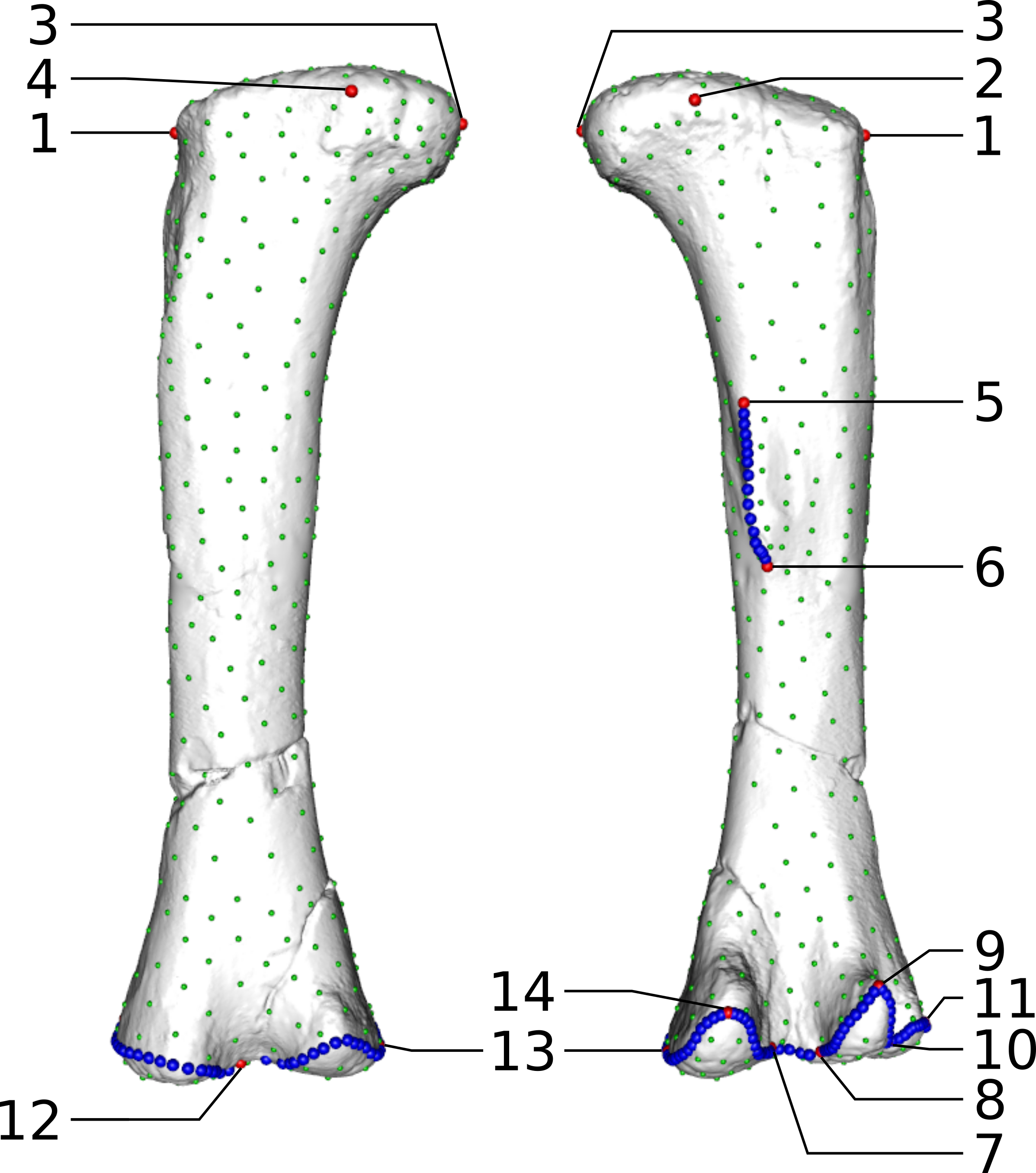


**Tibia**

| # | Landmark Definition |
| --- | --- |
| 1 | Apex of the cnemial crest pointing anterolaterally in proximal view |
| 2 | Apex of the fibular condyle pointing posterolaterally in proximal view |
| 3 | Apex of the internal condyle pointing posteromedially in proximal view |
| 4 | Intersection of the articular facet of the astragalar ascending process with the shaft, marking its beginning as an independent process on the lateral margin of the distal end |
| 5 | Most anterolateral point of the articular facet of the astragalar ascending process of the distal end |
| 6 | Tip of the anteromedial corner of the distal end |
| 7 | Tip of the posteromedial corner of the distal end |
| 8 | (Laterally oriented) Tip of the descending process |


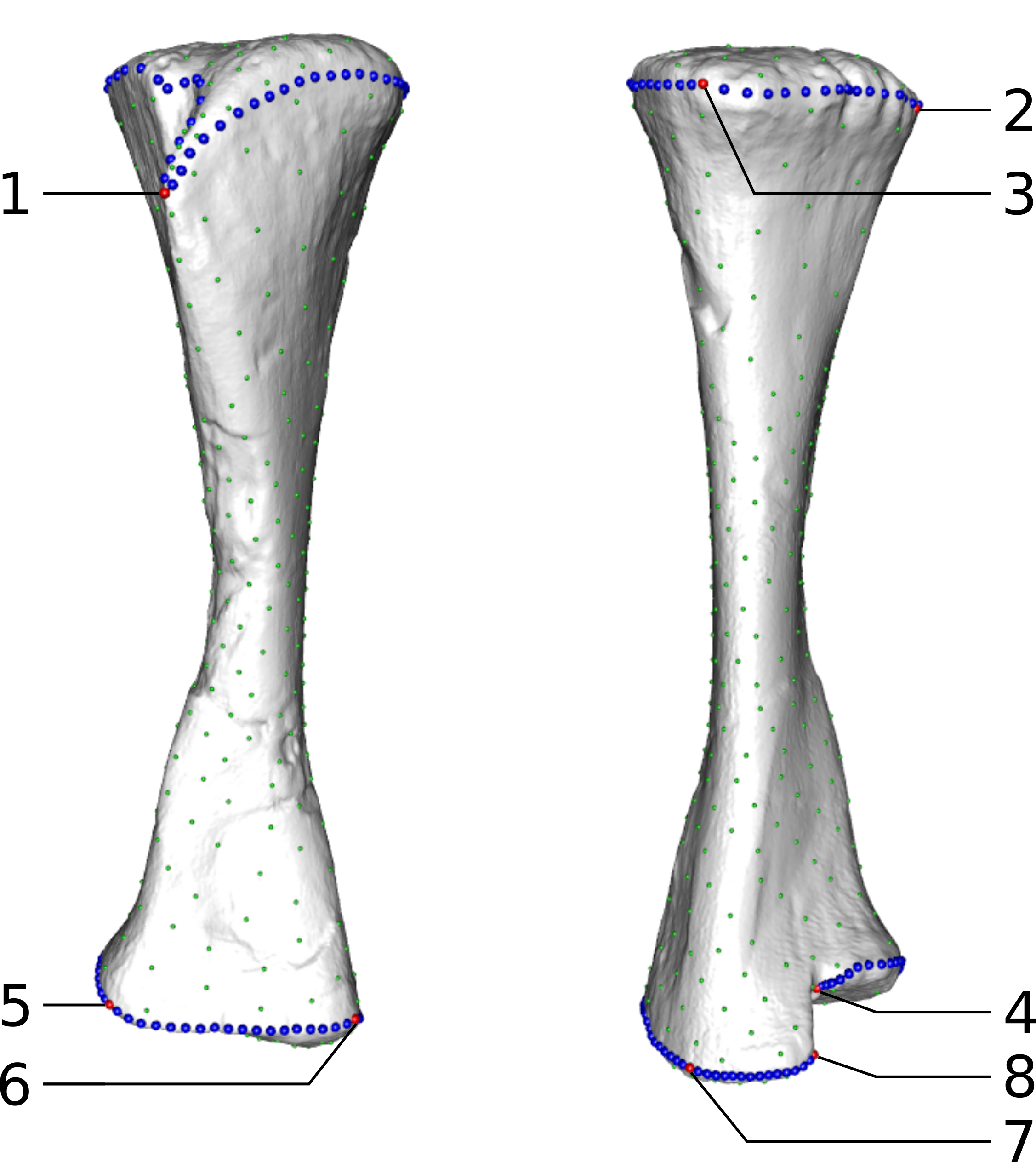


**Fibula**

| # | Landmark Definition |
| --- | --- |
| 1 | Most lateral point of the proximal end |
| 2 | Most posterior point of the proximal end |
| 3 | Maximum of concavity on the medial margin of the proximal end |
| 4 | Most anterior point of the proximal end |
| 5 | Tip of the anterior half of the distal end |
| 6 | Most medial point of the distal end |
| 7 | Most lateral point of the distal end |
| 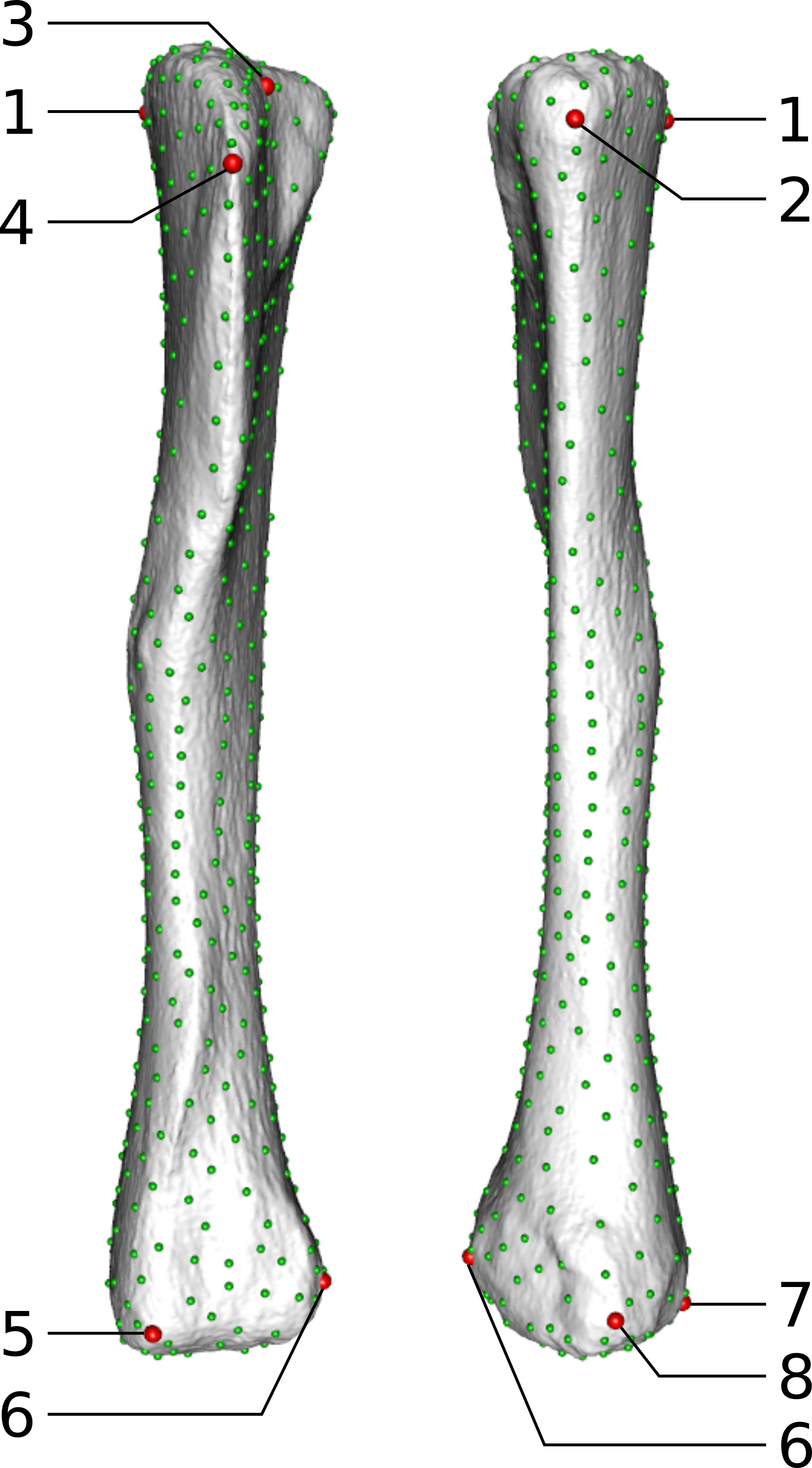8 | Tip of the posterior half of the distal end |

**Table S3**

**Tests of the size effect on bone shape, taking groups into account**

Test of the effect of size (*i.e.* natural logarithm of centroid size) on aligned landmark coordinates taking groups into account (Procrustes ANOVA with the logarithm of centroid size as a covariate and columnar-based groups as a factor; significant results (p-value < 0.05) in bold; p-value comprised between 0.05 and 0.1 in italic).

| Bone | R² | F | Z | p-value |
| --- | --- | --- | --- | --- |
| **Humerus**  log(size)  groups  HoS test  intercept | 0.1755  0.244  0.0455 | 5.2482  7.2987  1.36 | 3.0427  3.9207  0.8976 | 0.0026  1x10^-4^  0.182  0.8177 |
| **Radius**  log(size)  groups  HoS test  intercept | 0.2069  0.2747  0.0397 | 7.3496  9.7579  1.4111 | 3.6747  4.486  1.076 | 2x10^-4^  1x10^-4^  0.1387  0.7843 |
| **Ulna**  log(size)  groups  HoS test  intercept | 0.2118  0.2080  0.0127 | 9.3338  9.1658  0.5587 | 4.1161  4.6139  -1.0151 | 1x10^-4^  1x10^-4^  0.8406  0.399 |
| **Femur**  log(size)  groups  HoS test  intercept | 0.0802  0.2377  0.0134 | 2.1595  6.3978  0.3596 | 1.6540  3.6452  -1.6589 | *0.0540*  1x10^-4^  0.9624  0.0012 |
| **Tibia**  log(size)  groups  HoS test  log(size) (non-col. sauropodomorphs)  log(size) (col.  sauropods) | 0.1143  0.23551  0.12817  0.2282  0.2516 | 4.5989  9.4742  5.1562  3.8439  2.689 | 2.732  3.9097  2.9444  2.3758  1.9023 | 0.0021  1x10^-4^  0.0033  0.0167  0.0381 |
| **Fibula**  log(size)  groups  HoS test  log(size) (non-col. sauropodomorphs)  log(size) (col.  sauropods) | 0.059  0.2262  0.0855  0.1046  0.1961 | 1.8735  7.1879  2.7165  1.2844  2.1955 | 1.4649  4.0228  2.2849  0.6945  1.838 | *0.0806*  1x10^-4^  0.0141  0.2179  0.0221 |


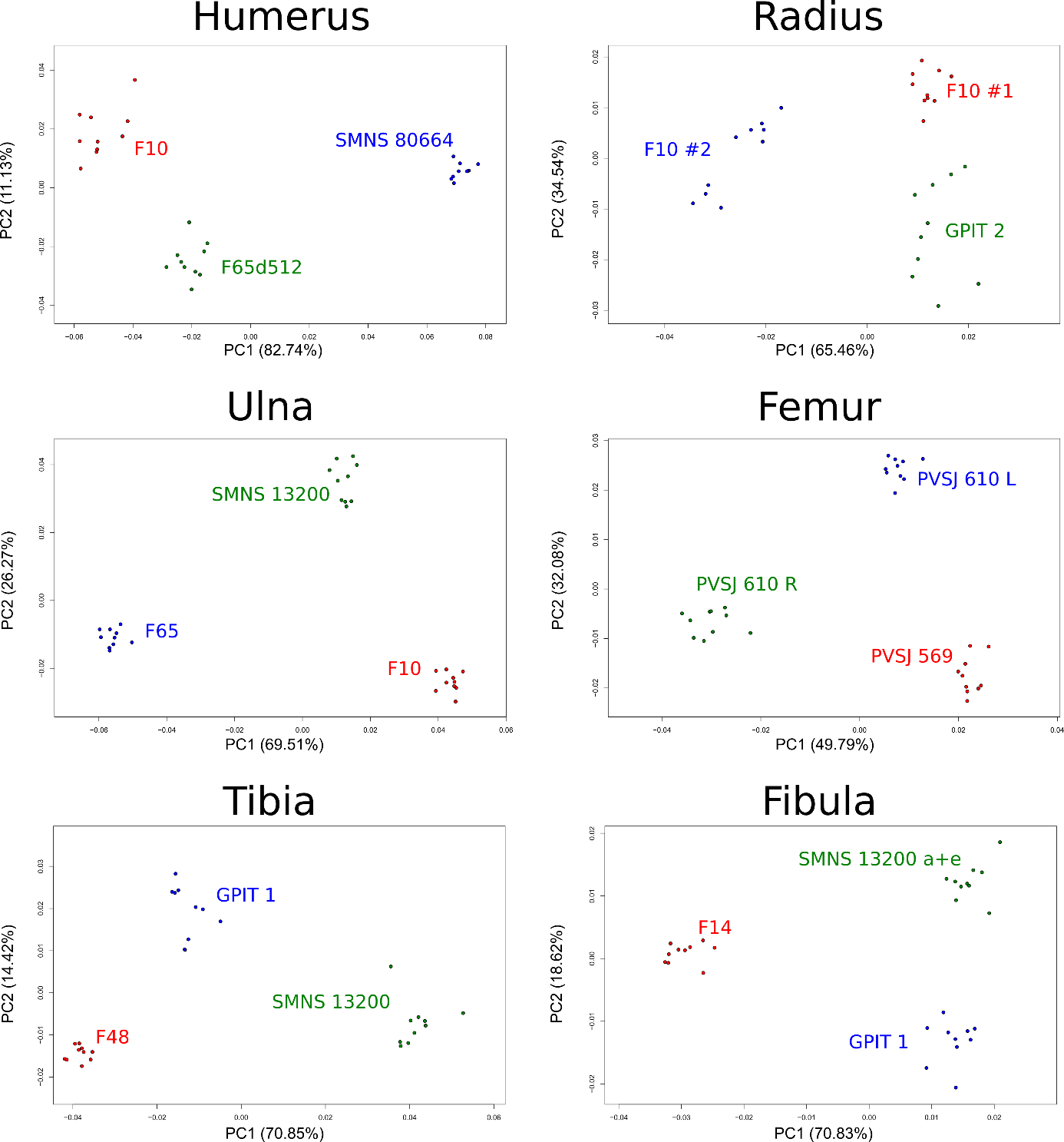


**Figure S1** Results of the PCA on the PC1 and PC2 of the repeatability procedures. For each bone, three specimens were selected. The set of anatomical landmarks of those specimens has been digitized ten times. On each plot, each specimen is recognizable by its color. For all the bones, the inter-specimen variation was greater than the intra-specimen variation. (For the femur, only one bone of PVSJ 610 was included in the study in order to avoid pseudoreplication).
